# Discovery of potent and selective activity-based probes (ABPs) for the deubiquitinating enzyme USP30†

**DOI:** 10.1039/d4cb00029c

**Published:** 2024-03-13

**Authors:** Milon Mondal, Fangyuan Cao, Daniel Conole, Holger W. Auner, Edward W. Tate

**Affiliations:** a Department of Chemistry, Molecular Sciences Research Hub, Imperial College London 82 Wood Lane London W12 0BZ UK e.tate@imperial.ac.uk; b Department of Immunology and Inflammation, Imperial College London Du Cane Road London W12 0NN UK

## Abstract

Ubiquitin-specific protease 30 (USP30) is a deubiquitinating enzyme (DUB) localized at the mitochondrial outer membrane and involved in PINK1/Parkin-mediated mitophagy, pexophagy, BAX/BAK-dependent apoptosis, and IKKβ-USP30-ACLY-regulated lipogenesis/tumorigenesis. A USP30 inhibitor, MTX652, has recently entered clinical trials as a potential treatment for mitochondrial dysfunction. Small molecule activity-based probes (ABPs) for DUBs have recently emerged as powerful tools for in-cell inhibitor screening and DUB activity analysis, and here, we report the first small molecule ABPs (IMP-2587 and IMP-2586) which can profile USP30 activity in cells. Target engagement studies demonstrate that IMP-2587 and IMP-2586 engage active USP30 at nanomolar concentration after only 10 min incubation time in intact cells, dependent on the presence of the USP30 catalytic cysteine. Interestingly, proteomics analyses revealed that DESI1 and DESI2, small ubiquitin-related modifier (SUMO) proteases, can also be engaged by these probes, further suggesting a novel approach to develop DESI ABPs.

## Abstract 2

IMP-2586 and IMP-2587 are selective and potent small molecule USP30 activity-based probes that engage USP30 rapidly in intact cells.

## Graphical abstract

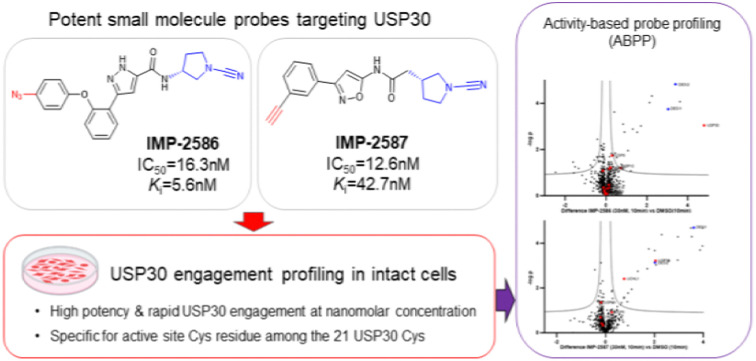

## Introduction

Decoration of proteins with the small protein modifier ubiquitin (Ub) is a reversible post translation modification (PTM) that regulates almost all cellular functions including proteolysis, transcriptional regulation, cellular trafficking, localization, inflammation, and autophagy.^1^ Hundreds of Ub ligases and deubiquitinases (DUBs) are involved in the addition or removal of ubiquitin, respectively, and modulation of this system has emerged as an important therapeutic strategy across many diseases.^2^ Ubiquitin specific peptidase 30 (USP30) is a member of the USP DUB family, which typically harbours a Cys-His-Asp catalytic triad.^3^ USP30 is the only DUB known to be present in the outer mitochondrial membrane due to its unique transmembrane domain.^4^ USP30 deubiquitylates specific mitochondrial proteins preferentially by cleaving Lys6-linked ubiquitin chains and opposes mitophagy driven by the E3 ligase Parkin.^5–8^ It has recently been shown that USP30 can also antagonise basal mitophagy mediated by the Parkinson's disease-associated kinase PINK1, even in cells which do not express Parkin.^9^ Overexpression of USP30 and dysregulation in mitochondrial turnover has been associated with neurodegenerative diseases including Parkinson's disease, Alzheimer's disease and motor neuron disease.^10–12^ USP30 may also play a role in drug resistant lymphoma, leukaemia, multiple myeloma, and BAX/BAK-dependent apoptosis.^3^ USP30 depletion sensitizes cancer cells to BH3-mimetics (*e.g.*, ABT-737), making it a potential target for cancer therapy.^8^ The USP30 inhibitor MTX652 entered clinical trials recently for Acute Kidney Injury, after exhibiting protective outcomes in various preclinical models.^13^ Despite being important therapeutic target and ongoing clinical trials of USP30 inhibitors, the endogenous substrates of USP30 have yet to be firmly established *in vivo* and regulation of its activity remains only partly understood.

Ub-derived activity-based probes (Ub-ABPs) bearing varied electrophilic warheads have been explored to better understand the function and mechanism of DUBs as well as for DUB inhibitor screening.^11^ While these Ub-ABPs are widely used and have greatly expanded our knowledge of DUB biology, they can be applied only in cell lysate due to their lack of cell permeability.^14^ In contrast, ABPs based on small molecules offer complementary properties since they are readily cell permeable, and whilst selective probes are challenging to develop they can be powerful tools to profile DUB activity in live cells, which is helpful to uncover their function in health and disease.^15,16^ Moreover, they have the potential to identify off-targets of structurally-related DUB inhibitors, which is not possible with highly DUB-specific Ub-ABPs.^17^

Several USP30 inhibitors based on *N*-cyanopyrrolidine (CNPy), oxospiramilactone, benzenesulfonamides, and naphthylsulfonamide have been reported recently,^18–20^ and in the recent patent literature.^21–25^ However, no ABP specific for USP30 has been developed to date, limiting opportunities to profile USP30 activity in intact cells. Here we report the first small molecule ABPs which target USP30 in an activity-dependent manner and demonstrate their application to identify active DUBs in cancer cells by activity-based protein profiling (ABPP).

## Results

**Fig. 1:**
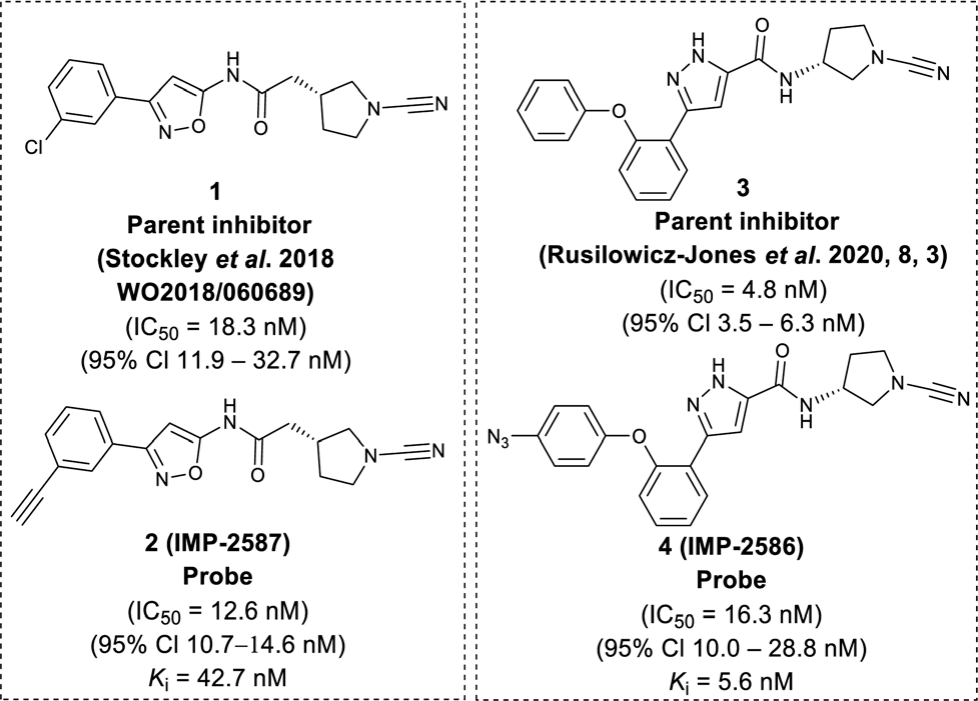
Chemical structure of parent compounds and activity-based probes with their determined IC_50_ and *K*_i_ accordingly. Fluorescent Polarisation assay were performed for IC_50_ (30 minutes incubation against USP30), *k*_obs_/*I*, and *k*_inact_/*K*_i_ determination of IMP-2587, and IMP-2586 against USP30 using Ub-Lys(TAMRA)-Gly (Fig. S1, ESI†).

**Scheme 1:**
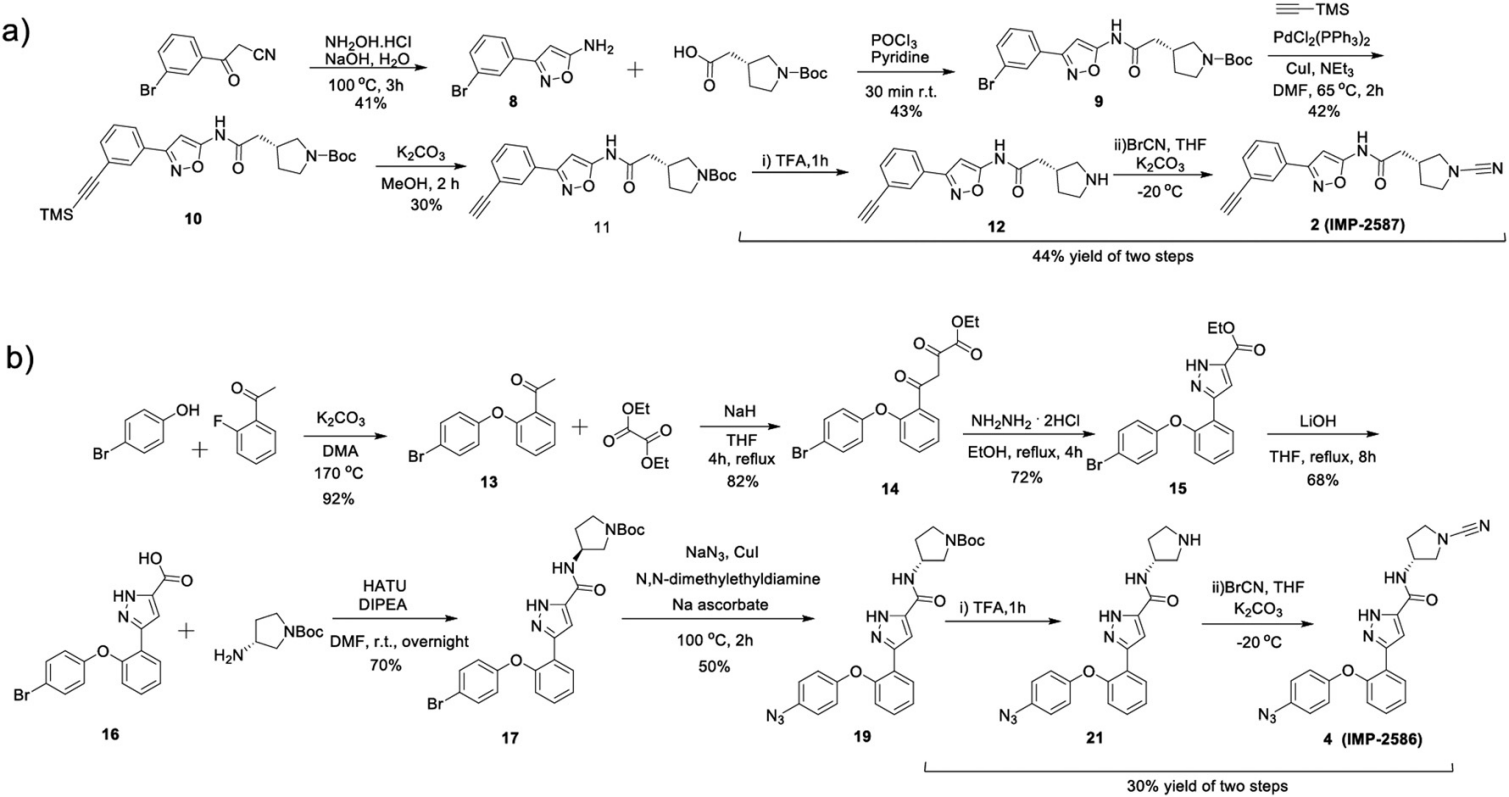
Synthetic route to the compounds described in this work. (a) synthetic route to compound 2 (IMP-2587); (b) synthetic route to compound 4 (IMP-2586).

To identify a suitable ABP scaffold for USP30, we considered several reported covalent USP30 inhibitors with low nanomolar potency, prioritising those bearing a CNPy warhead, expected to react with the DUB active cite cysteine residue to form an isothiourea adduct.^16,26^ We synthesised CNPy 1 (Fig. 1 and Scheme S1, ESI†) which has a reported IC_50_ of 1–10 nM against USP30^25^ (following 30 minutes incubation), and designed ABP (2, which we named IMP-2587) (Fig. 1 and Scheme 1) bearing a terminal alkyne functionality, based on reported structure–activity relationship (SAR) data suggesting that *meta* substitution on the aryl ring would retain activity, and compatibility with bioorthogonal click chemistry. IMP-2587 was synthesised through a short synthetic route (Scheme 1a). In parallel, we explored a CNPy with a different scaffold, synthesising the highly potent CNPy-bearing covalent inhibitor 3 (Fig. 1 and Scheme S1, ESI†) with reported IC_50_ value = 1.5 nM against USP30^18^ (following 30 minutes incubation), and high biochemical selectivity for USP30 among a diverse set of DUB family members, although with some appreciable off-target specificity against USP6 at higher concentration.^18^ We designed ABP IMP-2586 based on 3. For installation of the affinity handle (azide), the reported SAR supported *para* substitution at the terminal aryl ring, so we synthesised 4 by a short synthetic route (Scheme 1b). An azide bioorthogonal tag was preferred in this case due to the problematic instability of an alkyne in this relatively electron rich position during pyrrolidine Boc deprotection.

We first confirmed biochemical inhibition of USP30 with our probes in a fluorescence polarisation (FP) assay using a TAMRA labelled Lys-Gly dipeptide linked to ubiquitin *via* an isopeptide bond (Ub-Lys(TAMRA)-Gly). USP30 (5 nM) was incubated across a concentration series of test compound for 30 min, followed by FP measurement after addition of Ub-Lys(TAMRA)-Gly (100 nM). IMP-2587 showed IC_50_ value of 12.6 nM (95% CI 10.7–14.7 nM) against USP30, whereas parent inhibitor 1 showed IC_50_ value of 18.3 nM (95% CI 11.9–32.7 nM). IMP-2586 displayed IC_50_ value of 16.3 nM (95% CI 10.0–28.8 nM), while the original inhibitor 3 showed IC_50_ value of 4.8 nM (95% CI 3.5–6.3 nM) (Fig. 1 and Fig. S1, ESI†). Cross-screening of IMP-2587 and IMP-2586 against an unrelated DUB (UCHL1) displayed no activity (Table S1, ESI†).

We further characterised inhibition kinetics for ABPs in a FP assay using Ub-Lys(TAMRA)-Gly. *k*_obs_/*I*, *K*_i_ and *k*_inact_ determination assay was performed using 30 nM USP30 and 100 nM Ub-Lys(TAMRA)-Gly as described in ESI,† Section S3.1.c. FP was monitored kinetically over an hour after addition of the substrate, and response curves fitted to *y* = (*v*_i_/*k*_obs_) (1 − exp(−*k*_obs_*x*)) to calculate *k*_obs_/I, 5680 (95% Cl = 4723–6638) M^−1^ s^−1^ for IMP-2587, and 3021 (95% Cl = 2350–3693) M^−1^ s^−1^ for IMP-2586 (Fig. S1, ESI†). *K*_i_ and *k*_inact_ were estimated by nonlinear regression to *k*_obs_ = *k*_inact_/(1 + (*K*_i_/*x*)) for probes IMP-2587 (*K*_i_ 42.8 nM, *k*_inact_ 0.0014 s^−1^) and IMP-2586 (*K*_i_ 5.6 nM and *k*_inact_ 0.00067 s^−1^), confirming robust covalent inhibition activity and affinity for these ABPs (Fig. S1, ESI†).

**Fig. 2:**
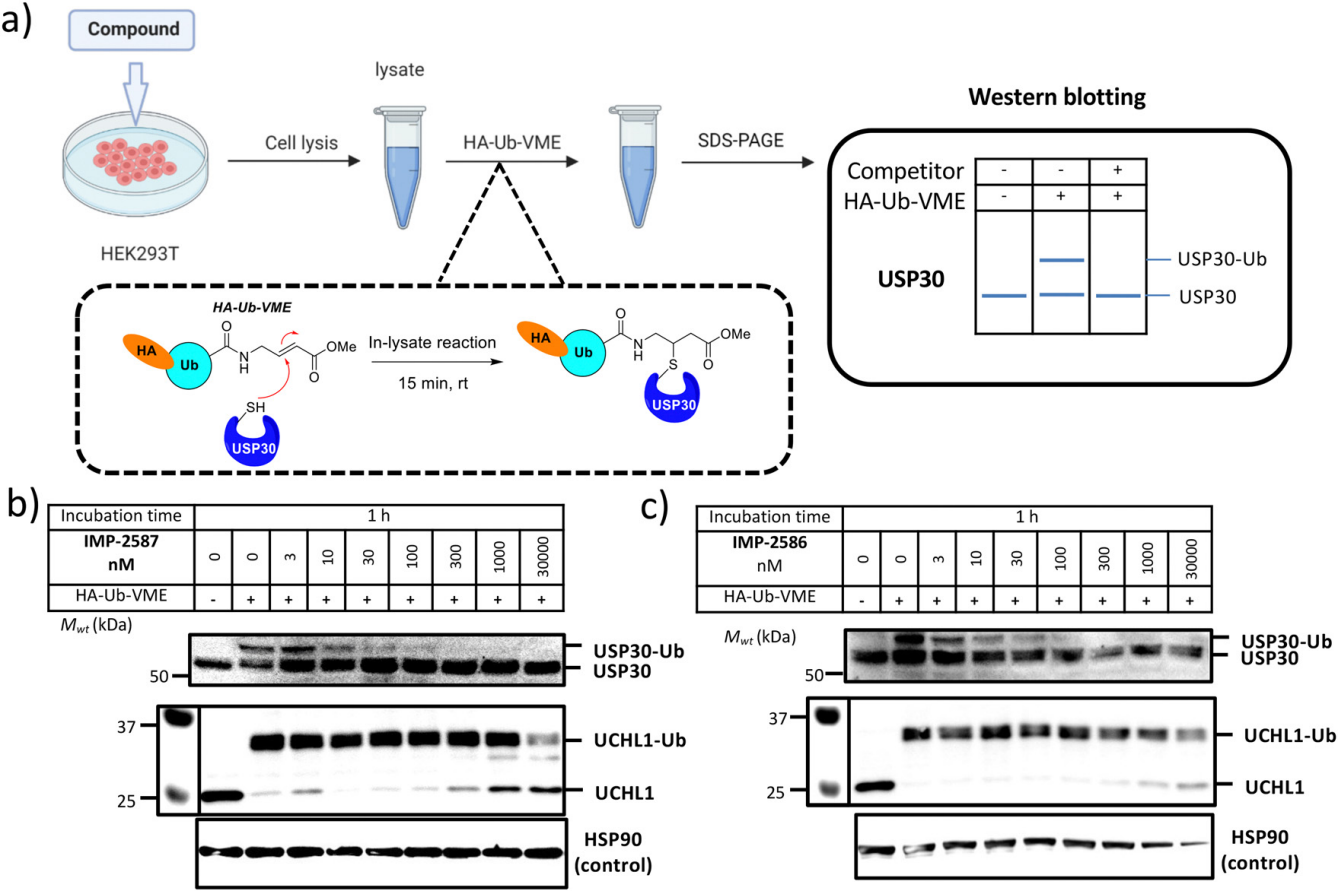
(a) Workflow of immunoblot analysis of HA-Ub-VME USP30 labelling following HEK293T treatment with IMP-2587 (b) and IMP-2586 (c) for 1 h. Dose-dependent competition for USP30 labelling occurs for both the ABPs but not with UCHL1 at similar concentration range.

**Fig. 3:**
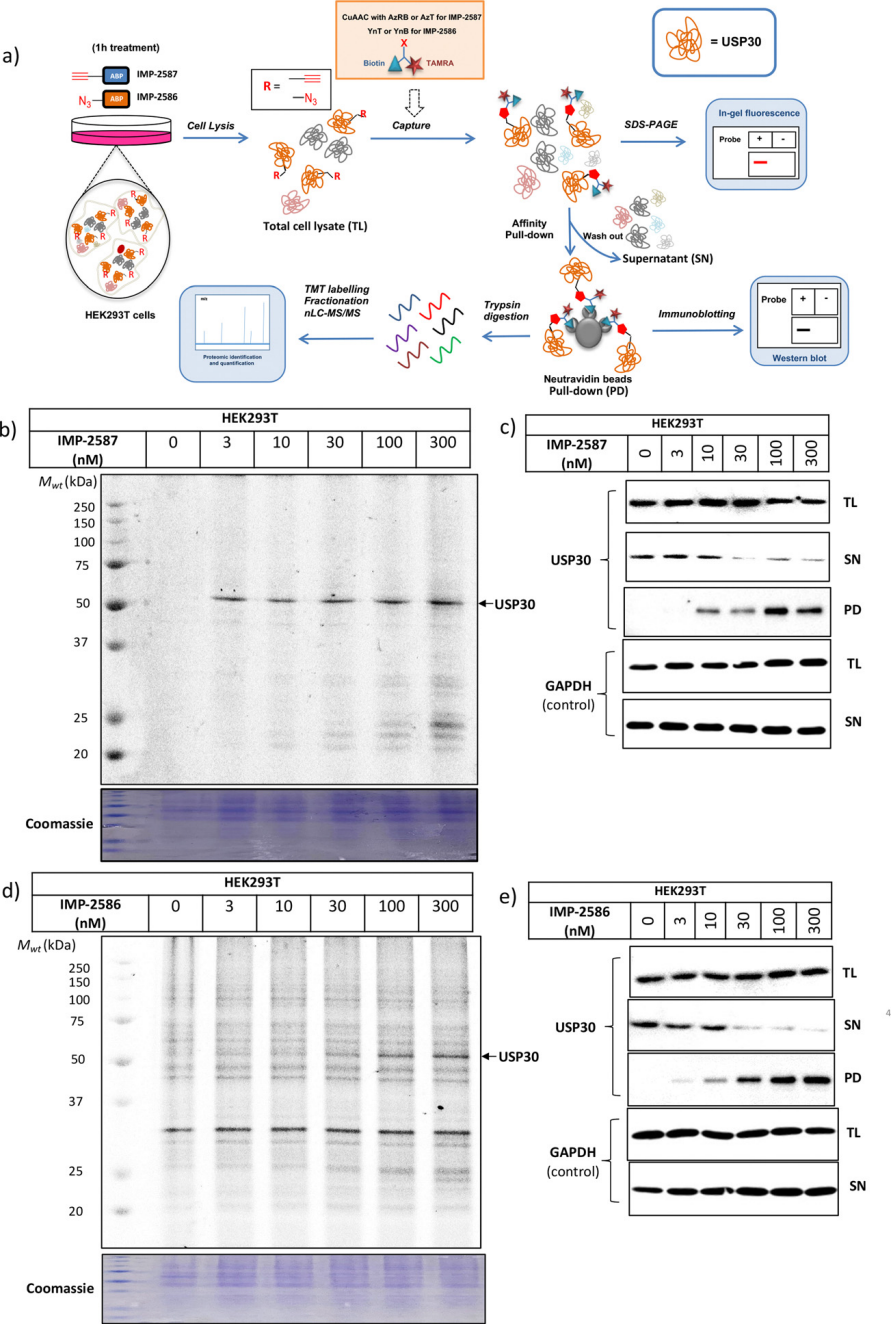
USP30 activity profiling using IMP-2587 and IMP-2586 in HEK293 cells. (a) Affinity pulldown and chemical proteomics workflow. Cells were incubated with IMP-2587 and IMP-2586 for 1 hour, separately and labelled proteins ligated *via* CuAAC to AzRB or AzT for IMP-2587 and YnB or YnT for IMP-2586 for in-gel fluorescence and/or affinity enrichment for immunoblotting and proteomics. Total cell lysates are labelled as TL samples; supernatants following affinity enrichment were collected as SN samples; protein bound to the beads was collected as PD samples. (b) In-gel fluorescence shows dose-dependent labelling by IMP-2587. (c) Probe-labelled (IMP-2587) protein identification by enrichment and immunoblotting for USP30. (TL: Total Lysate, SN: Supernatant, PD: Pull-Down) (d) In-gel fluorescence shows dose-dependent labelling by IMP-2586. (e) Probe-labelled (IMP-2586) protein identification by enrichment and immunoblotting for USP30.

We next determined cellular engagement of endogenous USP30 with IMP-2587 or IMP-2586. HEK293T cells were treated with ABPs for 1 hour followed by western blot analysis of in-lysate competitive activity-based profiling against hemagglutinin-tagged Ub-vinyl methyl ester probe (HA-Ub-VME) (Fig. 2a). In-cell concentration-dependent competition by IMP-2587 and IMP-2586 for USP30 were confirmed below 100 nM probe (Fig. 2b and c), whilst engagement of an exemplar unrelated DUB, UCHL1, occurred only at a much higher concentration (30 000 nM). We hypothesise that incomplete modification of USP30 by HA-Ub-VME is likely due to limited retention of USP30 activity in cell lysates, which have likely had the USP30 transmembrane domain compromised, highlighting the potential utility of a small molecule USP30 ABP applicable directly in intact cells. Direct USP30 target engagement by IMP-2587 and IMP-2586 was confirmed in HEK293T cells following 1 h probe treatment followed by cell lysis and CuAAC bio-orthogonal ligation to capture reagent azido-TAMRA-biotin (AzTB) for IMP-2587 or alkyne-TAMRA-biotin for IMP-2586 (Fig. 3a).^27^ In-gel fluorescence revealed one major band at *ca.* 60 kDa for IMP-2587 starting as low as 3 nM probe, with some labelling of additional bands above 30 nM (Fig. 3b). Similarly, labelling with IMP-2586 revealed a major band at *ca.* 60 kDa, but in this case in-gel fluorescence showed a degree of unspecific labelling across all concentrations, including the vehicle (DMSO)-treated control (Fig. 3d). This unspecific background labelling is presumably due to the previously reported higher off-target CuAAC reactivity of an alkyne-bearing capture reagent, applied here due to incorporation of an azide tag in IMP-2586.^28^ Moreover, the labelling of USP30 was further confirmed by biotin pulldown and immunoblotting. USP30 was enriched at 10 nM probe and maximal at 100 nM, resulting in almost complete engagement and enrichment with both IMP-2587 and IMP-2586 (Fig. 3c and e), which was in stark contrast to the ∼50% USP30 engagement observed with the Ub-based probe, HA-Ub-VME (Fig. 2b and c).

**Fig. 4:**
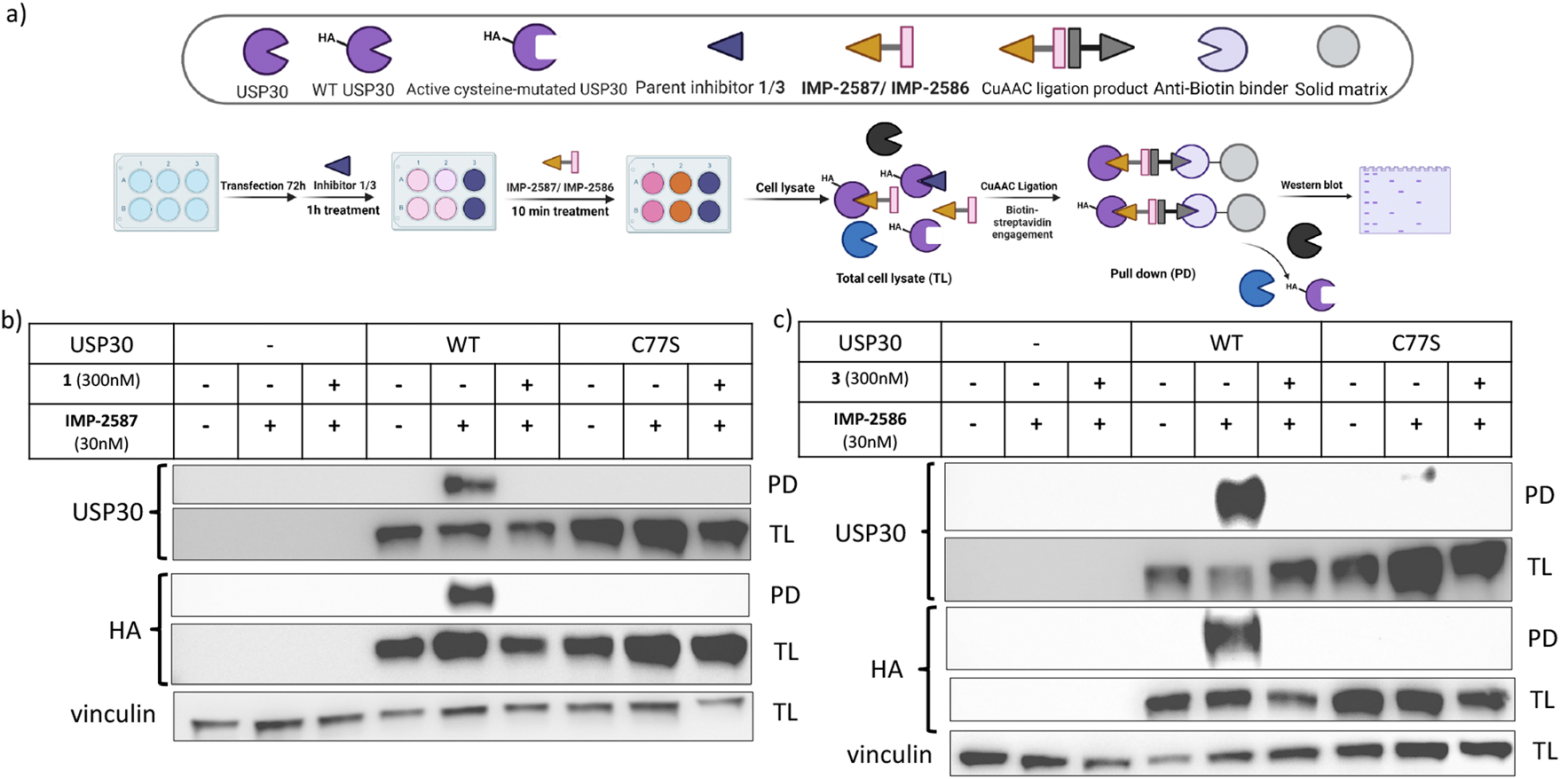
(a) Workflow of HA-tagged USP30 WT or C77S transfection system and IMP-2587/IMP-2586 labelling in HEK293T cells. HEK293T cells were transfected to overexpress HA-tagged USP 30 WT or catalytic CS mutants, and pulldown following treatment with IMP-2587 (b) or IMP-2586 (c) for 10 min. (PD: Pull-Down, TL: Total Lysate).

To validate these probes as ABPs, we sought to demonstrate dependence on USP30 catalytic activity for cellular target engagement, and specificity for the active site Cys residue among the >20 Cys residues present in USP30. We expressed HA-tagged wild-type (WT) USP30 and active site cysteine to serine mutant (CS) in HEK293T cells (Fig. 4a). Cells were treated by IMP-2587 or IMP-2586 for 10 min or pre-treated with corresponding parent inhibitors for 1 hour followed by probe treatment. Western blot analysis of affinity enriched USP30 demonstrates that USP30 labelling for both probes requires the presence of the catalytic cysteine, and this enrichment can be readily outcompeted by the parent inhibitors (Fig. 4b and c), confirming the activity-dependence of these ABPs.

**Fig. 5:**
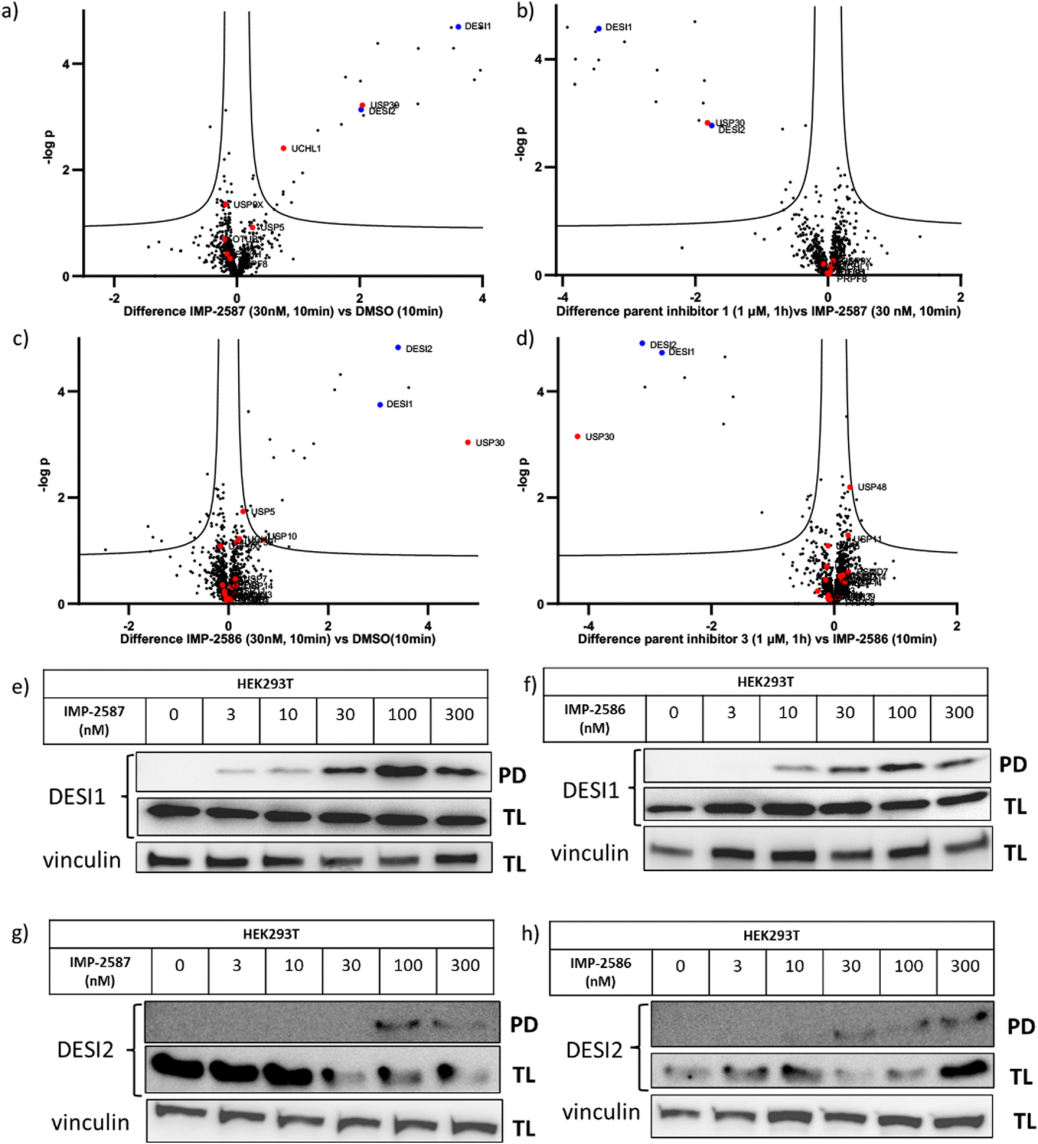
Chemical proteomic analysis of IMP-2587 at 30 nM (a) or 100 nM (b) and IMP-2586 at 30 nM (c) or 100 nM (d) labelling in HEK293T cells. Volcano plots showing log_2_ difference (fold change) and significance (−log *p*-value) between protein enrichment compared to DMSO control (two sample *t* test, *n* = 3, permutation-based FDR = 0.05, S0 = 0.1). Black dots represent non-DUBs, red dots represent DUBs and blue dots represent DESIs. Probe-labelled protein identification by enrichment and immunoblotting in HEK293T cells for DESI1 with IMP-2587 (e) and IMP-2586 (f) treatment for 10 min or DESI2 with IMP-2587 (g) and IMP-2586 (h) treatment for 10 min. (TL: Total Lysate, PD: Pull-Down).

To explore the selectivity of IMP-2587 and IMP-2586 across the whole proteome, we performed quantitative activity-based protein profiling (ABPP) in HEK293T cells following treatment with IMP-2587 or IMP-2586 (Fig. 3a). After ABP incubation, cells were lysed, and probe-labelled proteins were ligated to biotinylated capture reagents.^27^ Incubation of cells with 30 nM probe for 10 min showed highly significant enrichment of USP30 (Fig. 5a and c and Tables S2 and S4, ESI†), consistent with results from in-gel fluorescence (Fig. 3) and pull-down data (Fig. 3 and 4). USP30 was outcompeted by corresponding parent inhibitors for each ABP (Fig. 5b and d and Tables S3 and S5, ESI†), confirming the high potency and rapid engagement of IMP-2587 and IMP-2586 in cells. Interestingly, we also observed less potent but still significant enrichment of two desumoylating isopeptidases (DESI1 and DESI2), which were further validated by pull-down immunoblot analyses (Fig. 5e–h). DESI1 and DESI2 are small ubiquitin-related modifier (SUMO) proteases,^3,29–32^ and IMP-2587 and IMP-2586 may offer a starting point for future development of DESI ABPs.

## Discussion

Small molecule DUB ABPs circumvent the limitations of Ub-based ABPs, which can only be applied in cell lysates. Cell lysis may disrupt native DUB activity resulting in inconsistent and potentially misleading engagement by Ub-ABPs, as seen in the present study in the incomplete modification of USP30 by HA-Ub-VME. In contrast, recently developed pan-DUB small molecule ABPs enable exploration of DUB activity in intact cells, and assessment of cellular target engagement and selectivity of novel inhibitors.^15,33^

Dysregulation of USP30 is implicated in a range of rare genetic mitochondrial diseases, and neurodegenerative diseases including Parkinson's disease.^12^ Accordingly, there is significant interest in developing USP30 inhibitors for the clinic, and Mission Therapeutics initiated the first clinical trials of a USP30 inhibitor in 2022 for muscular, cardiac and kidney pathologies,^34^ reporting encouraging Phase I safety data. Selective and potent small molecule USP30 ABPs would be useful tools to explore the role of USP30 activity in intact cells for these diseases.

In this work, we designed, synthesised and validated two novel USP30 ABPs bearing a CNPy. Cyanoamines, particularly CNPy, have been established as a privileged warhead class for DUBs, which have proven challenging to target selectively with other warhead classes (*e.g.* chloroacetamides).^3,16,26,33,35,36^ Recent reports have shown that minimal CNPy probes lacking an extended structure lose activity toward DUBs, suggesting that the scaffold beyond the warhead plays a key role in enabling covalent DUB active site modification, and in selectivity within the DUB family.^26^ Interestingly, IMP-2586 and IMP-2587 showed very similar selectivity for USP30 in cells despite their divergent scaffolds. Unlike most reported DUB ABPs, IMP-2586 bears an azide tag rather than a terminal alkyne. Given the prevalence and convenience of Boc protecting group chemistry in CNPy synthetic routes, this design choice overcomes problems of acid instability of an electron rich alkyne in this position. Despite reduced CuAAC bioorthogonal ligation specificity using an alkyne capture reagent (Fig. 3), the utility of IMP-2586 as a selective USP30 ABP is preserved in cellular studies.

Our data suggest that both parent inhibitors from which IMP-2587 and IMP-2586 are derived also target DESI1 and DESI2 in cells and these may need to be considered as off-targets where related compounds are used as inhibitors. Like DUBs, DESIs are cysteine proteases, but catalyse hydrolysis of SUMO modifications rather than Ub.^29^ No probe or inhibitor has been reported to date for DESIs, although these enzymes are reported to be involved in a wide range of cellular pathways, including PI3K/AKT/mTOR signalling and P53-induced apoptosis.^30,31^ Future optimisation of IMP-2586 and IMP-2587 to minimise USP30 activity may provide the first ABPs for studying DESIs.

## Conclusions

IMP-2586 and IMP-2587 enable sensitive and rapid detection of USP30 activity in intact cells at probe concentrations as low as 3–10 nM following 1 hour incubation, or 30 nM at 10 min incubation. Parent inhibitors 1 and 3, IMP-2587 and IMP-2586, also showed high inhibitory potency against USP30 with low nM IC_50_ and fast USP30 modification kinetics both biochemically and in cells. Low nanomolar cellular target engagement and selectivity were examined with a range of orthogonal approaches, including HA-Ub-VME ABP assays, and analysis of direct target engagement through CuAAC bio-orthogonal ligation, in-gel fluorescence, enrichment, immunoblotting and ABPP proteomics. Both probes are strictly activity-based and selective for the active site cysteine of USP30 for labelling among the 21 Cys residues present in USP30.

In summary, IMP-2586 and IMP-2587 represent the first potent and selective ABPs to explore USP30 activity in intact cells, with the potential to facilitate studies of USP30 biology and target engagement, supporting future development of appropriate therapeutic strategies.

## Data availability

The MS data from this publication have been deposited to the ProteomeXchange Consortium *via* the PRIDE partner repository and assigned the identifier PXD044792.

## Conflicts of interest

E. W. T. is a founding director and shareholder of Myricx Pharma Ltd., an advisor of and holds share options in Sasmara Therapeutics and receives current or recent funding from Myricx Pharma Ltd, Pfizer Ltd, Kura Oncology, AstraZeneca, Merck & Co., GSK.

## Supplementary Material

CB-005-D4CB00029C-s001
