# Supplementary material for: Discovery of potent and selective activity-based probes (ABPs) for the deubiquitinating enzyme USP30
Source: RSC Chem Biol. 2024 Mar 13;5(5):439–46. doi: 10.1039/d4cb00029c (PMC11078216; doi:10.1039/d4cb00029c)
Supplement: CB-005-D4CB00029C-s001 [file CB-005-D4CB00029C-s001.pdf]

Electronic Supplementary Material (ESI) for RSC Chemical Biology.
This journal is © The Royal Society of Chemistry 2024

Discovery of Potent and Selective Activity-Based Probes (ABPs) for the Deubiquitinating Enzyme USP30

Contents

1. **Supplementary scheme and figures** 2
2. **Supplementary tables** 10
3. **General synthetic methods** 17
   1) **Chemical synthesis of the probes** **17**
   2) **Chemical synthesis of the parent inhibitors** **23**
   3) **^{1}H and ^{13}C spectra** **28**
4. **Biochemical and biological methods and proteomics** 51
   1) **Biochemical USP30 fluorescence polarisation (FP) assay using Ub-Lys(TAMRA)-Gly** **51**
      a. USP30 biochemical IC_{50} assay 51
      b. USP30 biochemical kinetic assay 51
      c. Kinetic analysis 51
   2) **USP30 in-cell engagement analysis** **52**
      a) Cell culture 52
      b) In-cell target engagement using HA-Ub-VME 52
      c) SDS-PAGE and immunoblotting analysis 52
      d) Live cell probe incubation, lysate click chemistry and precipitation 53
      e) In-gel fluorescence 53
      f) Neutravidin biotin enrichment (Affinity pull-down) 53
   3) **Proteomics analysis** **54**
      a) Live cell probe incubation 54
      b) Click chemistry, precipitation, Neutravidin biotin enrichment and on-bead digestion 54
      c) Isobaric TMT labelling and high pH reverse phase fractionation 54
      d) LC-MS/MS analysis 55
      e) Database searching and proteomics data analysis 55
   4) **USP30 WT and catalytic CS overexpressed in HEK293T cells** **56**
      a) Preparation of DNA constructs 56
      b) Profiling labelling in USP30 overexpression cells 56
5. **Reference** 56

1. Supplementary scheme and figures

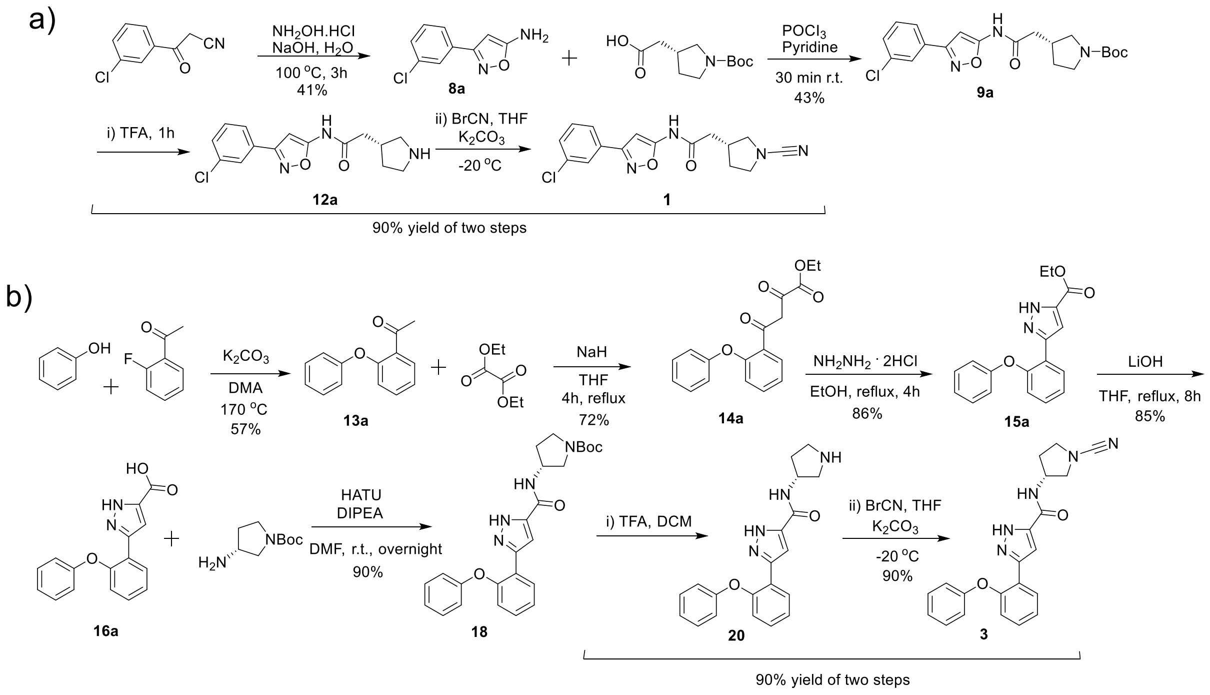

Scheme S1. Synthetic route to the parent inhibitors described in this work. a) synthetic route to compound **1**,[1] b) synthetic route to compound **3**.[2]

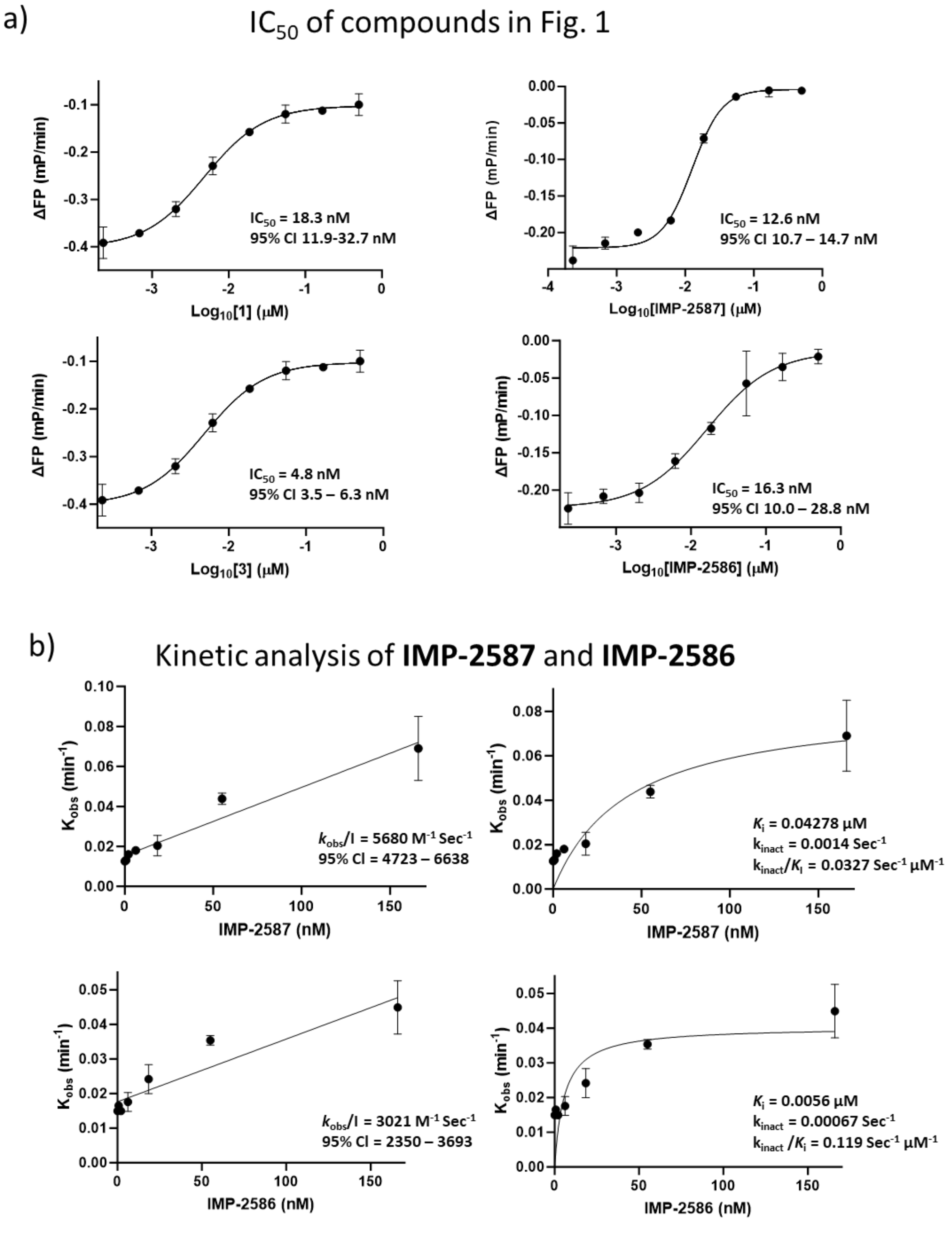

Figure S1. Fluorescent Polarisation assay for biochemical evaluation and kinetic analysis; (a)IC_{50} determination of compounds in Fig. 1; (b) k_{obs}/I, and k_{inact}/K_I determination of **IMP-2587**, and **IMP-2586** against USP30 using Ub-Lys(TAMRA)-Gly.

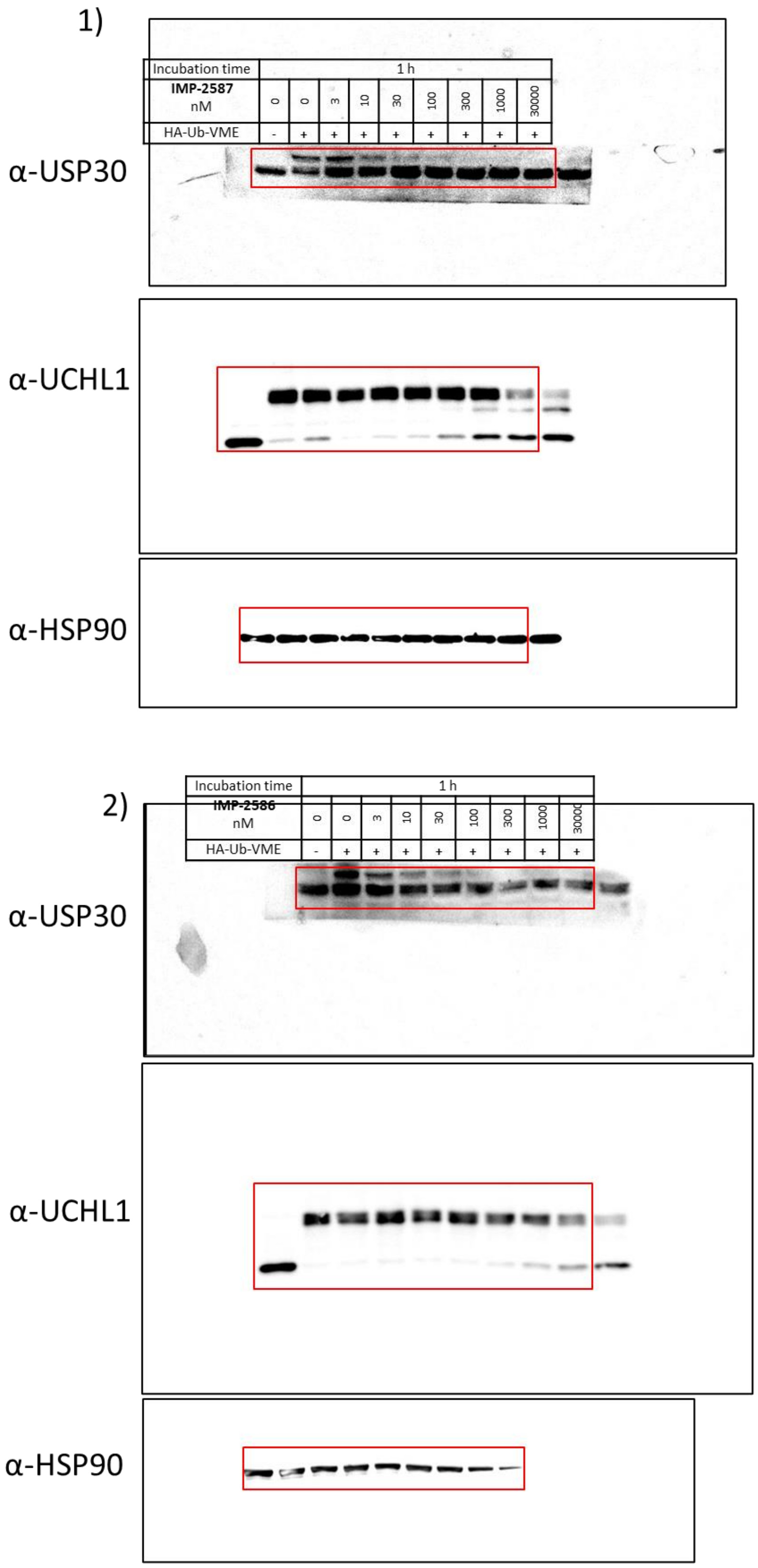

Figure S2. Uncropped blots for HA-Ub-VME competition experiments in Fig. 2. Fig. 2b displayed the bands in red box in Fig. S2-1. Fig. 3c displayed the bands in red box in Fig. S2-2.

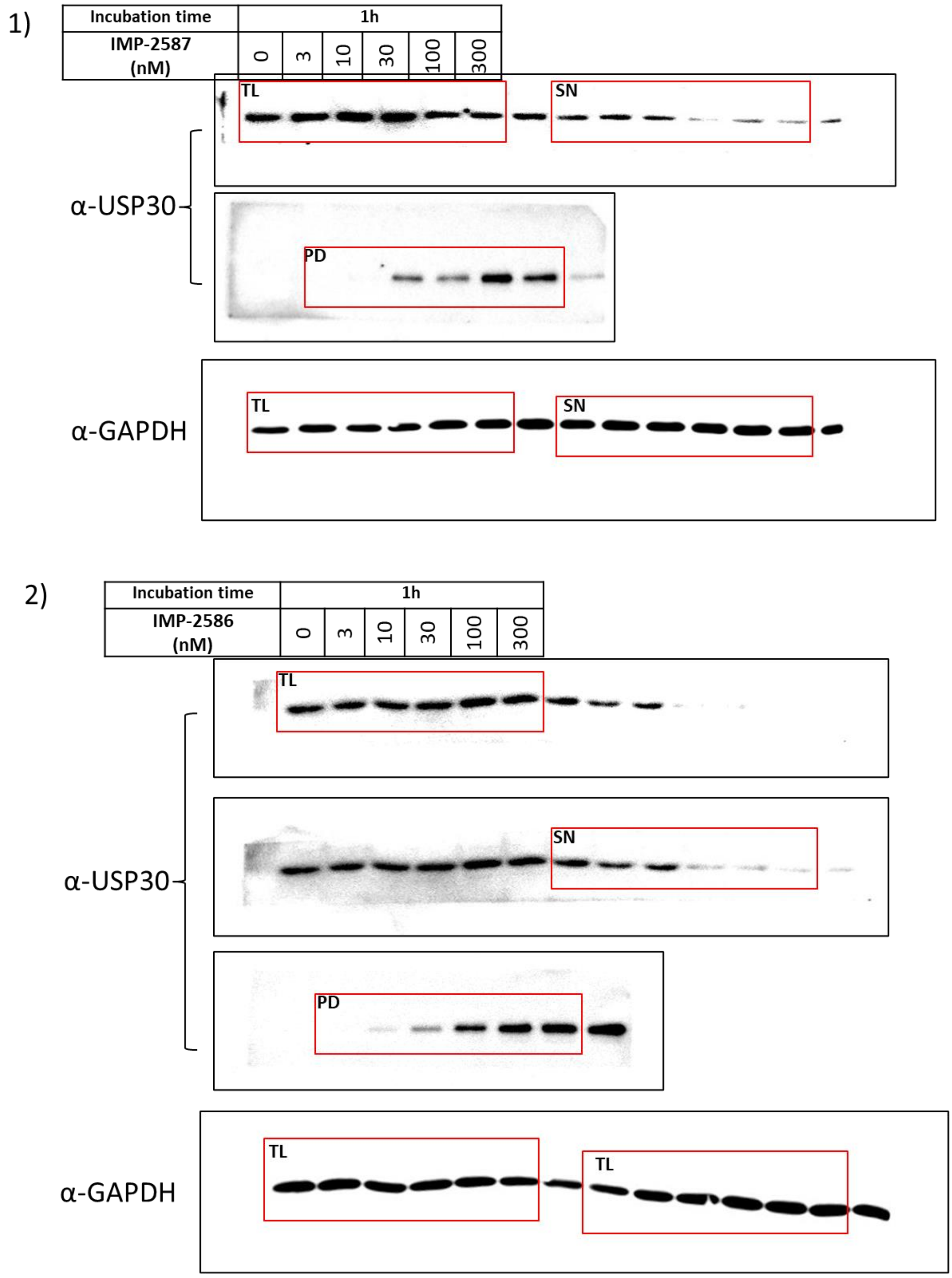

Figure S3. Uncropped blots for affinity pull-down experiments in Fig. 3. TL –Total Lysate; PD – Pull-down; SN-supernatant. Fig. 3c displayed the bands in red box in Fig. S3-1. Fig. 3e displayed the bands in red box in Fig. S3-2.

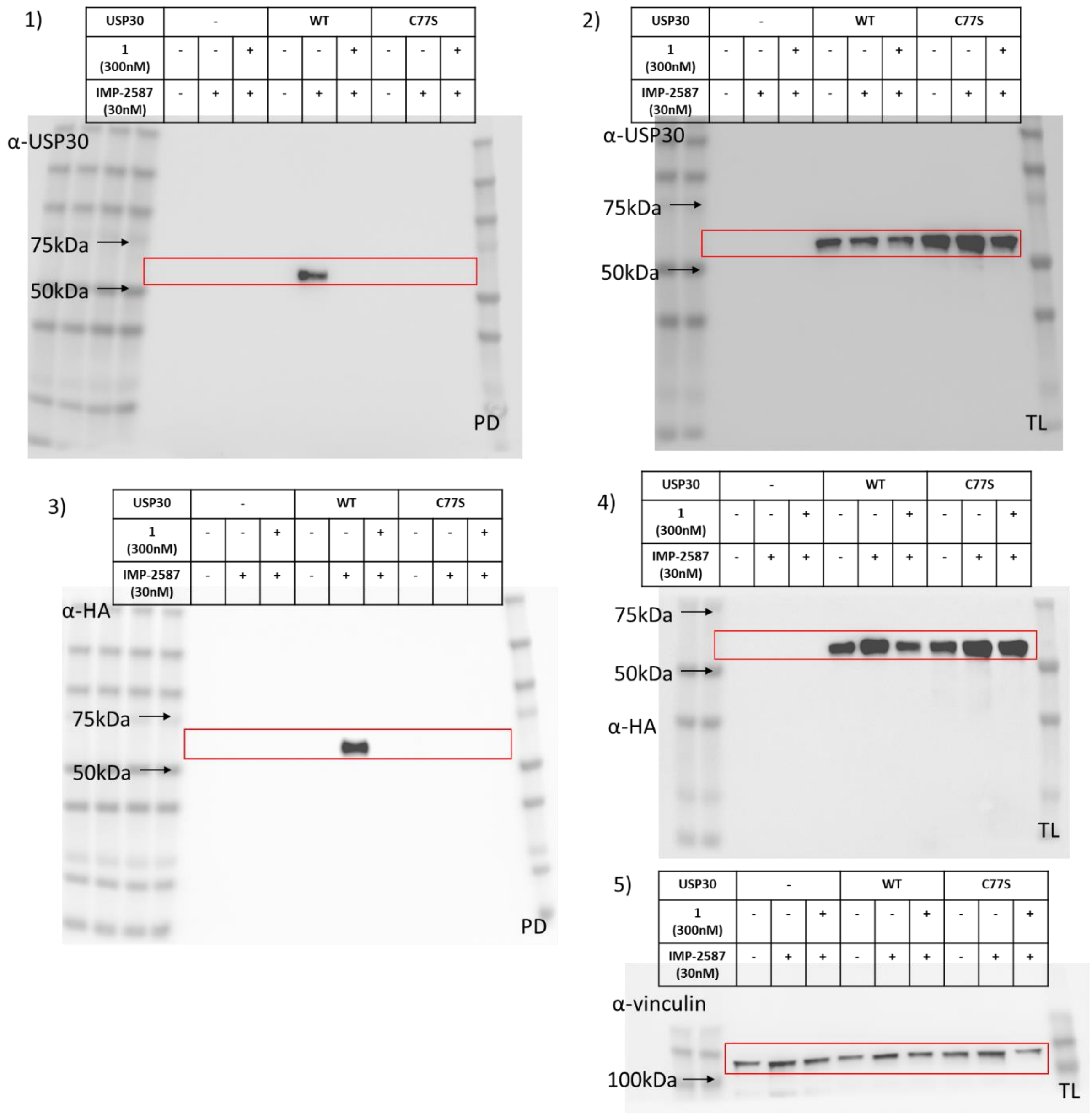

Figure S4. Overlayed uncropped blots (marker and blots) for HA-tagged WT or CS USP30 overexpressed experiments in Fig. 4b. TL –Total Lysate; PD –Pull-down. Fig. 4b displayed the bands in red box.

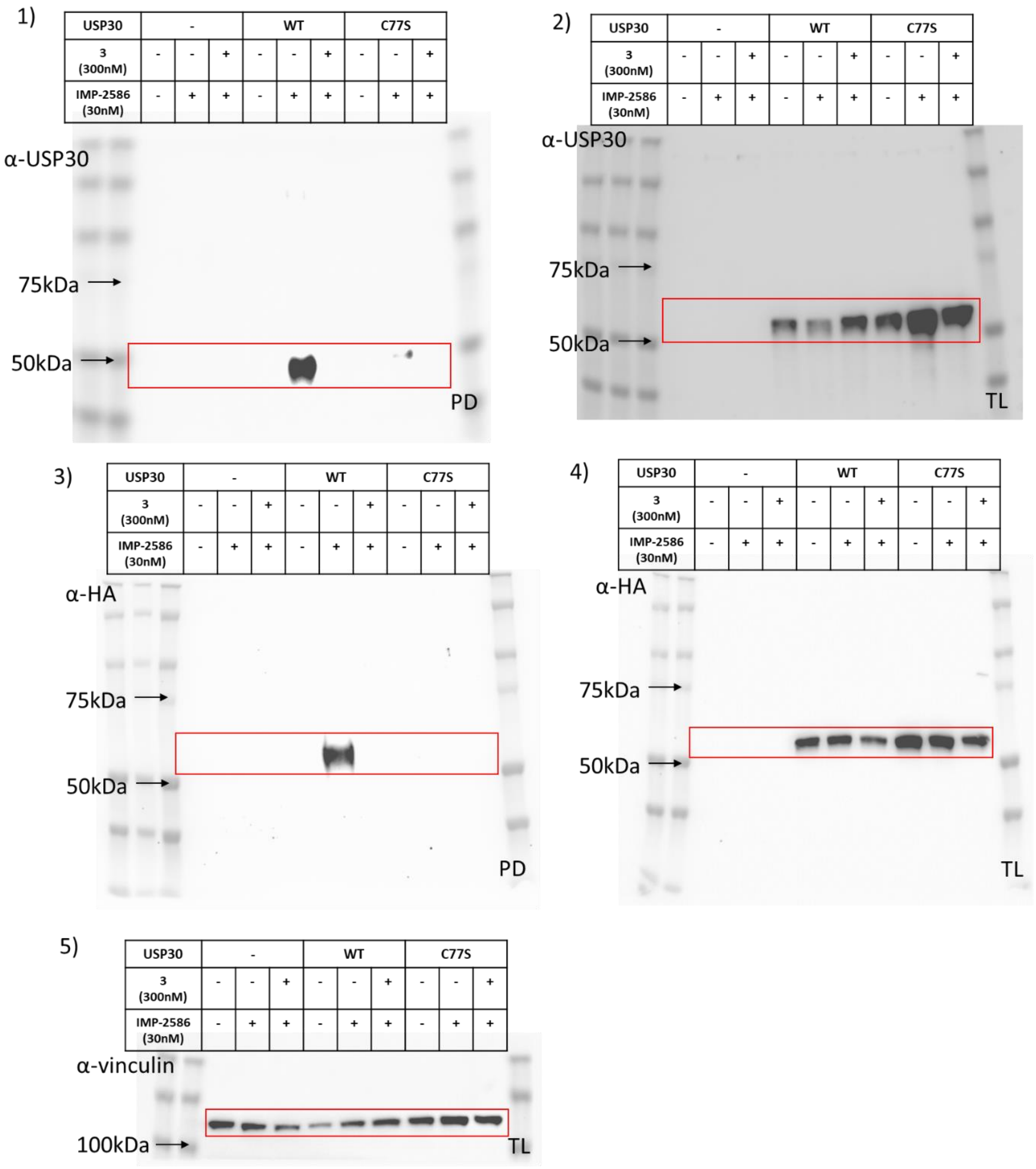

Figure S5. Overlayed uncropped blots (marker and blots) for HA-tagged WT or CS USP30 overexpressed experiments in Fig. 4c. TL –Total Lysate; PD –Pull-down. Fig. 4c displayed the bands in red box.

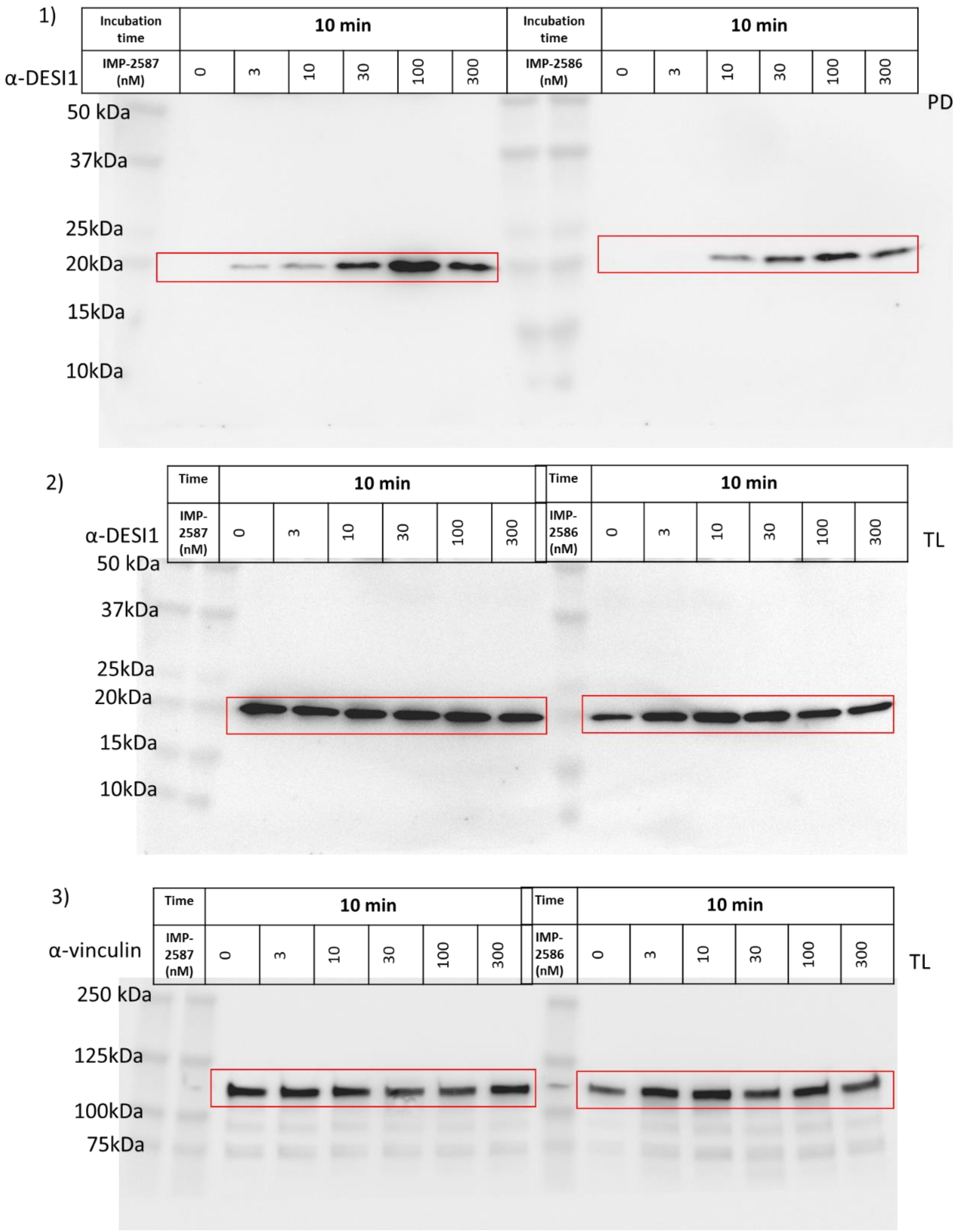

Figure S6. Overlayed uncropped blots (marker and blots) for in-cell DESI1 engagement experiments in Fig. 5e and 5f. TL –Total Lysate; PD –Pull-down. Fig. 5e and 5f displayed the bands in red box.

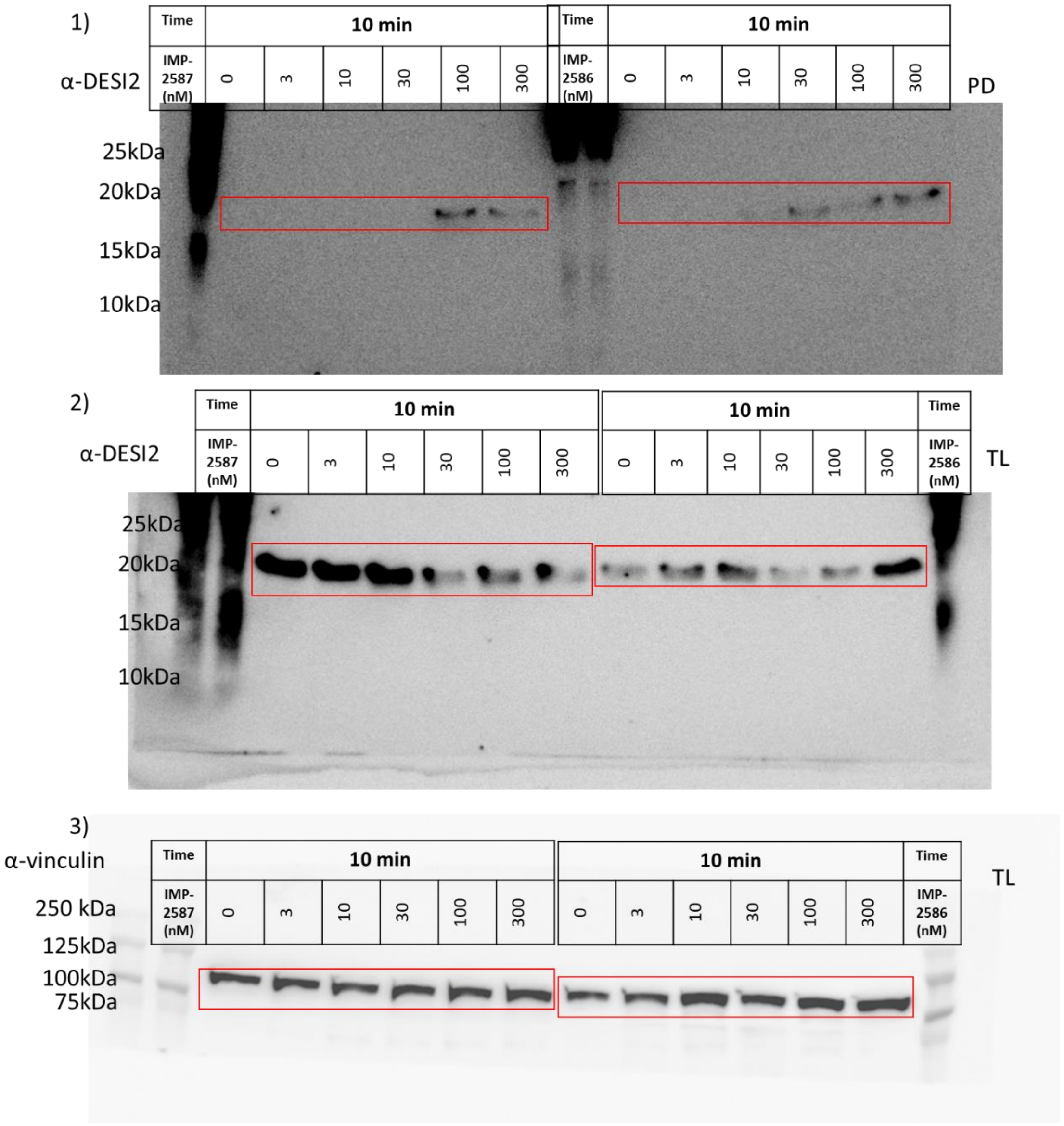

Figure S7. Overlayed uncropped blots (marker and blots) for in-cell DESI2 engagement experiments in Fig.5g and Fig 5h. TL –Total Lysate; PD –Pull-down. Fig. 5g and 5h displayed the bands in red box.

2. Supplementary tables

Table S1. Compound validation using FP assay against USP30 & UCHL1

origin	Compound ID	Structure	IC_{50} against USP30 (nM)	IC_{50} against UCHL1 (nM)
Mission ABP	**2**(**IMP-2587**)	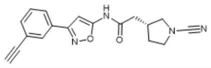	12.6	Not active
Forma ABP	**4**(I**MP-2586**)	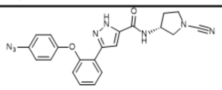	16.3	Not active
Forma inhibitor	**3**	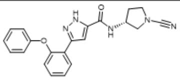	4.8	-
UCHL1<br>1st generation probe	**MT16-205**[3]	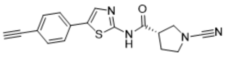	Not active	**-**
UCHL1<br>1st generation inhibitor	**MT16-001**[3]	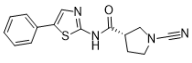	Not active	**-**

Table S2. List of enriched proteins by LC-MS/MS analysis of ABP IMP-2587 labelling (Fig.5a)

Gene names	Protein names	-Log P value	Difference
ABHD6	Monoacylglycerol lipase ABHD6	3.03	2.06
ALDH1A1	Retinal dehydrogenase 1	3.25	2.95
ALDH1A2	Retinal dehydrogenase 2	4.68	3.97
ALDH1B1	Aldehyde dehydrogenase X, mitochondrial	4.29	3.53
ALDH2	Aldehyde dehydrogenase, mitochondrial	4.68	3.49
ALDH3A2	Fatty aldehyde dehydrogenase	4.29	2.95
ALDH9A1	4-trimethylaminobutyraldehyde dehydrogenase	2.86	1.70
C21orf33		1.77	0.92
CTSZ	Cathepsin Z	4.39	2.29
DESI1	Desumoylating isopeptidase 1	4.70	3.61
DESI2	Desumoylating isopeptidase 2	3.14	2.02
FAAH	Fatty-acid amide hydrolase 1	3.88	3.97
FAAH2	Fatty-acid amide hydrolase 2	2.99	4.10
GET4	Golgi to ER traffic protein 4 homolog	1.59	0.76
H2AFV;H2AFZ	Histone H2A;Histone H2A.V;Histone H2A.Z	1.35	0.65
HMGCS1	Hydroxymethylglutaryl-CoA synthase, cytoplasmic	1.54	0.75
ISOC1	Isochorismatase domain-containing protein 1	5.45	4.06
ISOC2	Isochorismatase domain-containing protein 2, mitochondrial	3.70	3.87
MRPS2	28S ribosomal protein S2, mitochondrial	3.22	2.58
NIT1	Nitrilase homolog 1	3.67	2.01
NIT2	Omega-amidase NIT2	1.25	0.61
NPM3	Nucleoplasmin-3	1.39	0.89
PARK7	Protein deglycase DJ-1	2.75	1.32
RPS8	40S ribosomal protein S8	2.81	-0.44
RTN1	Reticulon;Reticulon-1	3.75	1.77
RTN3	Reticulon-3	1.94	1.07
UCHL1	Ubiquitin carboxyl-terminal hydrolase;Ubiquitin carboxyl-terminal hydrolase isozyme L1	2.41	0.76

USP30	Ubiquitin carboxyl-terminal hydrolase 30;Ubiquitin carboxyl-terminal hydrolase	3.22	2.05

Table S3. List of enriched proteins by LC-MS/MS analysis of compound 1 out-competition with IMP-2587 labelling (Fig.5b)

Gene names	Protein names	-Log P value	Difference
ABHD6	Monoacylglycerol lipase ABHD6	2.87	-1.95
ACTG1	Actin, cytoplasmic 2;Actin, cytoplasmic 2, N-terminally processed	1.72	-0.57
ALDH1A1	Retinal dehydrogenase 1	3.22	-2.59
ALDH1A2	Retinal dehydrogenase 2	4.60	-3.93
ALDH1B1	Aldehyde dehydrogenase X, mitochondrial	4.01	-3.81
ALDH2	Aldehyde dehydrogenase, mitochondrial	4.33	-3.07
ALDH3A2	Fatty aldehyde dehydrogenase	3.19	-1.88
ALDH9A1	4-trimethylaminobutyraldehyde dehydrogenase	2.77	-1.60
CTSZ	Cathepsin Z	4.70	-2.01
DCD	Dermcidin;Survival-promoting peptide;DCD-1	2.78	-0.34
DESI1	Desumoylating isopeptidase 1	4.57	-3.45
DESI2	Desumoylating isopeptidase 2	2.78	-1.75
FAAH	Fatty-acid amide hydrolase 1	3.99	-3.46
FAAH2	Fatty-acid amide hydrolase 2	3.54	-3.82
IMPDH2	Inosine-5-monophosphate dehydrogenase 2	1.35	-0.70
ISOC1	Isochorismatase domain-containing protein 1	4.51	-3.50
ISOC2	Isochorismatase domain-containing protein 2, mitochondrial	3.82	-3.53
MRPS2	28S ribosomal protein S2, mitochondrial	3.80	-2.58
NIT1	Nitrilase homolog 1	3.61	-1.86
RPL10	60S ribosomal protein L10	1.43	-0.52
RPL34	60S ribosomal protein L34	1.21	-0.67
RTN1	Reticulon;Reticulon-1	2.71	-0.69
RTN3	Reticulon-3	1.58	-0.64
USP30	Ubiquitin carboxyl-terminal hydrolase 30;Ubiquitin carboxyl-terminal hydrolase	2.83	-1.82

Table S4. List of enriched proteins by LC-MS/MS analysis of ABP IMP-2586 labelling (Fig.5c)

Gene names	Protein names	-Log P value	Difference
ALDH1A2	Retinal dehydrogenase 2	5.51	3.54
ALDH1B1	Aldehyde dehydrogenase X, mitochondrial	4.32	2.25
ALDH2	Aldehyde dehydrogenase, mitochondrial	4.03	2.13
ALYREF	THO complex subunit 4	2.45	-0.42
ASAH1	Acid ceramidase;Acid ceramidase subunit alpha;Acid ceramidase subunit beta	1.95	1.08
AURKAIP1	Aurora kinase A-interacting protein	1.31	0.81
C21orf33		5.03	3.93
CAV1	Caveolin	1.02	-2.47
CAV1	Caveolin-1	1.46	-1.61
CFAP20	Cilia- and flagella-associated protein 20	1.66	-0.41
CNP	2,3-cyclic-nucleotide 3-phosphodiesterase	1.24	-0.68
DESI1	Desumoylating isopeptidase 1	3.75	3.04
DESI2	Desumoylating isopeptidase 2	4.83	3.40
HSDL1	Inactive hydroxysteroid dehydrogenase-like protein 1	1.07	1.22
ISOC1	Isochorismatase domain-containing protein 1	3.02	1.71
ISOC2	Isochorismatase domain-containing protein 2, mitochondrial	4.07	3.62
MALT1	Mucosa-associated lymphoid tissue lymphoma translocation protein 1	1.36	0.75
MDC1	Mediator of DNA damage checkpoint protein 1	1.75	0.38
PARK7	Protein deglycase DJ-1	2.89	1.30
PGAP1	GPI inositol-deacylase	5.45	2.66
PPAT	Amidophosphoribosyltransferase	3.10	0.83
RER1	Protein RER1	1.18	-1.34
RPL23A	60S ribosomal protein L23a	1.54	-0.57
RPL27	60S ribosomal protein L27	1.33	-0.76
RTN1	Reticulon;Reticulon-1	2.75	1.53
RTN3	Reticulon-3	2.76	0.91

SFT2D3	Vesicle transport protein SFT2C	1.25	-1.51
SMARCA4	Transcription activator BRG1	1.65	0.52
SRP68	Signal recognition particle subunit SRP68	1.84	0.45
TMEM109	Transmembrane protein 109	0.99	-1.56
TMEM263	Transmembrane protein 263	1.69	-0.44
USP30	Ubiquitin carboxyl-terminal hydrolase 30;Ubiquitin carboxyl-terminal hydrolase	3.04	4.80

Table S5. List of enriched proteins by LC-MS/MS analysis of compound 3 out-competition with IMP-2586 labelling (Fig.5d)

Gene names	Protein names	-Log P value	Difference
ALDH1A2	Retinal dehydrogenase 2	4.26	-2.44
ALDH1B1	Aldehyde dehydrogenase X, mitochondrial	4.65	-1.78
ALDH2	Aldehyde dehydrogenase, mitochondrial	3.90	-1.64
C21orf33		5.97	-3.65
CLPTM1	Cleft lip and palate transmembrane protein 1	1.72	-1.18
DESI1	Desumoylating isopeptidase 1	4.73	-2.81
DESI2	Desumoylating isopeptidase 2	4.91	-3.13
ISOC1	Isochorismatase domain-containing protein 1	3.39	-1.80
ISOC2	Isochorismatase domain-containing protein 2, mitochondrial	4.08	-3.08
LUC7L3	Luc7-like protein 3	1.64	-0.51
NUB1	NEDD8 ultimate buster 1	2.04	-0.38
PGAP1	GPI inositol-deacylase	5.14	-2.38
PPAT	Amidophosphoribosyltransferase	1.96	-0.44
RTN3	Reticulon-3	2.02	-0.42
SRP68	Signal recognition particle subunit SRP68	1.80	-0.40
USP30	Ubiquitin carboxyl-terminal hydrolase 30;Ubiquitin carboxyl-terminal hydrolase	3.16	-4.18

3. General synthetic methods

All chemicals and solvents were obtained from Sigma-Aldrich UK or VWR international Ltd and used without further purification. All anhydrous conditions were performed in oven-dried glassware under an argon or nitrogen atmosphere. Dried solvents were dispensed using Pure SolvTM solvent drying towers (Innovative technology Inc.). For purifications, HPLC-grade solvents (≥ 99% purity) were used as purchased from Sigma-Aldrich Chemical Co. Ltd. or Fisher Scientific UK.

Analytical techniques Thin layer chromatography (TLC) analysis was performed on Merck silica gel 60 F254 aluminium plates for monitoring reaction progresses. Spots were visualized under UV lamp (254 nm) and/or stained with potassium permanganate for UV-inactive compounds, or 2,4-dinitrophenylhydrazine (DNP) for carbonyl compounds. Flash column chromatography was manually performed on Merck silica gel 60 Å, eluting with solvents as stated, under positive air pressure. ^{1}H and ^{13}C NMR spectra were recorded on a 400 MHz Bruker AV NMR spectrometer at 298 K (400 MHz for ^{1}H and 101 MHz for ^{13}C NMR) in chloroform-d ($CDCl_3$, Acros Organics) as internal reference (δH = 7.26 ppm and δC = 77.16 ppm). Chemical shifts (δ) are reported in part per million (ppm) relative to tetramethylsilane (TMS) as reference where δH and δC (TMS) = 0.00 ppm and their assignments are shown as multiplicity (s, singlet; d, doublet; dd, doublet of doublets; t, triplet; q, quartet; m, multiplet; br., broad), coupling constant, and number of protons. The coupling constants (J values), quoted in hertz (Hz) and recorded to the nearest 0.1 Hz, are calculated by MestReNova© NMR software.

1) Chemical synthesis of the probes

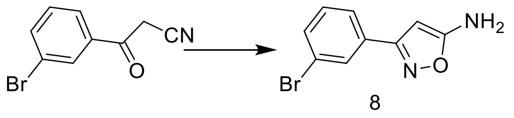

3-(3-Bromophenyl)isoxazol-5-amine(8): To a solution of 3-(4-bromophenyl)-3-oxopropanenitrile (CAS Number 70591-86-5; 2 g, 8.9 mmol) and NH_2OH.HCl (0.742 g, 10.70 mmol) in water (23 ml) was added NaOH (0.712 g, 17.8 mmol) portion wise at 0°C. The reaction mixture was heated to 100°C for 3 h. The resulting reaction mixture was cooled to rt and poured into water (125 ml) then extracted with EtOAc (4 x 30 ml). The combined organic layer was dried over Na_2SO, filtered and concentrated under reduced pressure. The resulting residue was purified by flash column chromatography (32%, EtOAc in hexane) yielding 860 mg 3-(4-bromophenyl)isoxazol-5-amine with 41% yield. ^{1}H NMR (400 MHz, DMSO) δ 7.90 (t, *J* = 1.8 Hz, 1H), 7.74 (ddd, *J* = 7.7, 1.6, 1.0 Hz, 1H), 7.63 (ddd, *J* = 8.0, 2.1, 1.0 Hz, 1H), 7.41 (t, *J* = 7.9 Hz, 1H), 6.86 (s, 2H), 5.47 (s, 1H). ^{13}C NMR (101 MHz, DMSO) δ 171.8, 161.7, 132.7, 131.5, 129.1, 125.7, 122.5, 75.6.

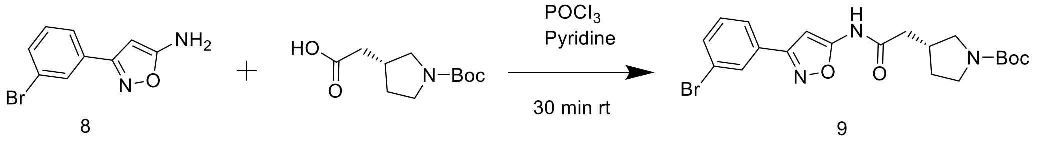

***tert*-Butyl (R)-3-(2-((3-(3-bromophenyl)isoxazol-5-yl)amino)-2-oxoethyl)pyrrolidine-1-carboxylate (9):** To a solution of 3-(3-bromophenyl)isoxazol-5-amine (500 mg , 2.1 mmol) and (R)-2-(1-(*tert*-butoxycarbonyl)pyrrolidin-3-yl)acetic acid (CAS Number: 204688-60-8; 480 mg , 2.1 mmol) in pyridine (15ml) was added POCI3 (0.6 ml, 6.27 mmol) dropwise at 0°C. The reaction mixture was stirred for 30

min at rt. The resulting reaction mixture was poured into water (150 ml) and extracted with EtOAc (3 x 25 ml). The combined organic layer was dried over Na_2SO_4, filtered and concentrated under reduced pressure. The resulting residue was purified by flash column chromatography (36%, EtOAc in hexane) yielding tert-butyl (R)-3-(2-((3-(3-bromophenyl)isoxazol-5 yl)amino)-2-oxoethyl)pyrrolidine-1-carboxylate (407 mg) with 43% yield. ^{1}H NMR (400 MHz, DMSO) δ 11.76 (s, 1H), 8.05 (t, *J* = 1.8 Hz, 1H), 7.88 (dt, *J* = 7.8, 1.2 Hz, 1H), 7.70 (ddd, *J* = 8.1, 2.1, 1.0 Hz, 1H), 7.47 (t, *J* = 7.9 Hz, 1H), 3.49 (d, *J* = 6.0 Hz, 1H), 3.36 (d, *J* = 3.8 Hz, 1H), 3.20 (q, *J* = 8.8 Hz, 1H), 2.91 (t, *J* = 7.9 Hz, 1H), 2.53 (d, *J* = 3.3 Hz, 3H), 1.99 (s, 1H), 1.63 – 1.45 (m, 1H), 1.40 (s, 9H). ^{13}C NMR (101 MHz, DMSO) δ 169.3, 161.9, 153.9, 149.6, 133.4, 131.7, 131.5, 129.4, 126.0, 122.8, 86.6, 78.7, 51.2, 45.3, 39.0, 35.4, 30.5, 28.7.

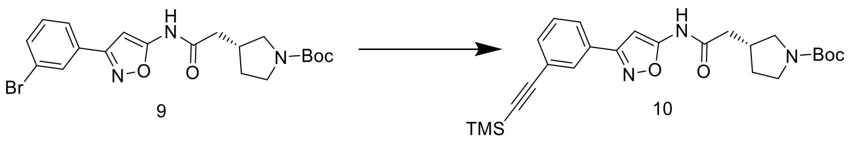

***tert*-Butyl*(R)*-3-(2-oxo-2-((3-(3-((trimethylsilyl)ethynyl)phenyl)isoxazol-5-yl)amino)ethyl)pyrrolidine-1-carboxylate (10):** To a solution of compound **9** (150 mg, 0.334 mmol, 1.0 equiv.) in degassed, dry DMF (0.3 mL) was added dichlorobis(triphenylphosphine)palladium (7 mg, 0.01 mmol, 0.03 equiv.), copper iodide (1.33 mg, 0.007 mmol, 0.02 equiv.), triethylamine (233 µL, 1.67 mmol, 5.0 equiv.) and ethynyl(trimethyl)silane (55 µL, 0.4 mmol, 1.2 equiv.). The reaction mixture was heated at 65 °C for 2 h then allowed cool to room temperature and quenched by addition of water (4 mL). The aqueous mixture was extracted with EtOAc (3 × 15 mL) and combined organic extracts were washed with brine, dried over Na_2SO_4 and concentrated under reduced pressure. The crude material was purified by automated flash chromatography (Biotage SNAP KP-Sil 100 g cartridge, 5-40% EtOAc in *n*-Hexane) to obtain compound **10** (67 mg) with 45% yield. ^{1}H NMR (400 MHz, $CDCl_3$) δ 10.57 (d, *J* = 28.6 Hz, 1H), 7.92 – 7.86 (m, 1H), 7.76 (t, *J* = 9.4 Hz, 1H), 7.52 (d, *J* = 7.8 Hz, 1H), 7.38 (td, *J* = 7.6, 4.5 Hz, 1H), 6.74 (d, *J* = 18.9 Hz, 1H), 3.63 (ddd, *J* = 26.2, 10.8, 6.8 Hz, 1H), 3.52 – 3.28 (m, 2H), 3.09 (dd, *J* = 10.9, 6.6 Hz, 1H), 2.83 – 2.56 (m, 3H), 2.13 (ddd, *J* = 25.8, 13.7, 7.1 Hz, 1H), 1.65 (dt, *J* = 19.8, 7.9 Hz, 1H), 1.47 (s, 9H), 0.25 (s, 9H). ^{13}C NMR (101 MHz, $CDCl_3$) δ 168.6, 163.1, 161.5, 155.3, 133.4, 130.5, 129.2, 128.9, 126.7, 124.0, 104.2, 95.3, 87.2, 79.9, 51.3, 45.6, 44.9, 39.4, 35.2, 34.8, 31.5, 30.6, 28.6.

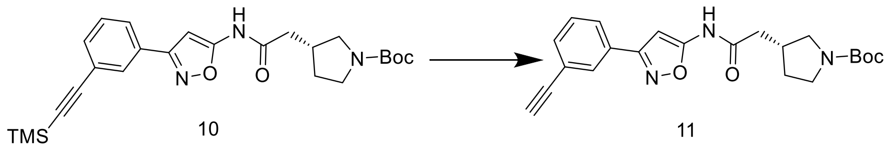

***tert*-Butyl*(R)*-3-(2-((3-(3-ethynylphenyl)isoxazol-5-yl)amino)-2-oxoethyl)pyrrolidine-1-carboxylate (11):** To a solution of Compound **10** (67 mg, 0.143 mmol) in MeOH: THF (1:1, 3 ml) were added K_2CO_3 (40 mg, 0.286 mmol). The mixture was stirred at rt for 2 h. The resulting reaction solvent was evaporated and compound **11** was purified by isolera (52%, reverse phase C18, 12g, 5-95% acetonitrile) to obtain the desired compound (17 mg) with a yield of 30%. ^{1}H NMR (400 MHz, DMSO) δ 11.76 (s, 1H), 8.05 (t, *J* = 1.8 Hz, 1H), 7.88 (dt, *J* = 7.9, 1.3 Hz, 1H), 7.70 (ddd, *J* = 8.1, 2.1, 1.0 Hz, 1H), 7.47 (t, *J* = 7.9 Hz, 1H), 6.83 (s, 1H), 3.49 (d, *J* = 6.1 Hz, 1H), 3.43 – 3.27 (m, 12H), 3.21 (q, *J* = 9.0 Hz, 1H), 3.02 – 2.86 (m, 1H), 1.99 (s, 1H), 1.56 (s, 1H), 1.40 (s, 8H). ^{13}C NMR (101 MHz, DMSO) δ 169.2, 162.8, 161.9, 153.9, 149.9, 133.4, 131.7, 131.6, 129.4, 126.0, 122.8, 86.6, 51.2, 45.3, 35.4, 30.5, 28.7.

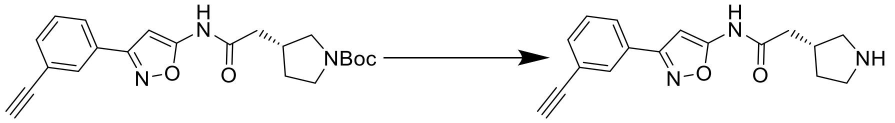

11 12

***(R)*-N-(3-(3-ethynylphenyl)isoxazol-5-yl)-2-(pyrrolidin-3-yl)acetamide (12):** A suspension of **compound 11** (35 mg, 0.088 mmol, 1 equiv.) and K_2CO_3 (122 mg, 0.88 mmol, 10 equiv.) in degassed, dry DCM (2.0 mL) was cooled to 0°C. TMSI (25.0 µL, 0.176 mmol, 2.0 equiv.) was slowly supplemented to the reaction mixture using a micro syringe. The solution was then stirred at room temperature for 30 min. The mixture was then added to a saturated solution of $NaHCO_3$ (5 mL) and extracted with DCM (3 × 5 mL). The combined organic layers were washed with brine (5 mL), dried over Na_2SO_4, filtered and concentrated under reduced pressure. The crude residue was purified by flash column chromatography (10% 2 M methanolic ammonia in DCM) to yield compound **12** and proceed for the following reaction without further purification.

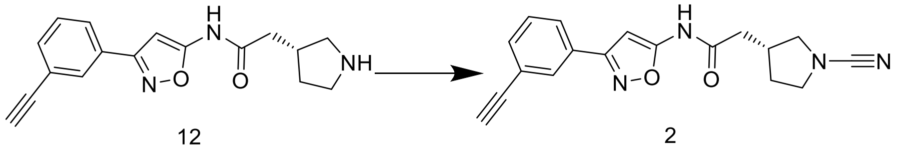

12 2

***(R)*-2-(1-Cyanopyrrolidin-3-yl)-N-(3-(3-ethynylphenyl)isoxazol-5-yl)acetamide (2, IMP-2587):** To a stirred solution of compound **12** (20 mg, 0.067 mmol, 1.0 equiv.) in THF (1 mL) was added K_2CO_3 (38 mg, 0.27 mmol, 4.0 equiv.). The reaction mixture was cooled to -20°C and a solution of BrCN in DCM (3 M, 27 µL, 0.059 mmol, 1.2 equiv.) was added dropwisely. The reaction mixture was allowed to warm to room temperature and stirred for 1 h. The resulting mixture was added to water (5 mL), supplemented with K_2CO_3 (35 mg) and extracted with DCM (3 × 5 mL). The combined organic layers were dried over $MgSO_4$, filtered and concentrated under reduced pressure. The crude material was purified by automated flash column chromatography (57%, SNAP KP-Sil 10 g, 1 to 6% MeOH in DCM, 36 mL/min) to yield compound **2** (12 mg) with a yield of 44% for two steps. ^{1}H NMR (400 MHz, $CDCl_3$) δ 10.03 (s, 1H), 7.92 (t, *J* = 1.7 Hz, 1H), 7.79 (dt, *J* = 7.9, 1.4 Hz, 1H), 7.57 (dt, *J* = 7.7, 1.4 Hz, 1H), 7.43 (t, *J* = 7.8 Hz, 1H), 6.72 (s, 1H), 3.69 (dd, *J* = 9.5, 6.8 Hz, 1H), 3.60 – 3.43 (m, 2H), 3.19 (dd, *J* = 9.6, 6.5 Hz, 1H), 2.90 – 2.69 (m, 1H), 2.59 (d, *J* = 7.2 Hz, 2H), 2.18 (q, *J* = 6.1 Hz, 1H), 1.73 (dt, *J* = 12.7, 7.4 Hz, 1H). ^{13}C NMR (101 MHz, $CDCl_3$) δ 167.6, 163.0, 161.1, 133.7, 130.4, 129.2, 129.1, 127.0, 123.0, 118.0, 87.3, 82.7, 78.3, 55.3, 49.7, 38.8, 35.4, 31.2. HR-MS calculated for $C_{18}H_{15}N_4O_2$[M-H]$^-$= 319.1190, found 319.1201.

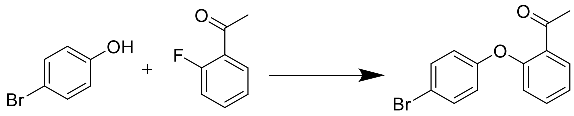

13

1-(2-(4-Bromophenoxy)phenyl)ethan-1-one (13): 4-Bromophenol (17.34 mmol, 1.1 equiv) and anhydrous K_2CO_3 (17.34 mmol, 1.1 equiv) were added to a solution of 2-fluoroacetophenone (15.763 mmol, 1.0 equiv) in DMA (20 mL) and the resulting mixture was slowly heated to 170 °C. After stirring for 4 h and cooling to room temperature, the reaction mixture was poured into H_2O (100 mL) and the aqueous layer was extracted with DCM (3×40 mL). The desired product was obtained after drying over MgSO4, filtration and concentration in vacuo for 4.2 g with 92% yield. ^{1}H NMR (400 MHz, $CDCl_3$) δ 7.84 (dd, *J* = 7.8, 1.8 Hz, 1H), 7.50 – 7.41 (m, 3H), 7.19 (td, *J* = 7.5, 1.1 Hz, 1H), 6.93 – 6.85 (m, 3H), 2.60 (s,

3H). ^{13}C NMR (101 MHz, $CDCl_3$) δ 198.6, 155.9, 155.7, 133.8, 133.1, 130.7, 124.1, 120.4, 119.6, 116.4, 31.5.

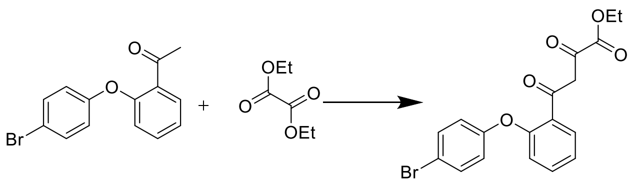

13 14

Ethyl 4-(2-(4-bromophenoxy)phenyl)-2,4-dioxobutanoate (14): 8.57 mmol of compound **13** and 1.283 ml (9.45 mmol) of diethyl oxalate were dissolved in 4 ml of anhydrous THF. This solution was added dropwise to the stirred suspension of 310 mg (12.88 mmol) of sodium hydride in 7 ml of anhydrous THF. Heating and evolution of H_2 occurred, and sodium salt of diketoester began to precipitate. When addition was completed, heating and stirring of reaction mixture with reflux condenser were continued for 10–30 min, until evolution of gas in bubble counter disappeared. Then THF was evaporated under reduced pressure. 20 ml of ice water, 10 ml of CH_2Cl_2 and 0.8 ml of H_2SO_4 were added to the residue of sodium salt of diketoester. Mixture was shacked until dissolution of solid and transferred to separation funnel. Organic layer was evaporated under reduced pressure to obtain compound **14** (2.8 g) in 82% yield. ^{1}H NMR (400 MHz, $CDCl_3$) δ 14.94 (s, 1H), 7.88 (dd, *J* = 7.9, 1.8 Hz, 1H), 7.47 – 7.35 (m, 3H), 7.22 – 7.14 (m, 2H), 6.89 (dd, *J* = 8.3, 1.1 Hz, 1H), 6.86 – 6.82 (m, 2H), 4.24 (q, *J* = 7.1 Hz, 2H), 1.26 (t, *J* = 7.1 Hz, 3H). ^{13}C NMR (101 MHz, $CDCl_3$) δ 189.6, 169.2, 161.9, 155.6, 155.6, 134.5, 133.0, 130.8, 127.5, 124.3, 120.3, 119.9, 116.5, 102.8, 62.4, 14.0.

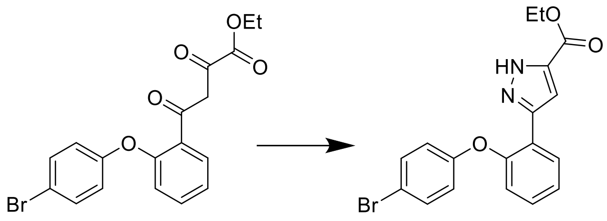

14 15

Ethyl 3-(2-(4-bromophenoxy)phenyl)-1H-pyrazole-5-carboxylate (15): Hydrazine dihydrochloride ($N_2H_4{\cdot}2HCl$) (967 mg, 9.21 mmol) dissolved in ethanol was added to compound **14** (2.4 g, 6.14 mmol) and refluxed for 4 hours. After the completion of the reaction, ethanol was evaporated under high vacuum conditions. 15-20 mL water was mixed to the above reaction residue, followed by extraction with 4x10 mL of ethyl acetate. The four fractions of organic layer were mixed with each other and dried on anhydrous Na_2SO_4. The ethyl acetate, organic solvent was evaporated by rotavapor under reduced pressure to give crude compound. A silica gel column chromatography technique with 7:3 v/v hexane and ethyl acetate solvent system were applied to the above crude to afford compound **15** (1.8 g, 72% yield). ^{1}H NMR (400 MHz, DMSO) δ 13.96 (s, 1H), 7.95 (s, 1H), 7.59 – 7.49 (m, 2H), 7.46 – 7.37 (m, 1H), 7.31 (td, *J* = 7.5, 1.2 Hz, 1H), 7.08 – 7.00 (m, 2H), 7.00 – 6.90 (m, 2H), 4.27 (q, *J* = 7.1 Hz, 2H), 1.27 (t, *J* = 7.1 Hz, 3H). ^{13}C NMR (101 MHz, DMSO) δ 156.5, 152.8, 133.3, 130.6, 129.1, 125.2, 120.6, 115.5, 108.5, 60.9, 55.4, 14.7.

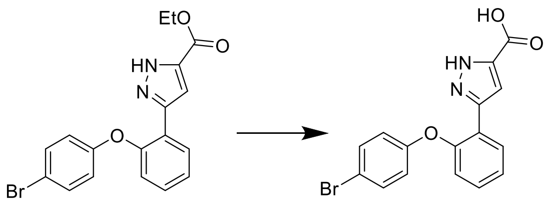

15 16

3-(2-(4-Bromophenoxy)phenyl)-1H-pyrazole-5-carboxylic acid (16): 5 mL of 2M NaOH was mixed with a solution of compound **15** (2 g, 5.16 mmol) in MeOH (6 ml) and the reaction mass was stirred for 12 h at room temperature. After evaporation of most of the methanol, the aqueous phase was adjusted to pH 7 and extracted with 4x10 mL of ethyl acetate. The four fractions of organic layer were layers mixed with each other and dried on anhydrous Na_2SO_4. The ethyl acetate organic solvent was evaporated by rotavapor under reduced pressure to give crude compound. Silica gel column chromatography technique with 7:3 v/v hexane and ethyl acetate solvent system were applied to afford compound **16** (1.3 g, 68% yield). ^{1}H NMR (400 MHz, DMSO) δ 8.09 – 7.95 (m, 1H), 7.59 – 7.47 (m, 2H), 7.44 – 7.35 (m, 1H), 7.30 (td, *J* = 7.5, 1.3 Hz, 1H), 7.04 (dd, *J* = 8.1, 1.3 Hz, 1H), 6.98 – 6.86 (m, 3H). ^{13}C NMR (101 MHz, DMSO) δ 156.8, 152.5, 133.3, 130.2, 129.0, 125.3, 121.2, 120.1, 115.1, 108.3.

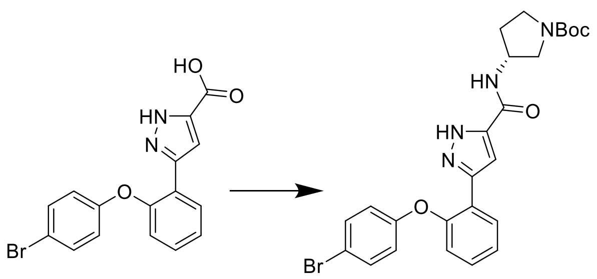

16 **17**

***tert*-Butyl(R)-3-(3-(2-(4-bromophenoxy)phenyl)-1H-pyrazole-5-carboxamido)pyrrolidine-1-carboxylate (17):** Compound **16** (450 mg, 1.242 mmol), tert-butyl (R)-3-aminopyrrolidine-1-carboxylate (252 ul, 1.476 mmol), HATU (576 mg, 1.521 mmol) was dissolved in DMF (5 mL), following by DIPEA (450 ul, 2.574 mmol) addition. The resulting mixture was stirred at room temperature for overnight. Water was added to the reaction mixture. The resulting mixture was extracted with ethylacetate 3 times. The ethyl acetate organic solvent was dried on anhydrous Na_2SO_4 and evaporated by rotavapor under reduced pressure to give crude compound. Silica gel column chromatography was applied to afford 459 mg compound **17** with a yield of 70%. ^{1}H NMR (400 MHz, MeOD) δ 7.75 (d, *J* = 7.3 Hz, 1H), 7.41 – 7.33 (m, 2H), 7.32 – 7.24 (m, 1H), 7.23 – 7.10 (m, 2H), 6.94 – 6.74 (m, 3H), 4.51 (p, *J* = 5.9 Hz, 1H), 3.65 (dd, *J* = 11.2, 6.6 Hz, 1H), 3.52 – 3.18 (m, 3H), 2.14 (q, *J* = 6.9 Hz, 1H), 1.96 (q, *J* = 6.2 Hz, 1H), 1.42 (s, 9H). ^{13}C NMR (101 MHz, MeOD) δ 155.8, 154.9, 153.2, 132.6, 130.0, 128.6, 124.4, 120.3, 119.4, 115.8, 105.3, 79.7, 50.8, 50.4, 49.3, 48.7, 44.2, 43.7, 37.7, 30.8, 30.0, 27.6.

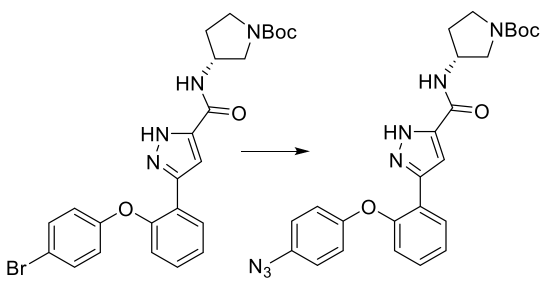

17 **19**

tert-Butyl(R)-3-(3-(2-(4-azidophenoxy)phenyl)-1H-pyrazole-5-carboxamido)pyrrolidine-1-carboxylate (19): Compound **17** (100 mg, 0.2 mmol), NaN_3 (24.7mg, 0.4 mmol), sodium ascorbate (1.9 mg, 0.001 mmol), CuI (3.62 mg, 0.02 mmol), N,N-dimethylethyldiamine (3 µL, 0.03 mmol) were dissolved in EtOH–H_2O (7:3) in a two-necked round-bottom flask equipped with a stirring bar and a reflux condenser. After the reaction mixture was degassed, and then introduced under an argon atmosphere, the reaction mixture was stirred under reflux and the progress of the reaction was followed by TLC. When the starting material was completely consumed, or when the progress of the reaction had stopped, the reaction mixture was allowed to cool down to r.t., and the crude mixture was purified by flash chromatography, giving the desired compound **19** (49mg) with a yield of 50%. ^1H NMR (400 MHz, $CDCl_3$) δ 10.04 (s, 1H), 7.76 (dd, *J* = 7.8, 1.7 Hz, 1H), 7.32 – 7.13 (m, 4H), 7.10 – 6.94 (m, 4H), 6.85 (dd, *J* = 8.3, 1.2 Hz, 1H), 4.63 (h, *J* = 5.9 Hz, 1H), 3.70 (dd, *J* = 11.4, 6.4 Hz, 1H), 3.47 (m, 2H), 3.37 – 3.20 (m, 1H), 2.24-2.15 (m, 1H), 1.86-2.03 (m, 1H), 1.46 (s, 9H). ^{13}C NMR (101 MHz, $CDCl_3$) δ 162.1, 154.7, 154.2, 152.5, 146.5, 141.7, 136.4, 123.0, 128.6, 124.1, 121.1, 120.5, 119.3, 118.2, 104.7, 79.9, 51.7, 51.0, 49.2, 48.5, 44.3, 43.8, 31.9, 31.0, 29.7, 28.6, 28.5.

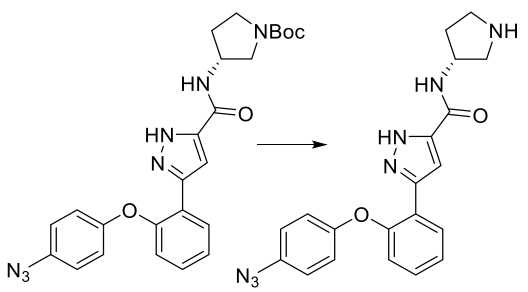

19 **21**

(R)-3-(2-(4-azidophenoxy)phenyl)-N-(pyrrolidin-3-yl)-1H-pyrazole-5-carboxamide(21): To a solution of compound **19** (87 mg, 0.177 mmol, 1.0 equiv.) in DCM (1.5 mL) was added TFA (0.4 mL) and the solution stirred at room temperature for 3 h. The reaction mixture was diluted with DCM (2 mL) and concentrated under reduced pressure to yield a crude oil that was triturated with Et_2O. The recovered solid was dried under high vacuum to yield compound **21** as an off-white solid which was used without further purification.

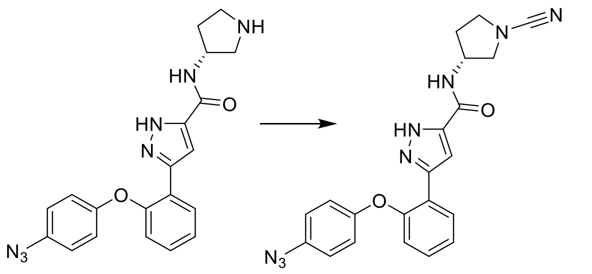

21 4

(R)-3-(2-(4-azidophenoxy)phenyl)-N-(1-cyanopyrrolidin-3-yl)-1H-pyrazole-5-carboxamide (4, IMP-2586): A solution of crude compound **21** and K_2CO_3 (80 mg, 0.570 mmol, 3.0 equiv.) was prepared in dry THF (2 mL) and cooled to -20°C. A solution of BrCN in DCM (3 M, 75 µL, 0.227 mmol, 1.2 equiv.) was added dropwise and the reaction mixture allowed to warm to room temperature and stirred for 1 h. The resulting mixture was added to water (5 mL) and extracted with EtOAc (3 × 5 mL). The combined organic phases were washed with brine (5 mL), dried over $MgSO_4$, filtered and concentrated under reduced pressure. The crude material was purified by automated flash chromatography to obtain the final compound **4** (22 mg) with a yield of 30%. ^{1}H NMR (400 MHz, $CDCl_3$) δ 11.43 (s, 1H), 7.77 (dd, *J* = 7.8, 1.8 Hz, 1H), 7.37 – 7.26 (m, 1H), 7.26 – 7.18 (m, 2H), 7.12 – 7.05 (m, 5H), 6.87 (dd, *J* = 8.2, 1.2 Hz, 1H), 4.90 – 4.61 (m, 1H), 3.76 (dd, *J* = 10.0, 6.1 Hz, 1H), 3.67 – 3.51 (m, 2H), 3.41 (ddd, *J* = 10.1, 4.3, 0.8 Hz, 1H), 2.30 (dddd, *J* = 13.3, 8.2, 7.2, 6.2 Hz, 1H), 2.04 (ddt, *J* = 12.9, 7.1, 5.3 Hz, 1H). ^{13}C NMR (101 MHz, $CDCl_3$) δ 161.9, 154.3, 152.2, 136.8, 130.1, 128.6, 124.1, 121.4, 120.7, 118.8, 118.0, 116.9, 104.1, 55.5, 49.1, 48.8, 31.7. HR-MS calculated for $C_{21}H_{19}N_8O_2$ $[M+H]^+$= 415.1631, found 415.1633.

2) Chemical synthesis of the parent inhibitors

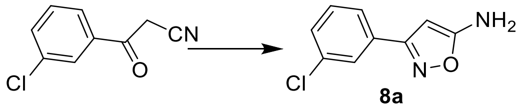

8a

3-(4-chlorophenyl)isoxazol-5-amine (8a): To a solution of 3-(3-chlorophenyl)-3-oxopropanenitrile (1.0 g, 5.57 mmol) and NH2OH·HCl (0.464 g, 6.68 mmol) in water (15 mL) was added NaOH (0.446 g, 11.14 mmol) portion wise at 0°C. The reaction mixture was heated to 100°C for 3 h. The resulting reaction mixture was cooled to rt and poured into water (40 ml) then extracted with EtOAc (4 x 20 mL). The combined organic layer was dried over Na_2SO_4, filtered and concentrated under reduced pressure. The resulting residue was purified by flash column chromatography (32% EtOAc in hexane) yielding 3-(4-chlorophenyl)isoxazol-5-amine (444 mg) with a yield of 41%. ^{1}H NMR (400 MHz, CDCl3) δ 7.73 (t, J = 1.8 Hz, 1H), 7.63 (dt, J = 7.2, 1.6 Hz, 1H), 7.44 – 7.32 (m, 2H), 5.44 (s, 1H), 4.68 (s, 2H). ^{13}C NMR (101 MHz, CDCl3) δ 169.1, 162.8, 134.7, 131.5, 130.1, 129.8, 126.9, 124.7, 78.1.

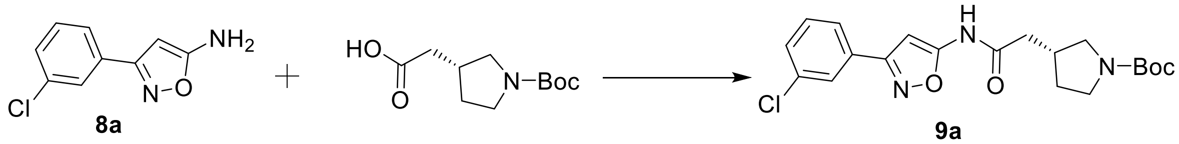

8a 9a

***tert*-butyl (R)-3-(2-((3-(3-chlorophenyl)isoxazol-5-yl)amino)-2-oxoethyl)pyrrolidine-1-carboxylate(9a):**

To a solution of 3-(3-chlorophenyl)isoxazol-5-amine (0.425 g , 2.18 mmol) and (R)-2-(1-(tert-butoxycarbonyl)pyrrolidin-3-yl)acetic acid (0.501 g , 2.18 mmol) in pyridine (15mL) was added $POCl_3$ (0.611 mL, 6.54 mmol) dropwise at 0°C. The reaction mixture was stirred for 30 min at rt. The resulting reaction mixture was poured into water (150 mL) and extracted with EtOAc (3 x 25 mL). The combined organic layer was dried over Na_2SO_4, filtered and concentrated under reduced pressure. The resulting residue was purified by flash column chromatography (36% EtOAc in hexane) yielding compound **9a** (380 mg) with 43% yield. ^{1}H NMR (400 MHz, DMSO) δ 11.79 (s, 1H), 7.92 (d, *J* = 2.4 Hz, 1H), 7.85 (dq, *J* = 7.3, 1.4 Hz, 1H), 7.62 – 7.49 (m, 2H), 6.84 (d, *J* = 2.3 Hz, 1H), 3.49 (q, *J* = 10.6 Hz, 1H), 3.18 (q, *J* = 9.6 Hz, 1H), 2.95 – 2.85 (m, 1H), 2.53 (d, *J* = 5.0 Hz, 4H), 1.99 (s, 1H), 1.54 (d, *J* = 11.4 Hz, 1H), 1.40 (s, 9H). ^{13}C NMR (101 MHz, DMSO) δ 169.2, 162.8, 162.0, 154.0, 134.3, 131.5, 131.3, 130.6, 126.6, 125.7, 86.6, 78.7, 51.2, 45.5, 40.4, 40.2, 40.0, 39.8, 39.6, 39.3, 35.4, 34.6, 31.3, 30.5, 28.7.

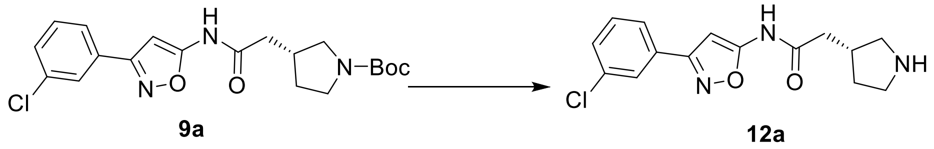

(R)-*N*-(3-(3-chlorophenyl)isoxazol-5-yl)-2-(pyrrolidin-3-yl)acetamide(12a): To a solution of **9a** (330 mg, 0.81 mmol, 1.0 equiv.) in DCM (4 mL) was added TFA (0.6 mL) and the solution stirred at room temperature for 3 h. The reaction mixture was diluted with DCM (10 mL) and concentrated under reduced pressure to yield a crude oil that was triturated with Et_2O. The recovered solid was dried under high vacuum to yield compound **12a** as an off-white solid which was used without further purification.

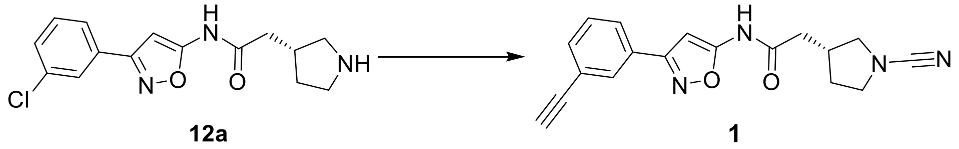

(R)-2-(1-cyanopyrrolidin-3-yl)-N-(3-(3-ethynylphenyl)isoxazol-5-yl)acetamide(1):

A solution of crude **12a** and K_2CO_3 (337 mg, 2.44 mmol, 3.0 equiv.) was prepared in dry THF (4 mL) and cooled to -20°C. A solution of BrCN in DCM (3 M, 325 µL, 0.97 mmol, 1.2 equiv.) was added dropwise and the reaction mixture allowed to warm to room temperature and stirred for 1 h. The resulting mixture was added to water (5 mL) and extracted with EtOAc (3 × 5 mL). The combined organic phases were washed with brine (5 mL), dried over $MgSO_4$, filtered and concentrated under reduced pressure. The crude material was purified by automated flash chromatography yielding compound **1** (234 mg) with 90%.^{1}H NMR (400 MHz, $CDCl_3$) δ 9.66 (s, 1H), 7.81 (t, *J* = 1.9 Hz, 1H), 7.70 (dt, *J* = 7.2, 1.6 Hz, 1H), 7.49 – 7.34 (m, 2H), 6.72 (s, 1H), 3.71 (dd, *J* = 9.7, 6.9 Hz, 1H), 3.62 – 3.46 (m, 2H), 3.22 (dd, *J* = 9.7, 6.4 Hz, 1H), 2.84 (hept, *J* = 7.0 Hz, 1H), 2.61 (d, *J* = 7.3 Hz, 2H), 2.28 – 2.15 (m, 1H), 1.82(s, 1H), 1.80 – 1.69 (m, 1H). ^{13}C NMR (101 MHz, $CDCl_3$) δ 167.4, 162.7, 160.9, 135.0, 130.6, 130.4, 130.3, 126.9, 125.0, 118.0, 87.3, 77.4, 77.1, 76.7, 55.3, 49.7, 38.9, 35.3, 31.2.

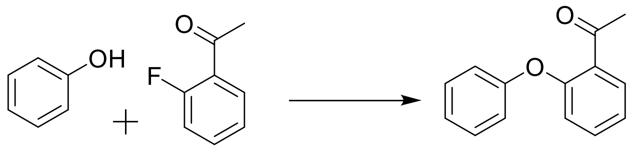

13a

1-(2-Phenoxyphenyl)ethan-1-one(13a): The phenol (17.34 mmol, 1.1 equiv) and anhydrous K_2CO_3 (17.34 mmol, 1.1 equiv) were added to a solution of 2-fluoroacetophenone (15.763 mmol, 1.0 equiv) in DMA (20 mL) and the resulting mixture was slowly heated to 170 °C. After stirring at that temperature for 4 h and cooling to room temperature the reaction mixture was poured into H_2O (100 mL) and the aqueous layer was extracted with DCM (3×40 mL). After drying over $MgSO_4$, filtration and concentration in *vacuo*, the product 13a was obtained for 2.1 g with a yield of 57%.^{1}H NMR (400 MHz, $CDCl_3$) δ 7.88 (dt, *J* = 7.8, 1.4 Hz, 1H), 7.48 – 7.36 (m, 3H), 7.22 – 7.14 (m, 2H), 7.05 (dt, *J* = 8.5, 1.2 Hz, 2H), 6.93 (dd, *J* = 8.3, 1.2 Hz, 1H), 2.67 (s, 3H). ^{13}C NMR (101 MHz, $CDCl_3$) δ 199.0, 156.5, 156.5, 133.7, 130.6, 130.1, 123.9, 123.5, 119.4, 31.7, 31.7, 31.6, 31.6.

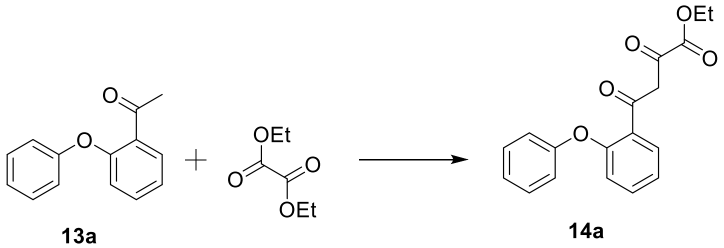

13a **14a**

Ethyl 2,4-dioxo-4-(2-phenoxyphenyl)butanoate(14a): 3.77 mmol of compound **13a** and 0.564 ml (4.15 mmol) of diethyl oxalate were dissolved in 3 ml of anhydrous THF. This solution was added dropwise to the stirred suspension of 136 mg (5.65 mmol) of sodium hydride in 4 ml of anhydrous THF. Heating and evolution of H_2 occurred, and sodium salt of diketoester began to precipitate. When addition was completed, heating and stirring of reaction mixture with reflux condenser were continued for 10–30 min, until evolution of gas in bubble counter disappeared. Then THF was evaporated under reduced pressure. 20 ml of ice water, 10 ml of CH_2Cl_2 and 0.8 ml of H_2SO_4 were added to the residue of sodium salt of diketoester. Mixture was shacked until dissolution of solid and transferred to separation funnel. Organic layer was evaporated under reduced pressure to obtain compound **14a** (848 mg) in 72% yield. ^{1}H NMR (400 MHz, $CDCl_3$) δ 15.08 (s, 1H), 7.96 (dt, *J* = 7.8, 1.3 Hz, 1H), 7.51 – 7.43 (m, 1H), 7.37 (ddd, *J* = 8.5, 7.4, 0.9 Hz, 2H), 7.30 (s, 1H), 7.25 – 7.19 (m, 1H), 7.16 (td, *J* = 7.5, 1.1 Hz, 1H), 7.06 – 6.99 (m, 2H), 6.93 (d, *J* = 8.3 Hz, 1H), 4.31 (qd, *J* = 7.1, 0.9 Hz, 2H), 1.32 (td, *J* = 7.1, 0.9 Hz, 3H). ^{13}C NMR (101 MHz, $CDCl_3$) δ 190.1, 168.9, 162.2, 156.6, 156.2, 134.3, 130.7, 130.1, 127.3, 124.1, 123.7, 119.4, 119.0, 103.1, 62.4, 14.0.

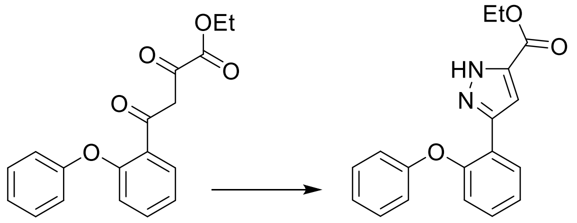

14a **15a**

Ethyl 3-(2-phenoxyphenyl)-1H-pyrazole-5-carboxylate(15a): Hydrazine dihydrochloride (N_2H_4·2HCl) (967 mg, 9.21 mmol) dissolved in ethanol was added to compound **14a** (2.4 g, 6.14 mmol) and refluxed

for 4 hours. After the completion of the reaction, ethanol was evaporated under high vacuum conditions. 15-20 mL water was mixed to the above reaction residue, followed by extraction with 4x10 mL of ethyl acetate. The four fractions of organic layer were mixed with each other and dried on anhydrous Na_2SO_4. The ethyl acetate, organic solvent was evaporated by rotavapor under reduced pressure to give crude compound. A silica gel column chromatography technique with 7:3 v/v hexane and ethyl acetate solvent system were applied to the above crude to afford compound **15a** (1.6 g, 86% yield). ^{1}H NMR (400 MHz, $CDCl_3$) δ 11.77 (s, 1H), 7.85 (dd, *J* = 7.7, 1.8 Hz, 1H), 7.44 – 7.36 (m, 2H), 7.33 – 7.25 (m, 2H), 7.20 (td, *J* = 7.7, 3.2 Hz, 2H), 7.08 (dt, *J* = 8.0, 1.2 Hz, 2H), 6.92 (dd, *J* = 8.3, 1.5 Hz, 1H), 4.46 – 4.34 (m, 2H), 1.41 (td, *J* = 7.1, 1.1 Hz, 3H). ^{13}C NMR (101 MHz, $CDCl_3$) δ 162.1, 155.1, 154.2, 130.2, 129.8, 128.5, 124.5, 123.9, 119.5, 118.7, 106.7, 61.1, 14.3.

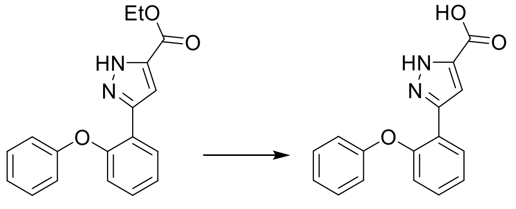

15a **16a**

3-(2-Phenoxyphenyl)-1H-pyrazole-5-carboxylic acid(16a): LiOH (37 mg, 1.54 mmol) was mixed with a solution of compound **15a** (190 mg, 0.616 mmol) in THF (3 ml) and the reaction mass was stirred for over 6-7 hour time period at reflux. Add water and HCl to make pH 4-5 and precipitate was filtered off and washed with water to get 147 mg compound **16a** with 85% yield. ^{1}H NMR (400 MHz, DMSO) δ 13.58 (s, 1H), 7.99 (d, *J* = 7.7 Hz, 1H), 7.43 – 7.34 (m, 3H), 7.28 (td, *J* = 7.6, 1.2 Hz, 1H), 7.13 (t, *J* = 7.4 Hz, 1H), 7.04 – 6.95 (m, 3H). ^{13}C NMR (101 MHz, DMSO) δ 153.2, 130.6, 128.8, 124.8, 123.7, 120.6, 118.3.

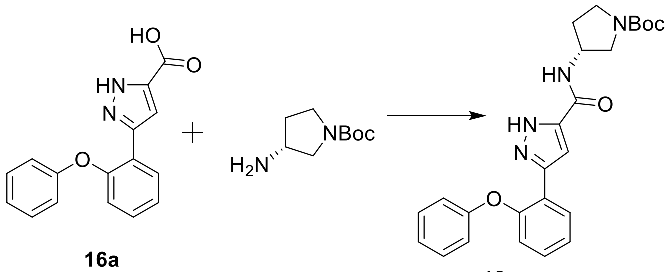

16a **18**

tert-Butyl (R)-3-(3-(2-phenoxyphenyl)-1H-pyrazole-5-carboxamido)pyrrolidine-1-carboxylate(18): Compound **16a** (170mg, 0.616 mmol), tert-butyl (R)-3-aminopyrrolidine-1-carboxylate (125 ul, 0.73 mmol), HATU (286 mg, 0.75 mmol) was dissolved in DMF (2.5 mL), following by DIPEA (215 ul, 1.232 mmol) addition. The resulting mixture was stirred at room temperature for overnight. Water was added to the reaction mixture. The resulting mixture was extracted with ethylacetate 3 times. The ethyl acetate organic solvent was dried on anhydrous Na_2SO_4 and evaporated by rotavapor under reduced pressure to give crude compound. Silica gel column chromatography was applied to afford 249 mg compound **18** with a yield of 90%. ^{1}H NMR (400 MHz, $CDCl_3$) δ 11.74 (s, 1H), 7.78 (dd, *J* = 7.7, 1.6 Hz, 1H), 7.39 (t, *J* = 7.8 Hz, 2H), 7.27 (dt, *J* = 8.2, 4.5 Hz, 1H), 7.19 (td, *J* = 7.3, 4.6 Hz, 2H), 7.08 (d, *J* = 7.9 Hz, 3H), 6.89 (d, *J* = 8.2 Hz, 1H), 4.64 (h, *J* = 6.1 Hz, 1H), 3.72 (dd, *J* = 11.4, 6.4 Hz, 1H), 3.48 (dq, *J* = 14.3, 7.3 Hz, 2H), 3.37 – 3.20 (m, 1H), 2.21 (dd, *J* = 12.9, 6.6 Hz, 1H), 2.01 – 1.87 (m, 1H), 1.47 (s, 9H). ^{13}C NMR (101 MHz, $CDCl_3$) δ 161.9, 155.5, 154.6, 154.2, 130.2, 129.8, 128.5, 124.7, 123.9, 119.7, 118.4, 104.2, 79.6, 51.7, 51.0, 49.1, 48.4, 44.2, 43.8, 38.6, 32.0, 31.1, 28.5.

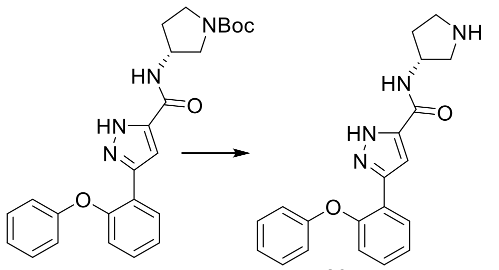

18 20

(R)-3-(2-phenoxyphenyl)-N-(pyrrolidin-3-yl)-1H-pyrazole-5-carboxamide(20): To a solution of compound **18** (100 mg, 0.223 mmol, 1.0 equiv.) in DCM (1.5 mL) was added TFA (0.17 mL) and the solution stirred at room temperature for 3 h. The reaction mixture was diluted with DCM (2 mL) and concentrated under reduced pressure to yield a crude oil that was triturated with Et_2O. The recovered solid was dried under high vacuum to yield compound **20** as an off-white solid which was used without further purification.

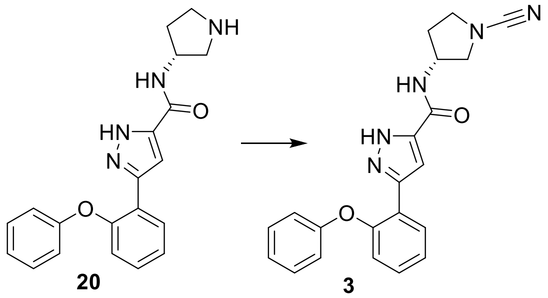

20 3

(R)-N-(1-cyanopyrrolidin-3-yl)-3-(2-phenoxyphenyl)-1H-pyrazole-5-carboxamide(3): A solution of crude compound **20** and K_2CO_3 (93 mg, 0.669 mmol, 3.0 equiv.) was prepared in dry THF (2 mL) and cooled to -20°C. A solution of BrCN in DCM (3 M,89 µL, 0.267 mmol, 1.2 equiv.) was added dropwise and the reaction mixture allowed to warm to room temperature and stirred for 1 h. The resulting mixture was added to water (5 mL) and extracted with EtOAc (3 × 5 mL). The combined organic phases were washed with brine (5 mL), dried over $MgSO_4$, filtered and concentrated under reduced pressure. The crude material was purified by automated flash chromatography to obtain 75 mg the final compound **3** with a yield of 90%. ^{1}H NMR (400 MHz, $CDCl_3$) δ 7.77 (dd, *J* = 7.8, 1.7 Hz, 1H), 7.45 – 7.37 (m, 2H), 7.29 (td, *J* = 7.9, 1.7 Hz, 1H), 7.23 – 7.06 (m, 5H), 6.89 (dd, *J* = 8.3, 1.1 Hz, 1H), 4.68 (h, *J* = 5.8 Hz, 1H), 3.72 (dd, *J* = 10.0, 6.1 Hz, 1H), 3.64 – 3.47 (m, 2H), 3.38 (dd, *J* = 10.0, 4.4 Hz, 1H), 2.31 – 2.20 (m, 1H), 2.09 – 1.96 (m, 1H). ^{13}C NMR (101 MHz, $CDCl_3$) δ 162.0, 155.3, 154.3, 146.2, 142.2, 130.2, 123.0, 128.5, 124.8, 123.9, 119.9, 118.3, 117.0, 104.2, 55.4, 49.1, 48.8, 31.6.

3) ^{1}H and ^{13}C spectra

3-(3-bromophenyl)isoxazol-5-amine(8):

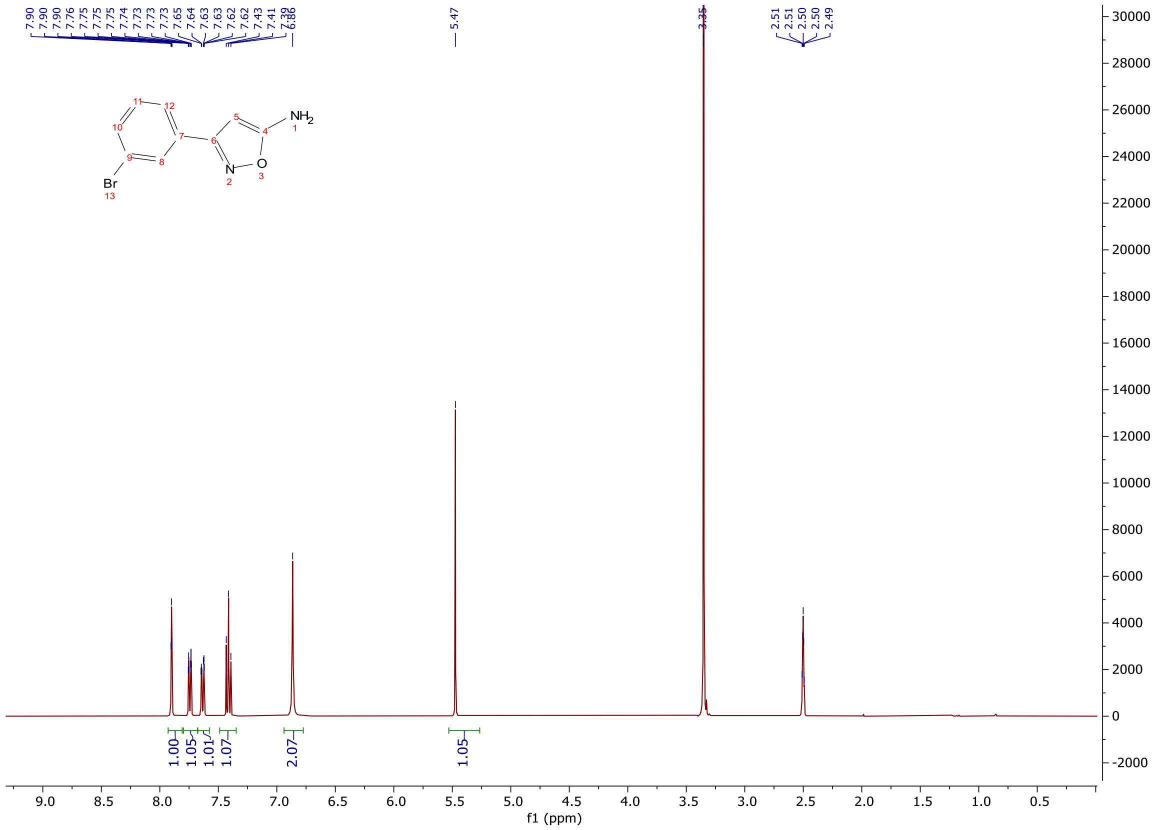

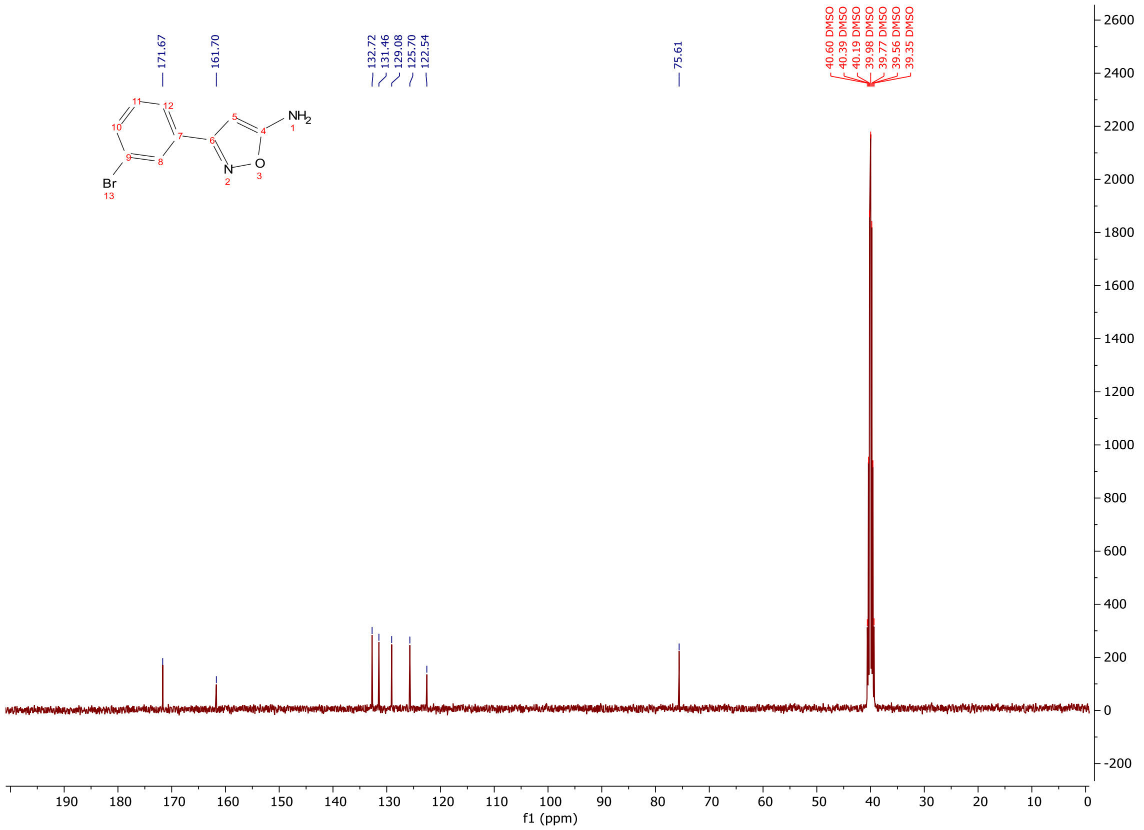

tert-butyl (R)-3-(2-((3-(3-bromophenyl)isoxazol-5-yl)amino)-2-oxoethyl)pyrrolidine-1-carboxylate (9):

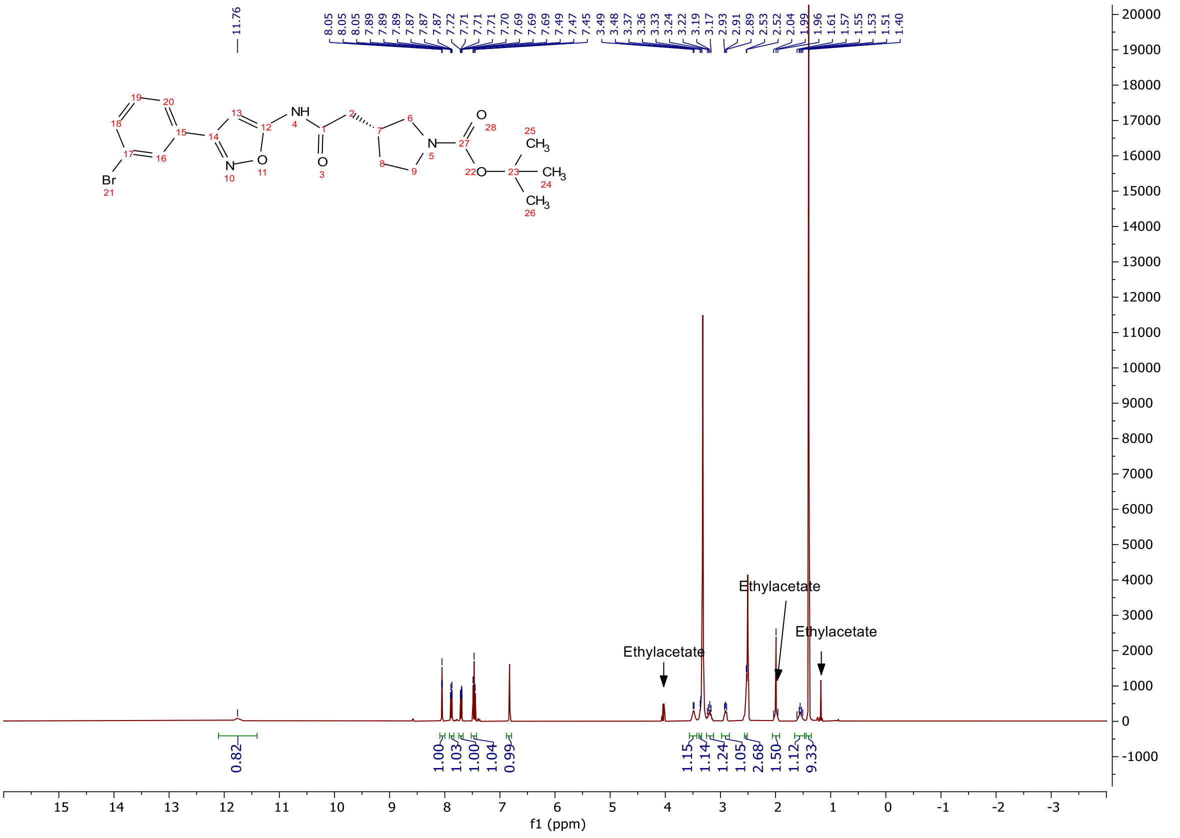

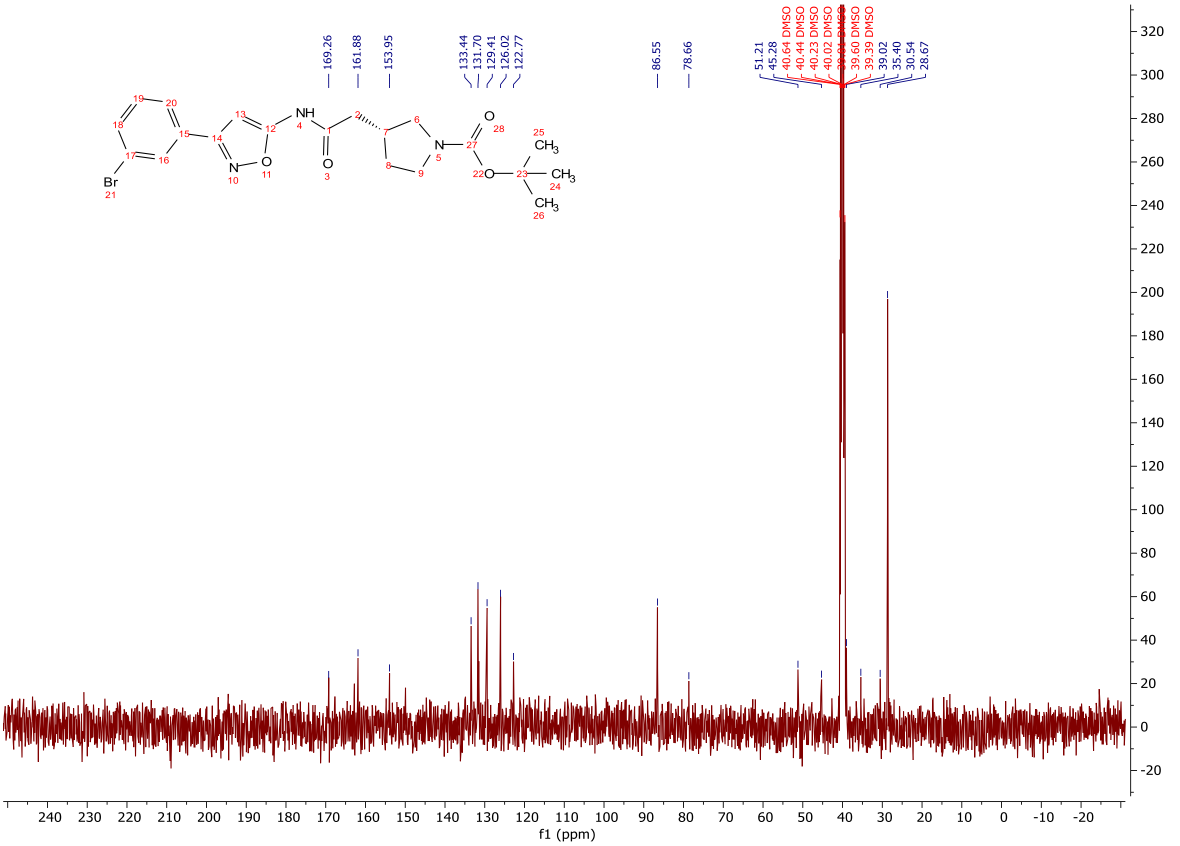

***tert*-butyl (R)-3-(2-oxo-2-((3-(3-((trimethylsilyl)ethynyl)phenyl)isoxazol-5-yl)amino)ethyl)pyrrolidine-1-carboxylate(10)**

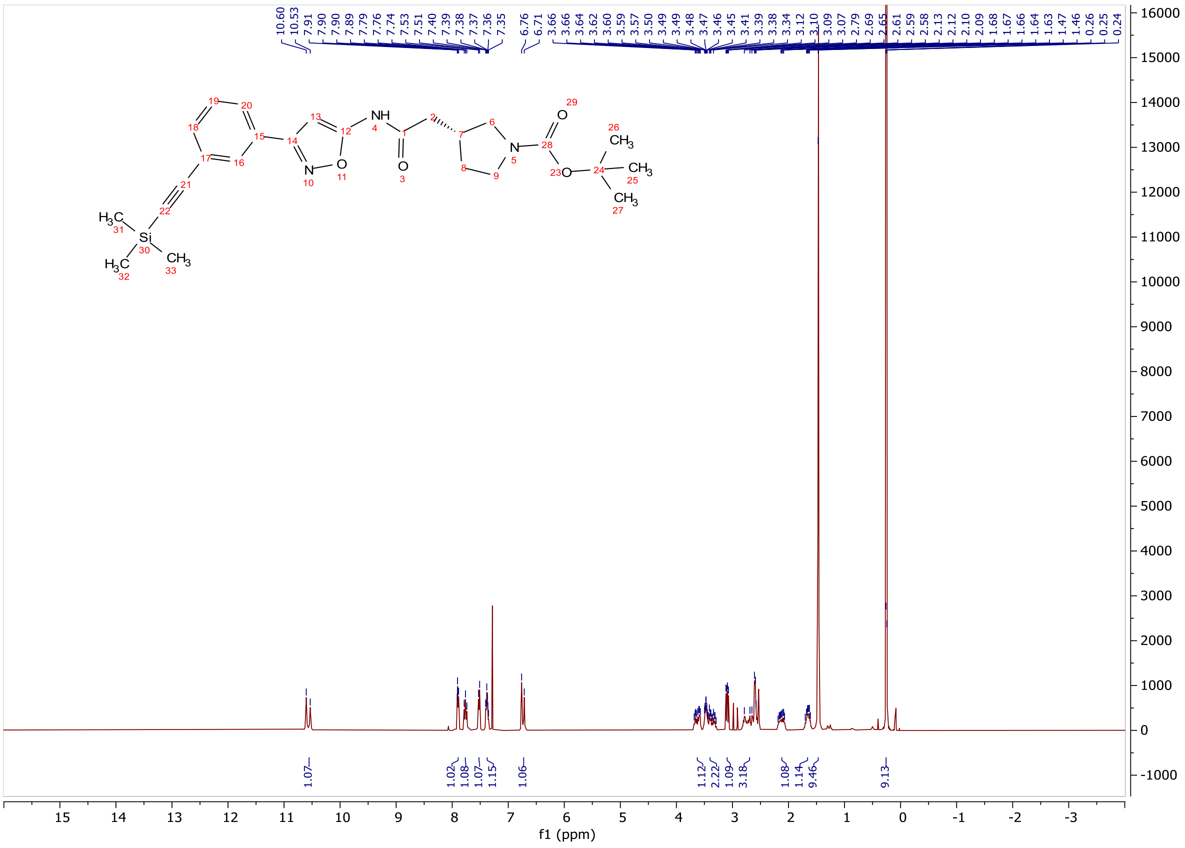

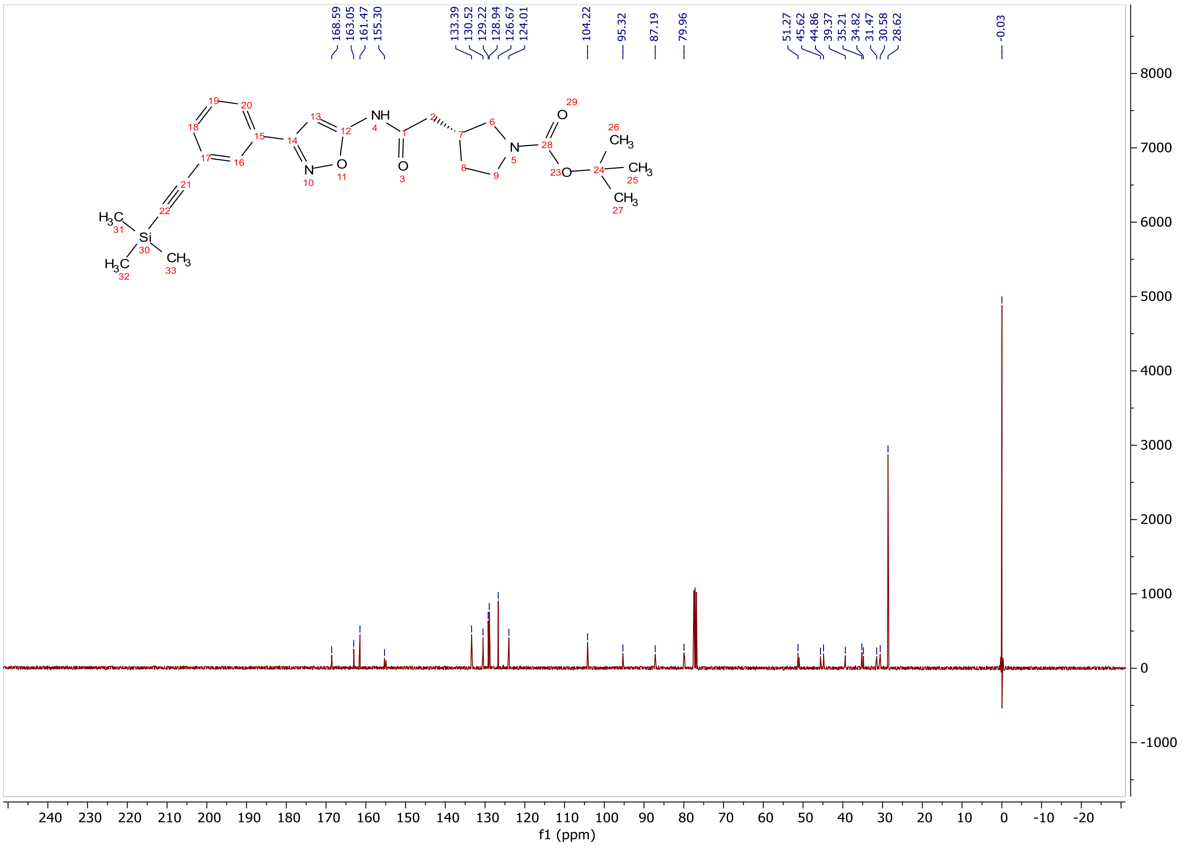

tert-butyl(_R)_-3-(2-((3-(3-ethynylphenyl)isoxazol-5-yl)amino)-2-oxoethyl)pyrrolidine-1-carboxylate (11):

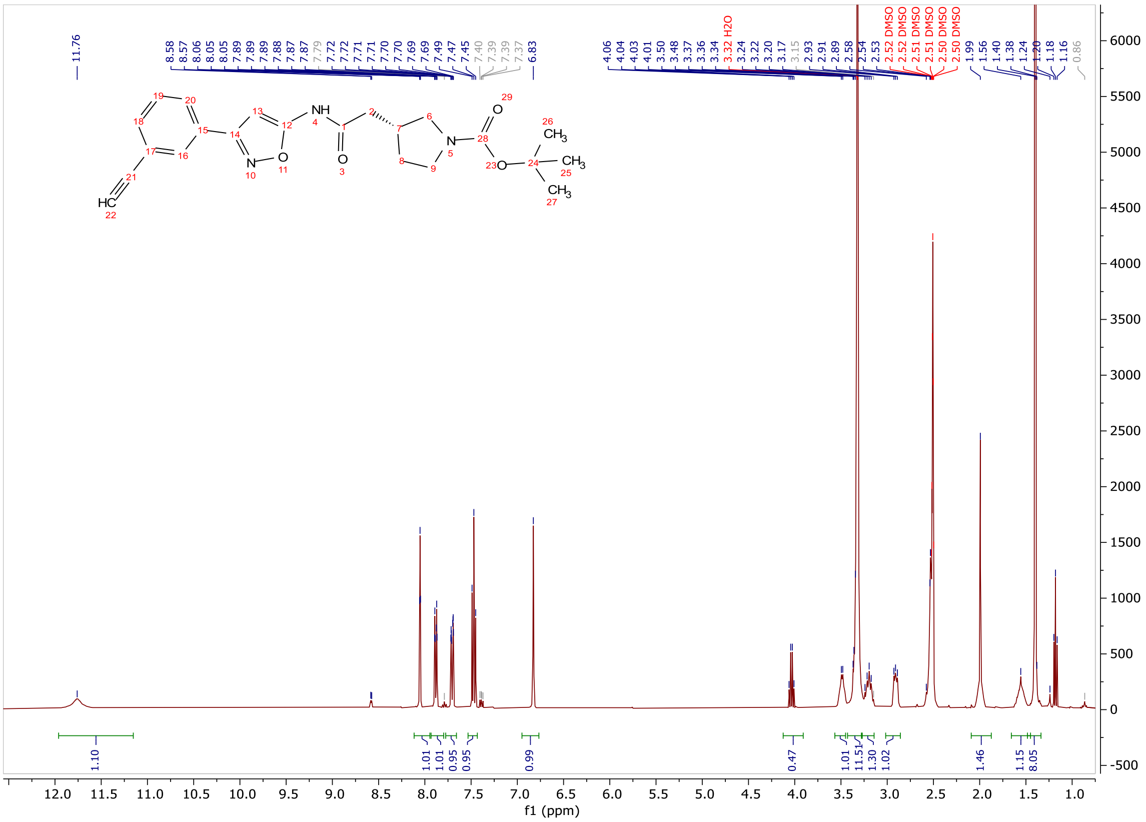

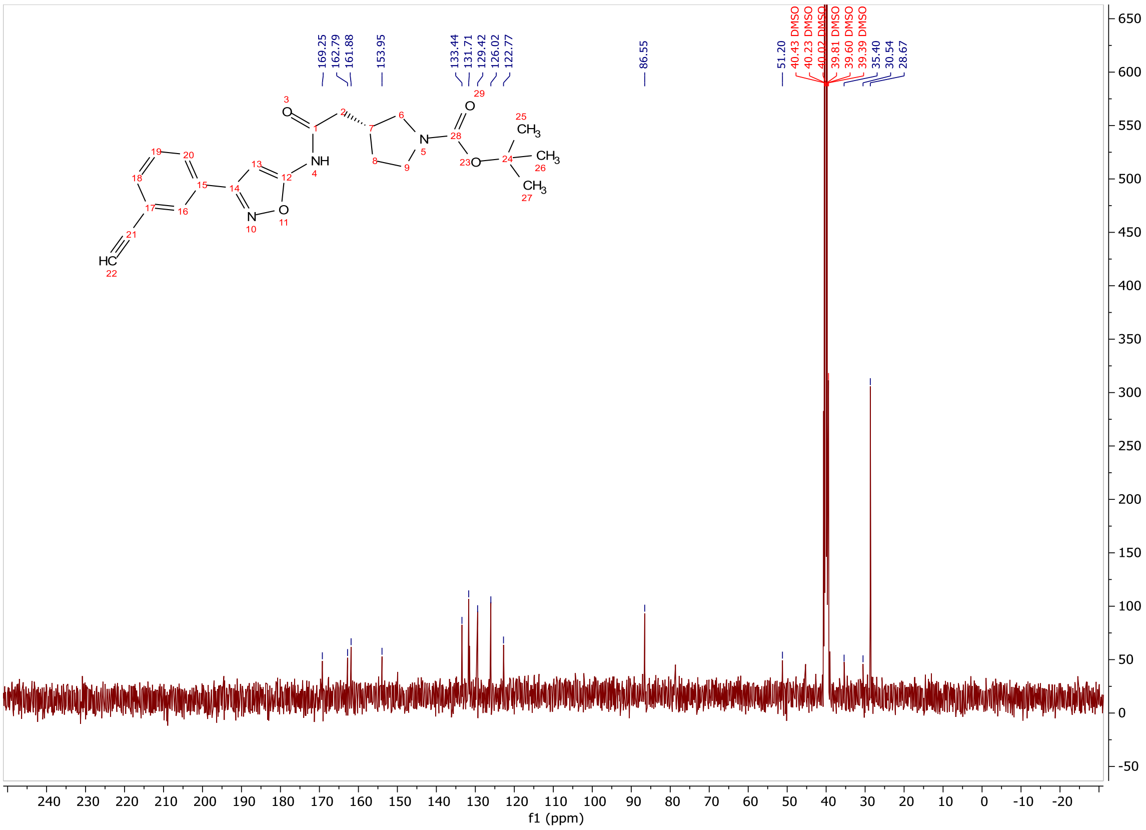

***(R)*-2-(1-cyanopyrrolidin-3-yl)-N-(3-(3-ethynylphenyl)isoxazol-5-yl)acetamide (2, IMP-2587)**

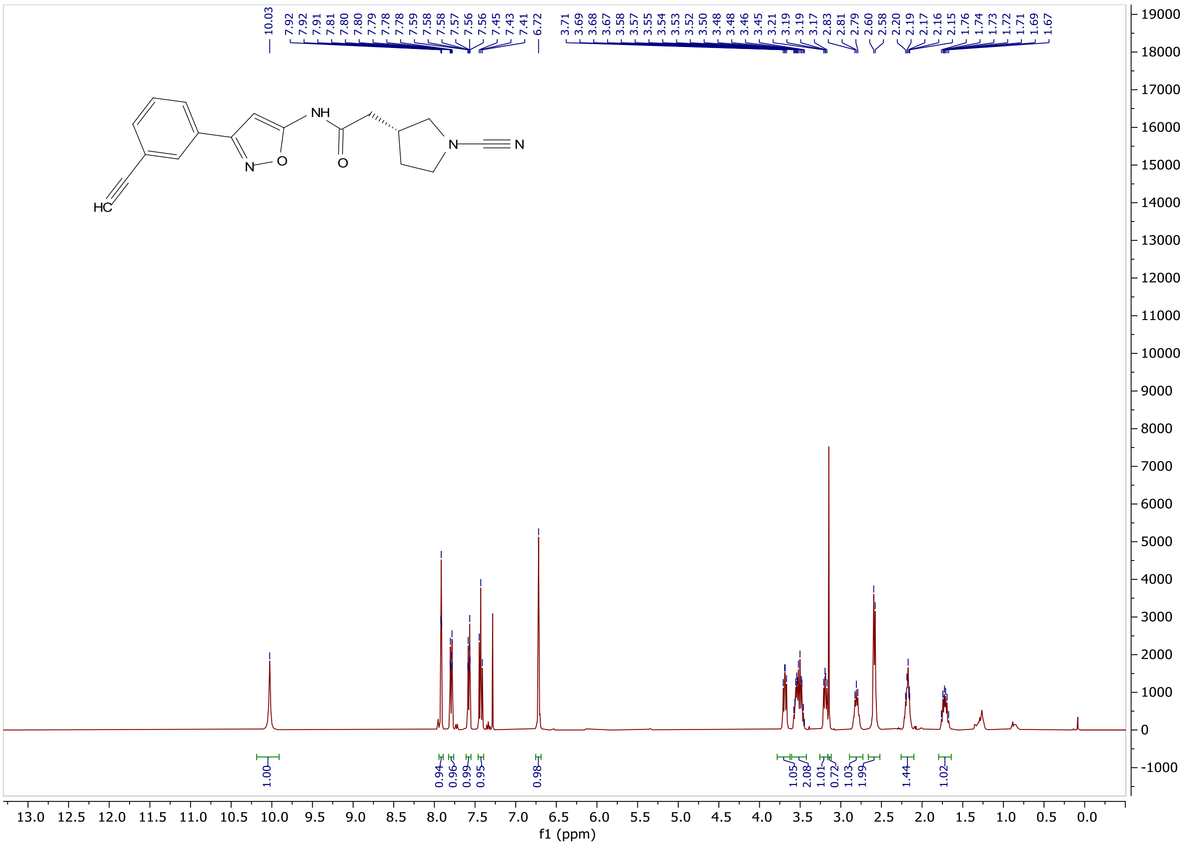

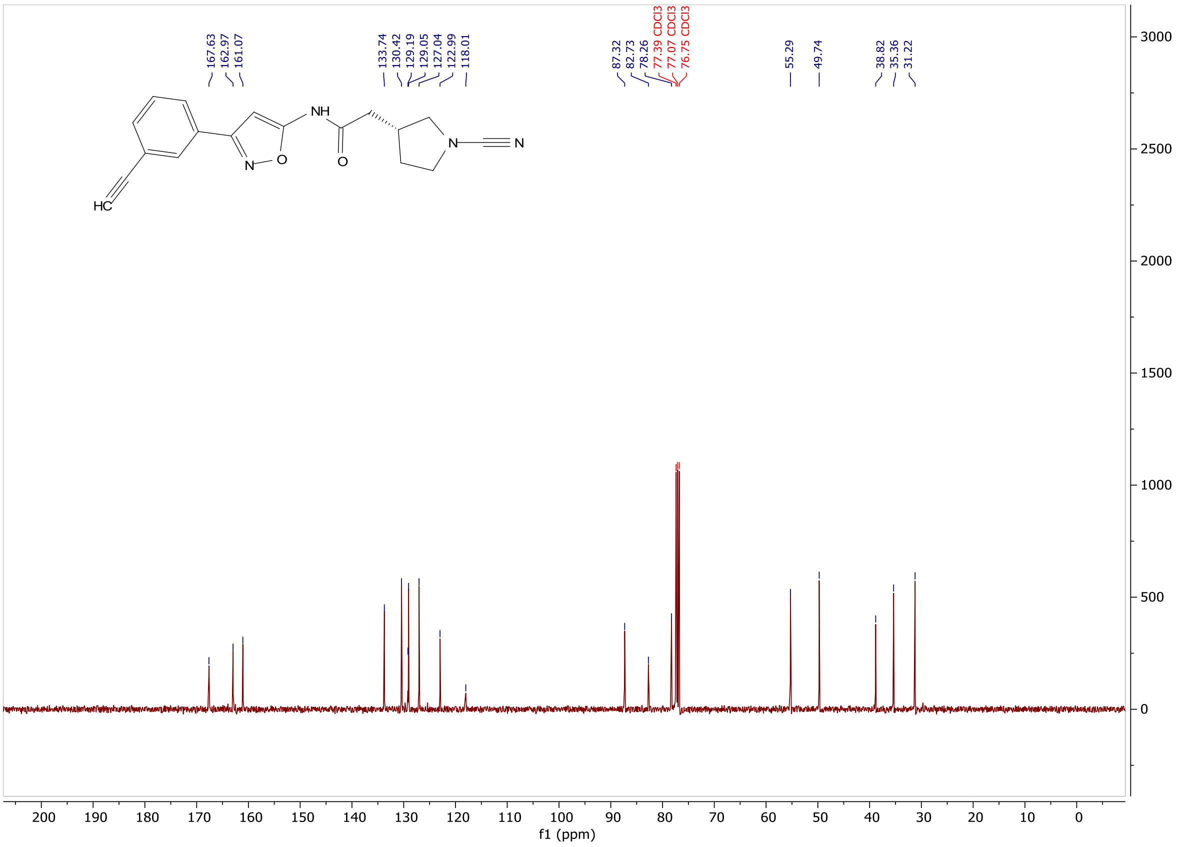

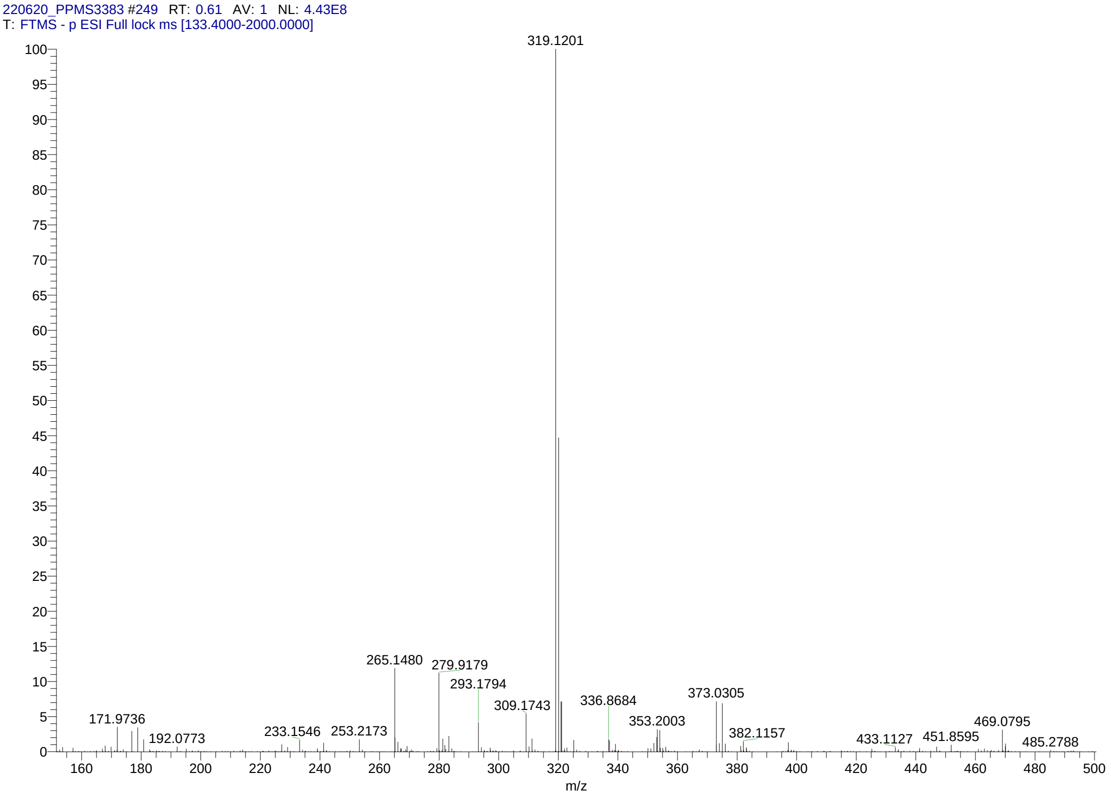

220620_PPMS3383 #249 RT: 0.61 AV: 1 NL: 4.43E8
T: FTMS - p ESI Full lock ms [133.4000-2000.0000]
319.1201
265.1480
279.9179
293.1794
309.1743
336.8684
373.0305
353.2003
171.9736
192.0773
233.1546
253.2173
382.1157
433.1127
451.8595
469.0795
485.2788
m/z

1-(2-(4-bromophenoxy)phenyl)ethan-1-one (13):

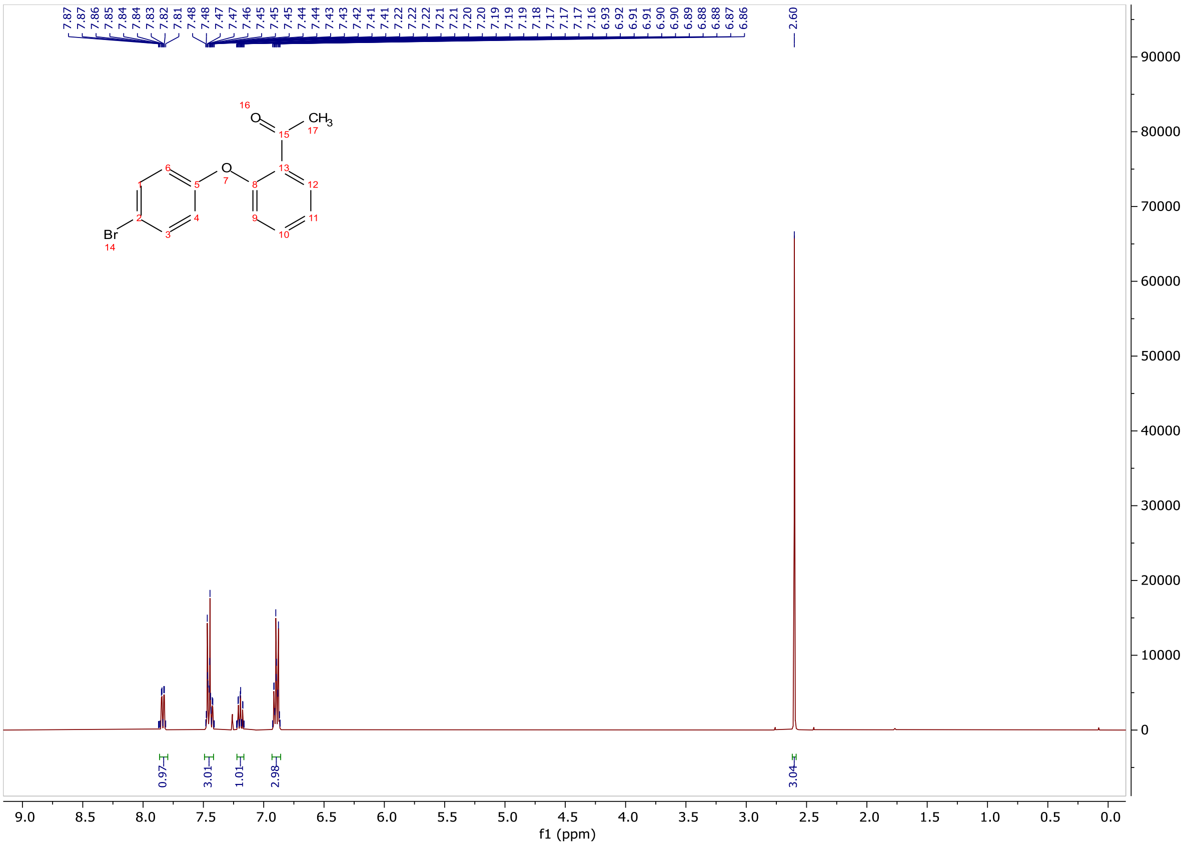

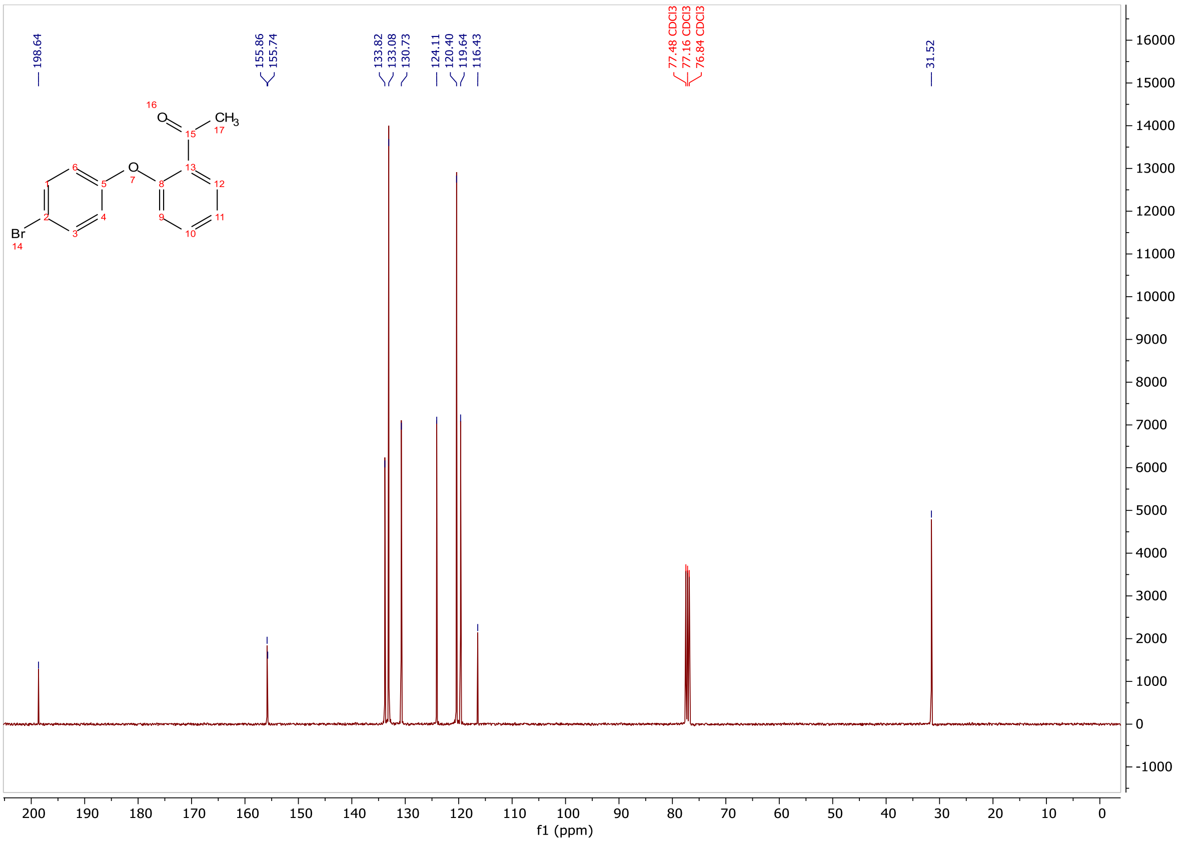

Ethyl 4-(2-(4-bromophenoxy)phenyl)-2,4-dioxobutanoate (14):

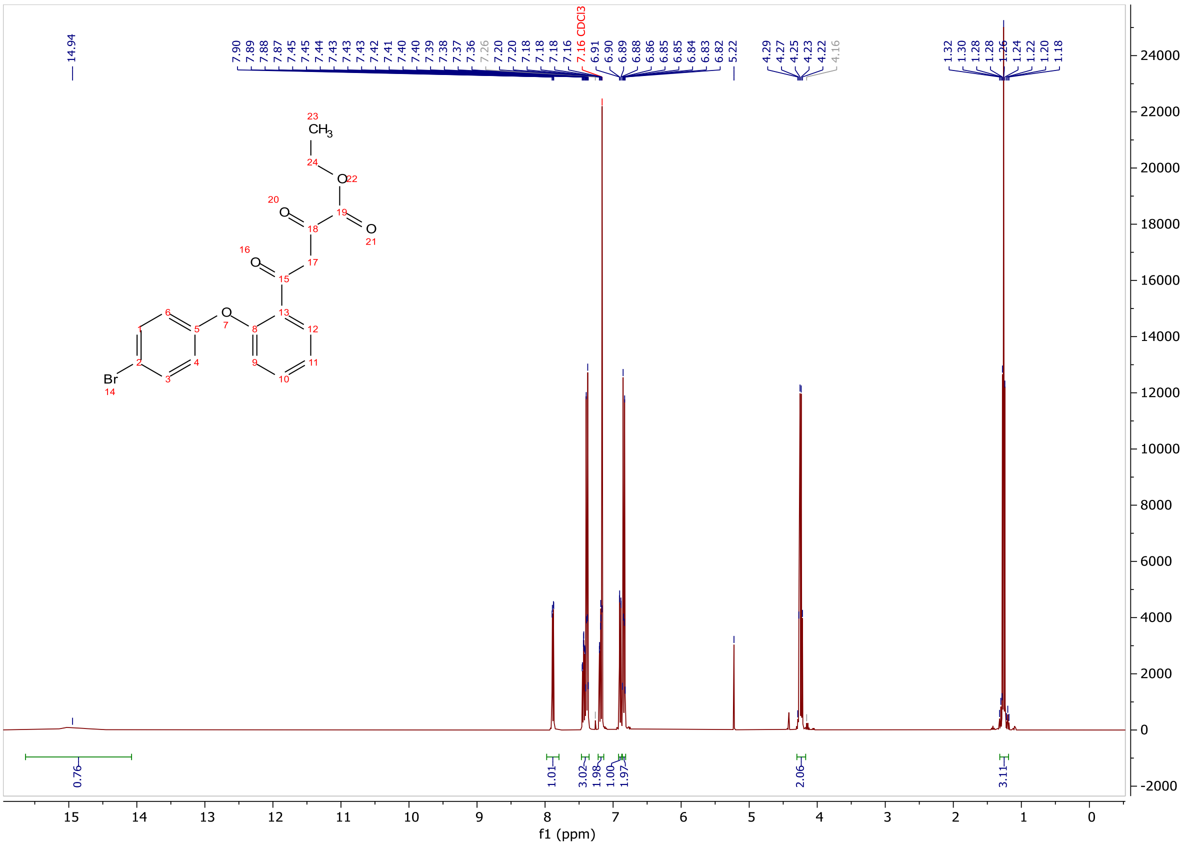

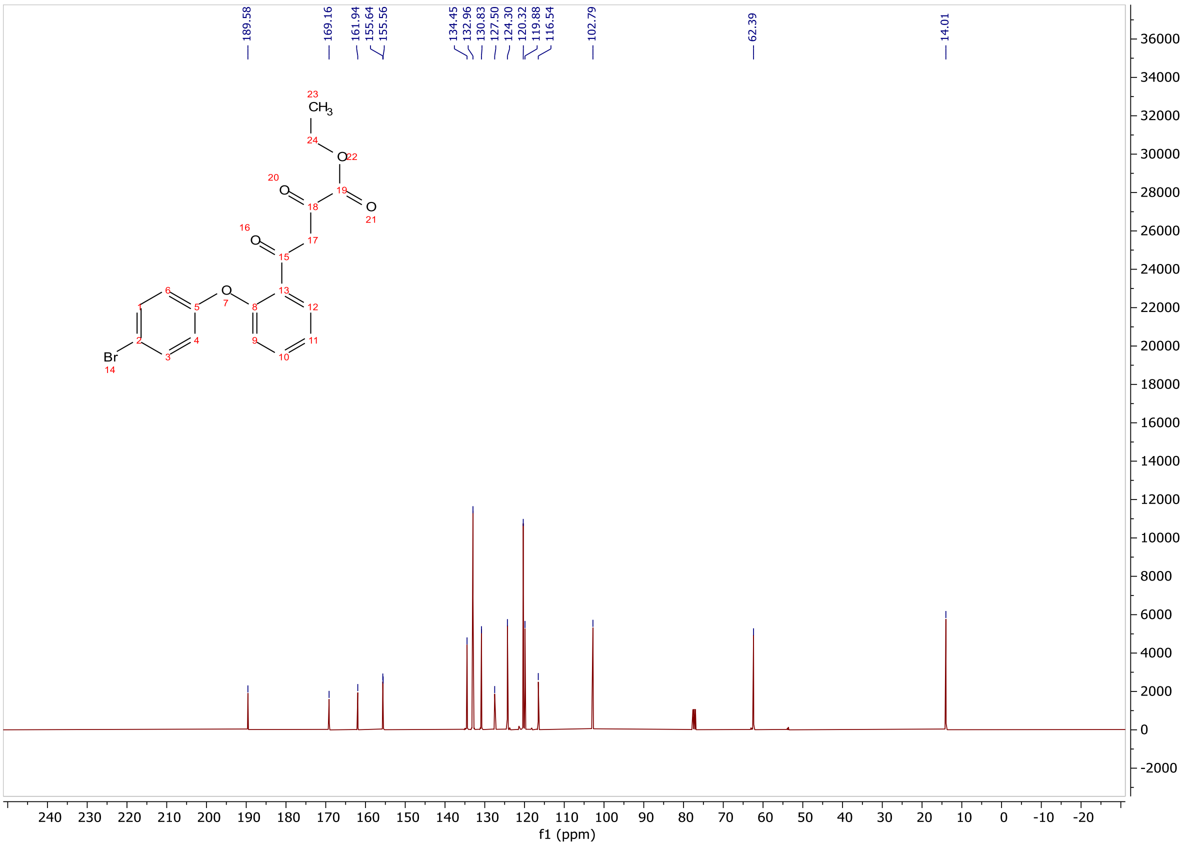

Ethyl 3-(2-(4-bromophenoxy)phenyl)-1H-pyrazole-5-carboxylate (15):

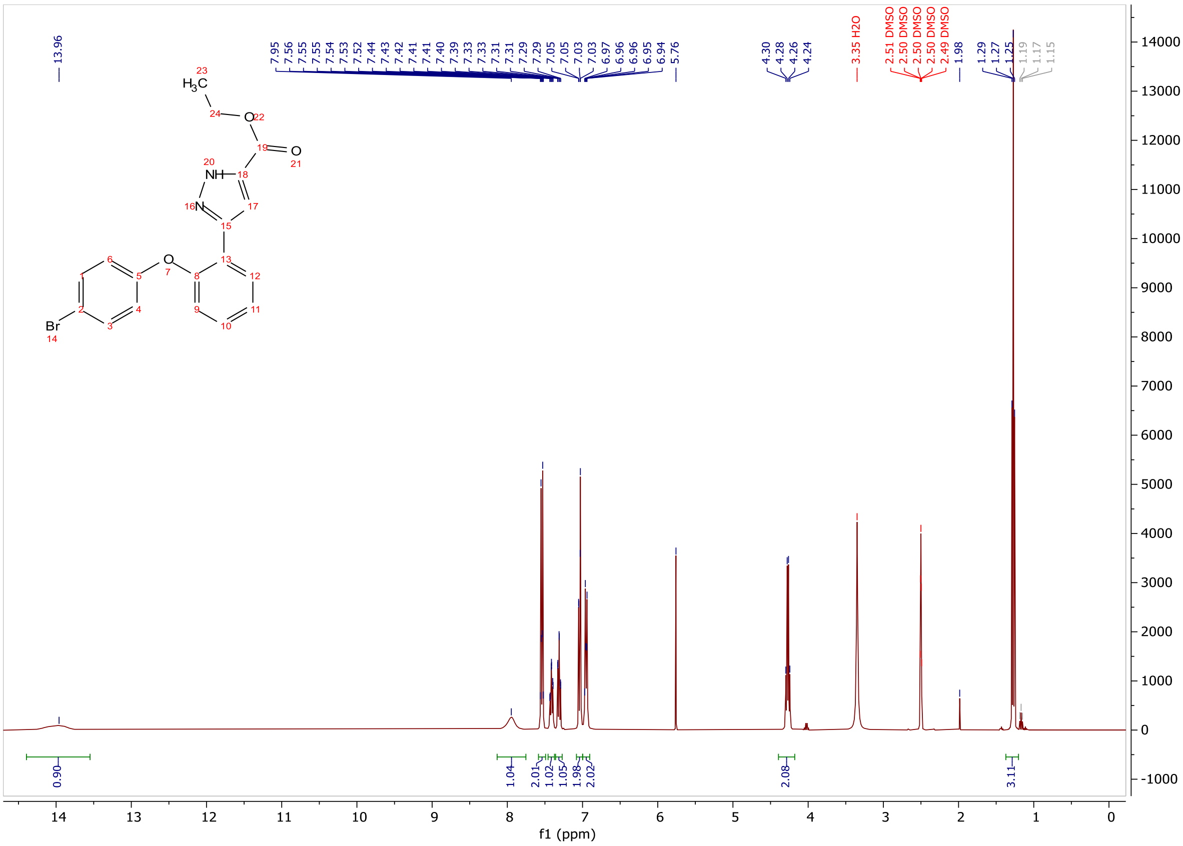

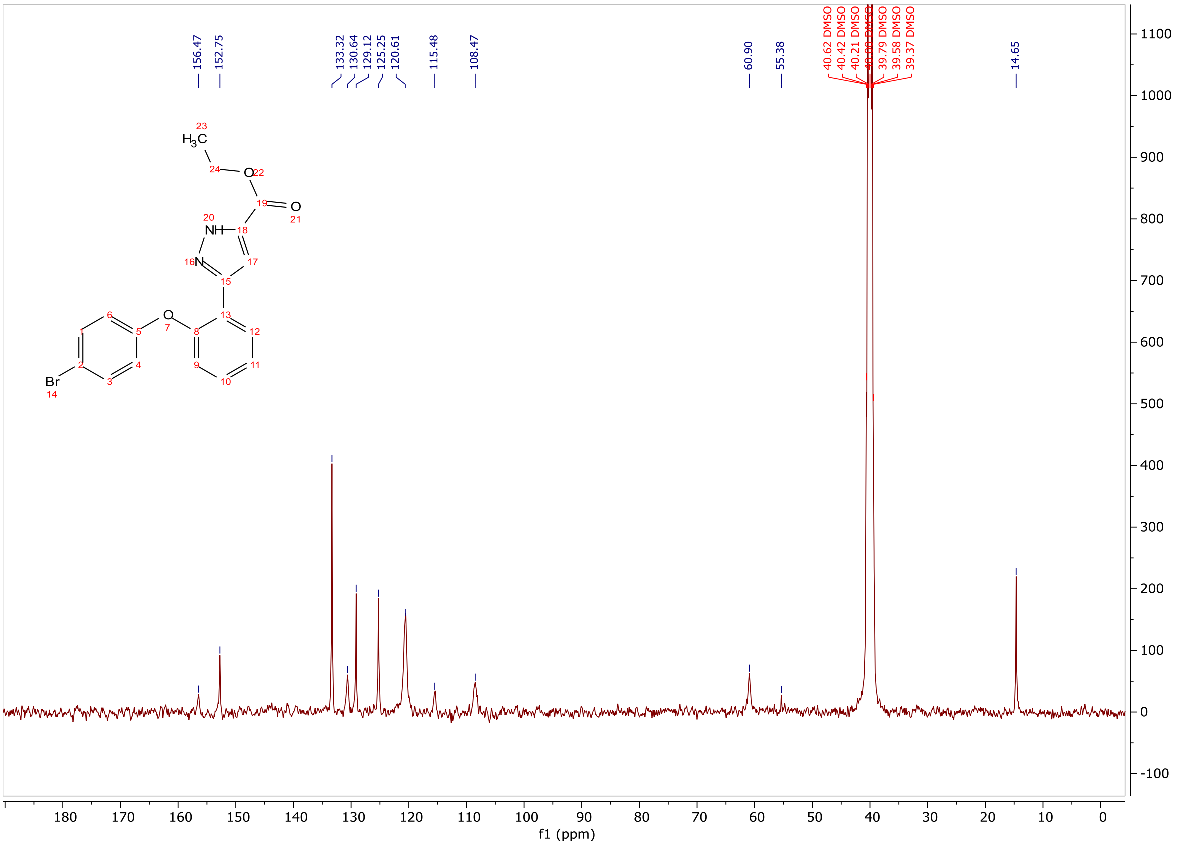

3-(2-(4-Bromophenoxy)phenyl)-1H-pyrazole-5-carboxylic acid (16):

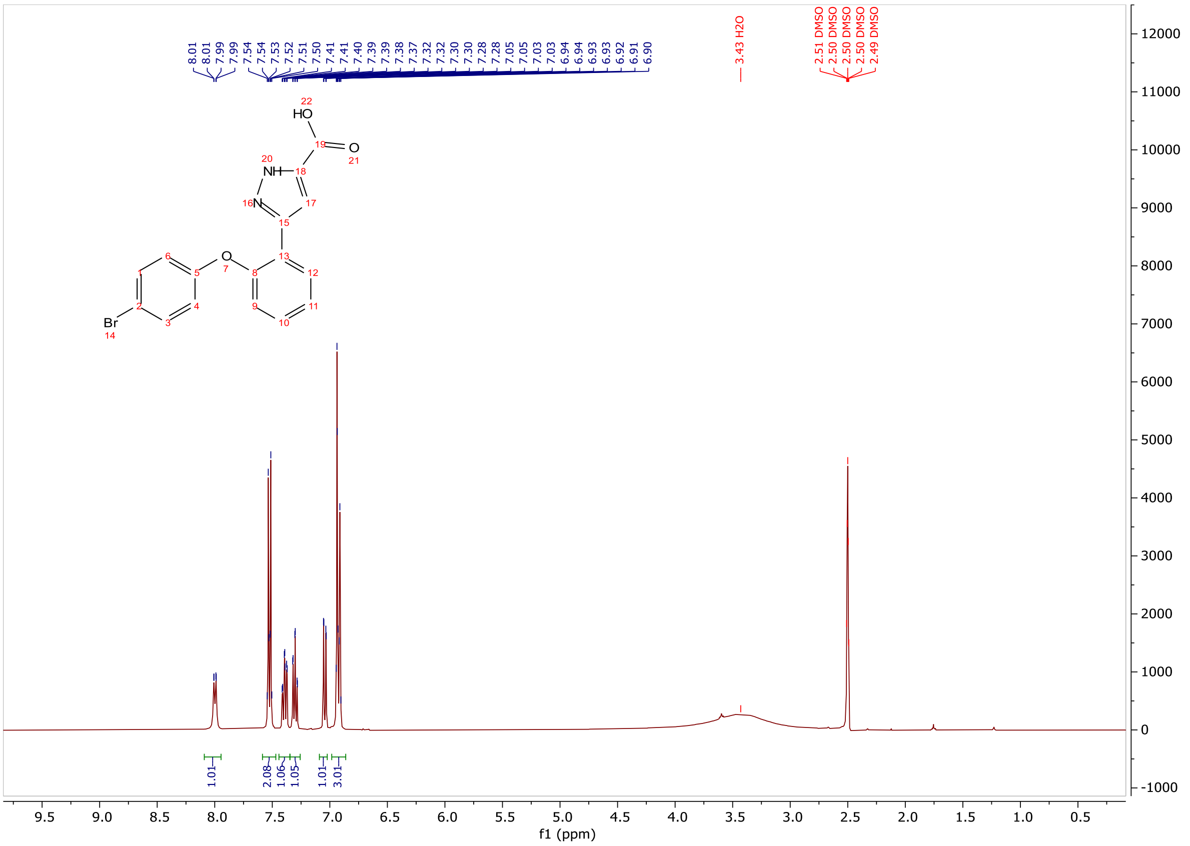

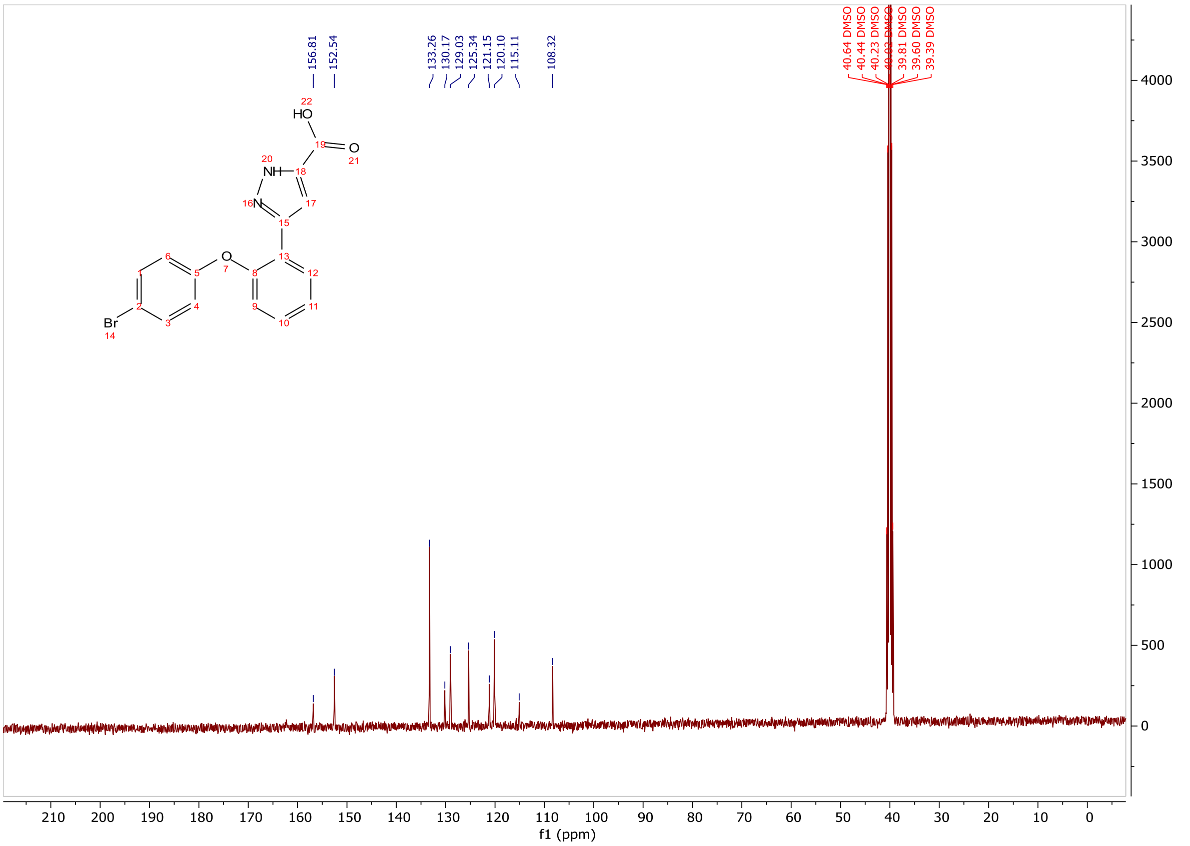

tert-Butyl(R)-3-(3-(2-(4-bromophenoxy)phenyl)-1H-pyrazole-5-carboxamido)pyrrolidine-1-carboxylate (17):

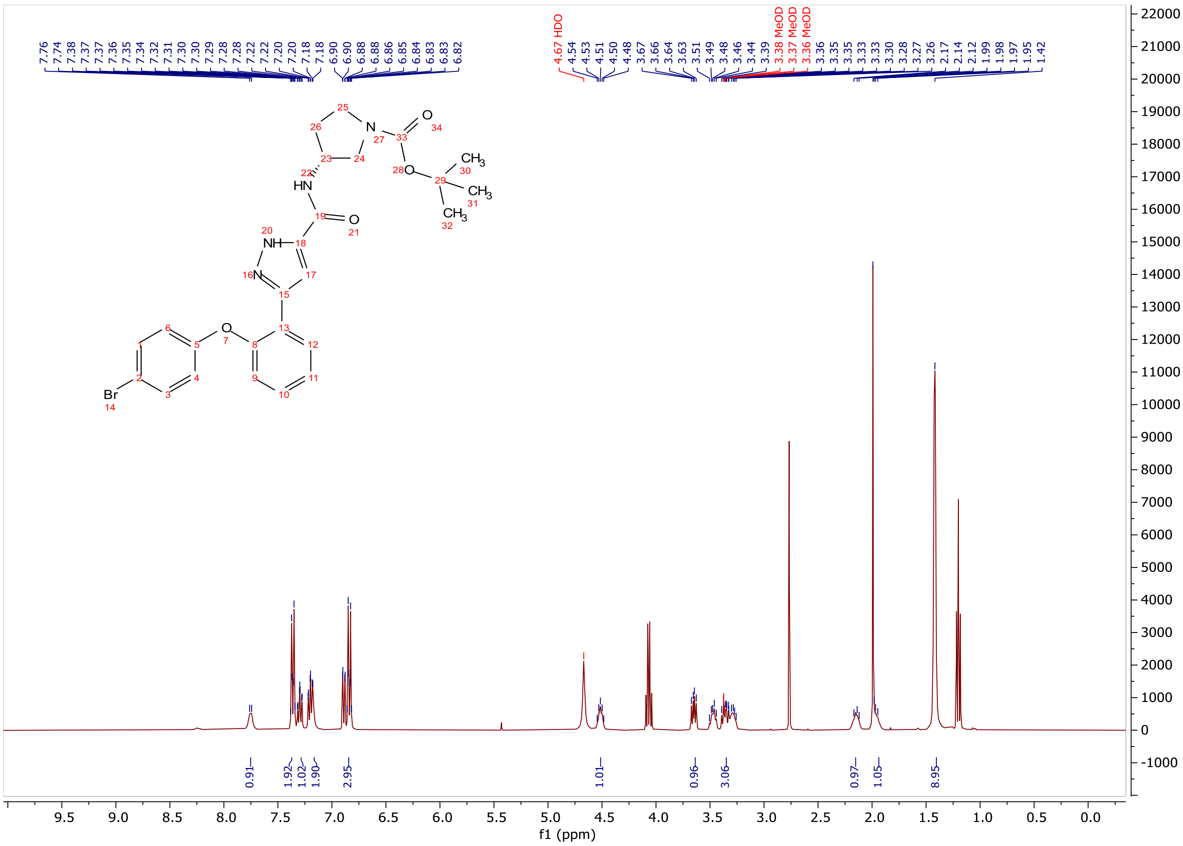

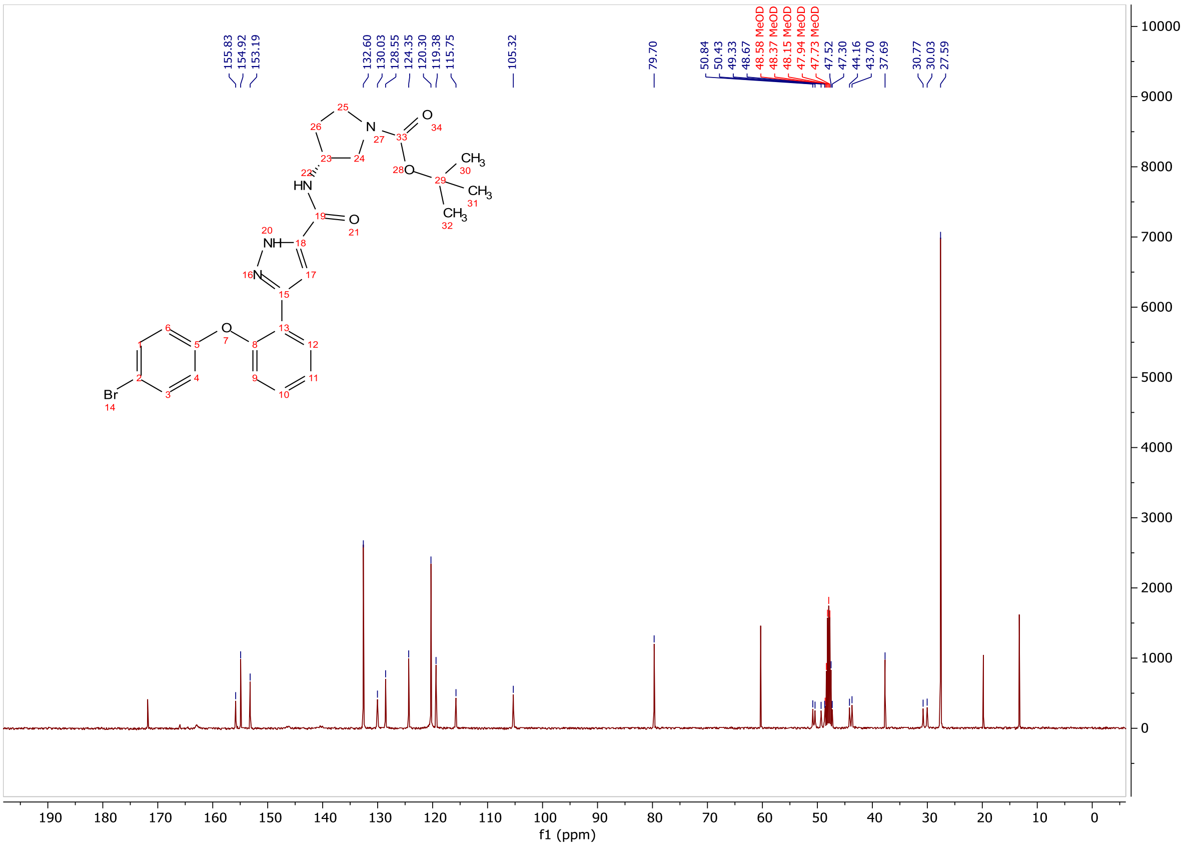

tert-Butyl(R)-3-(3-(2-(4-azidophenoxy)phenyl)-1H-pyrazole-5-carboxamido)pyrrolidine-1-carboxylate (19):

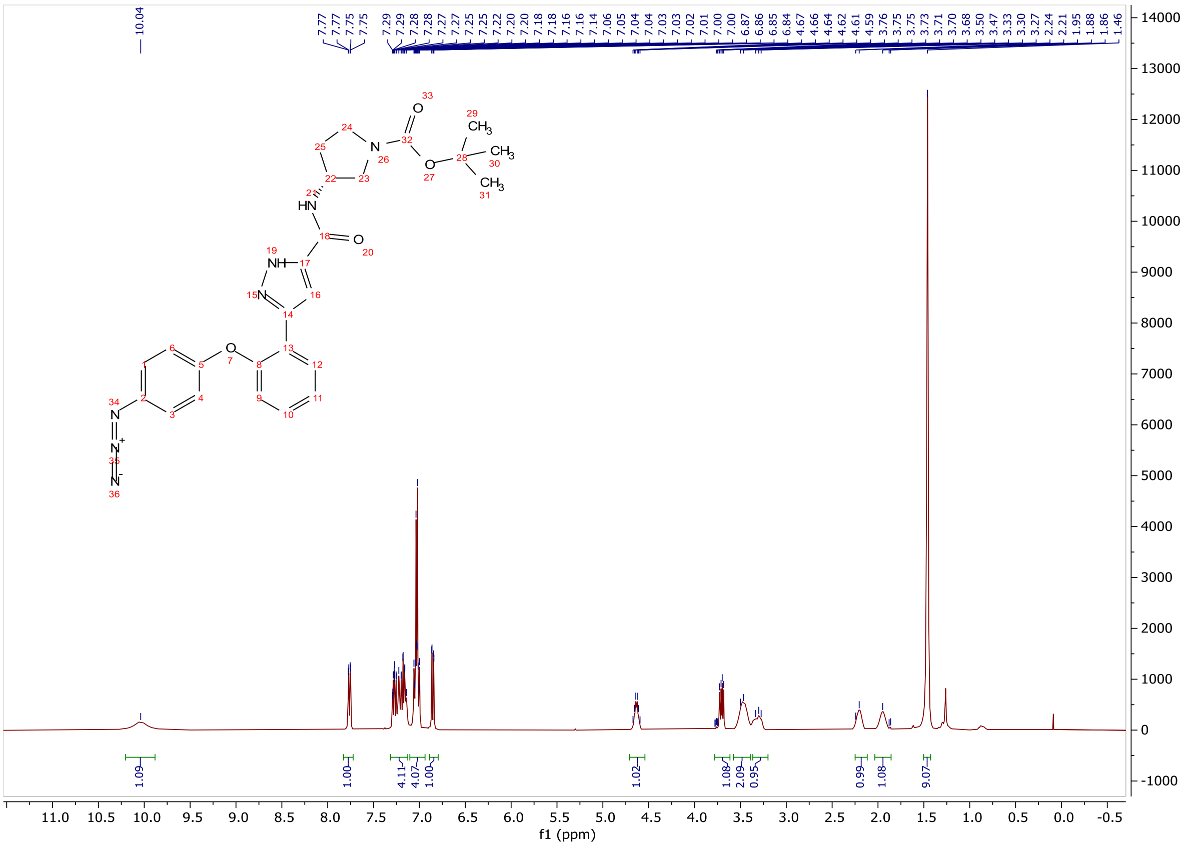

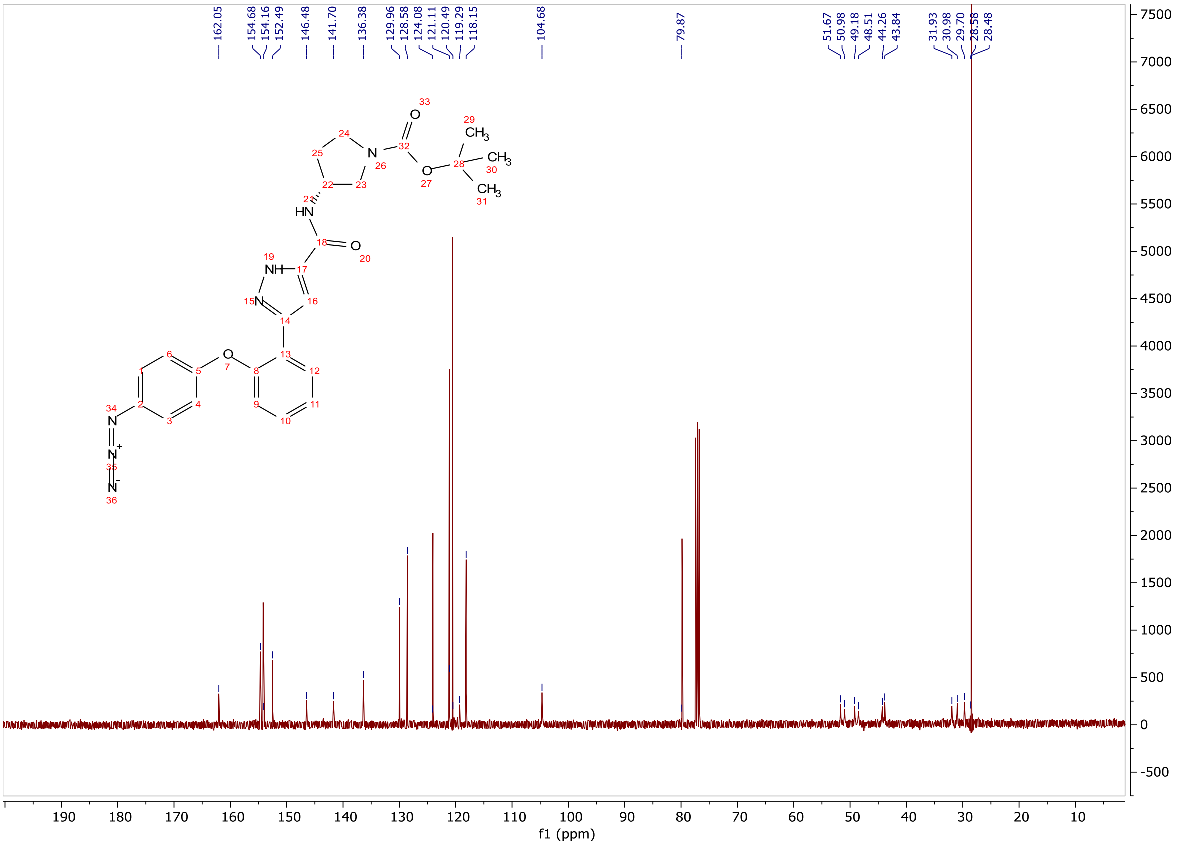

(R)-3-(2-(4-azidophenoxy)phenyl)-N-(1-cyanopyrrolidin-3-yl)-1H-pyrazole-5-carboxamide (4):

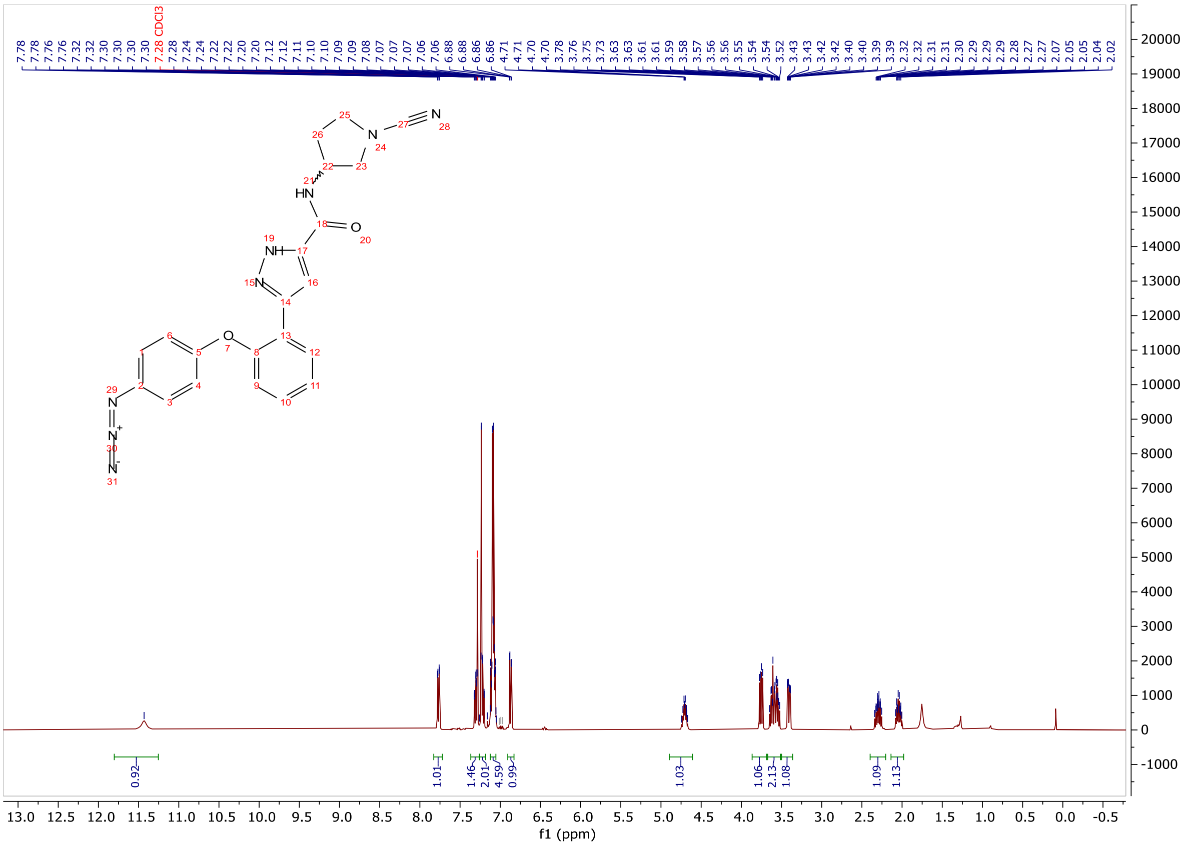

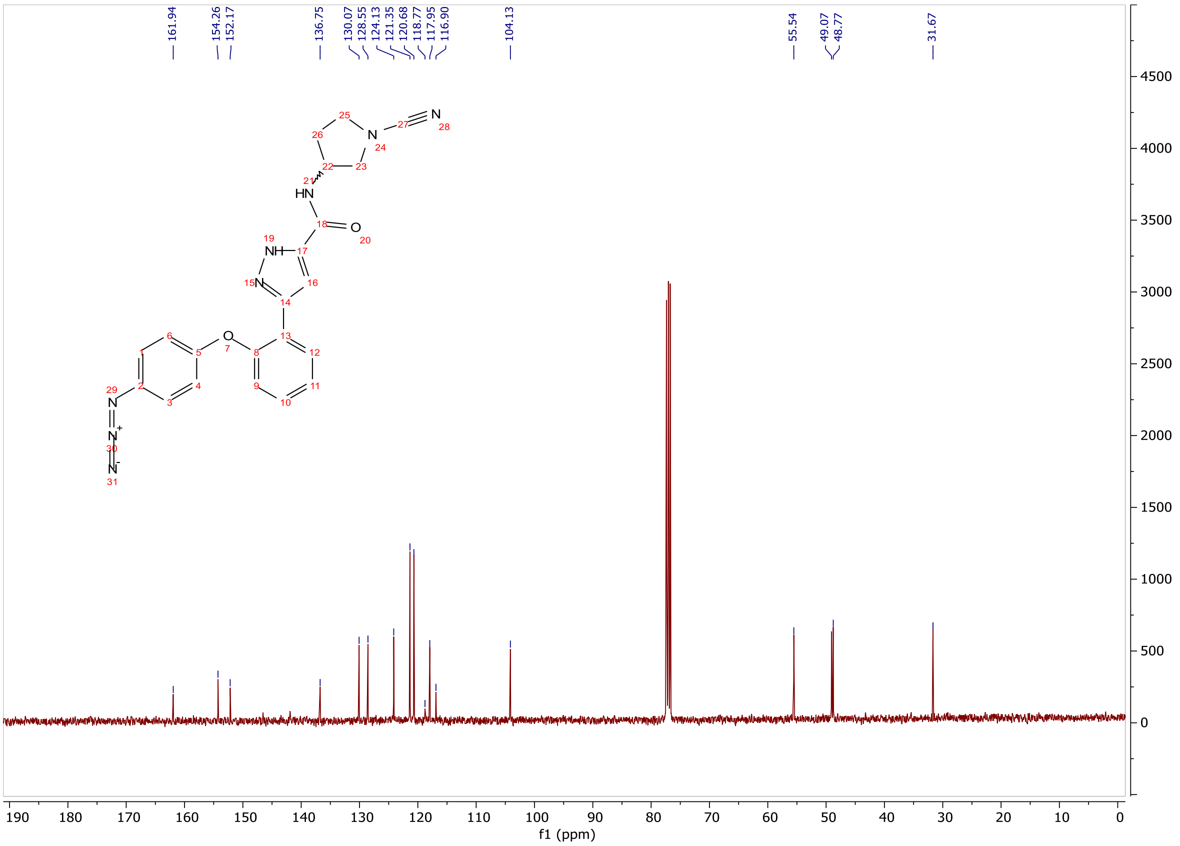

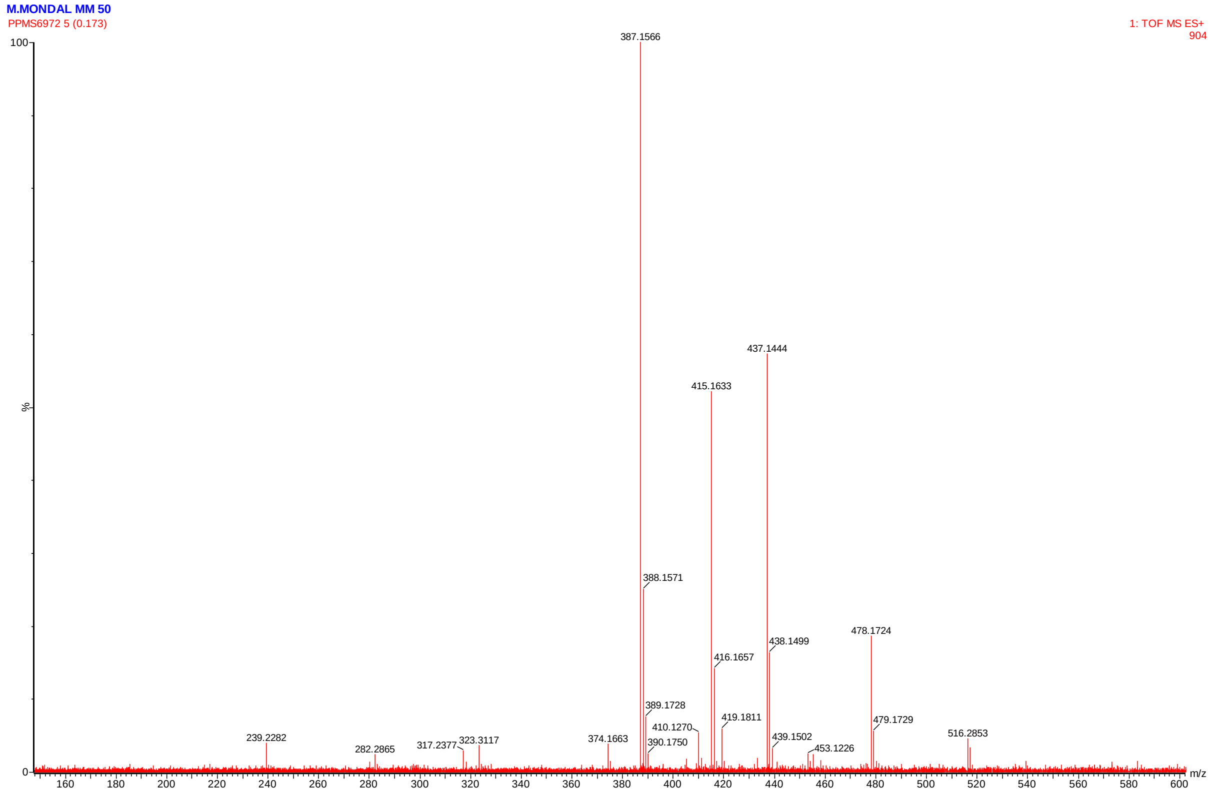
M.MONDAL MM 50
PPMS6972 5 (0.173)
1: TOF MS ES+
904
387.1566
437.1444
415.1633
388.1571
478.1724
438.1499
416.1657
389.1728
419.1811
410.1270
479.1729
239.2282
374.1663
439.1502
516.2853
323.3117
317.2377
390.1750
282.2865
453.1226
%
m/z

3-(4-chlorophenyl)isoxazol-5-amine (8a):

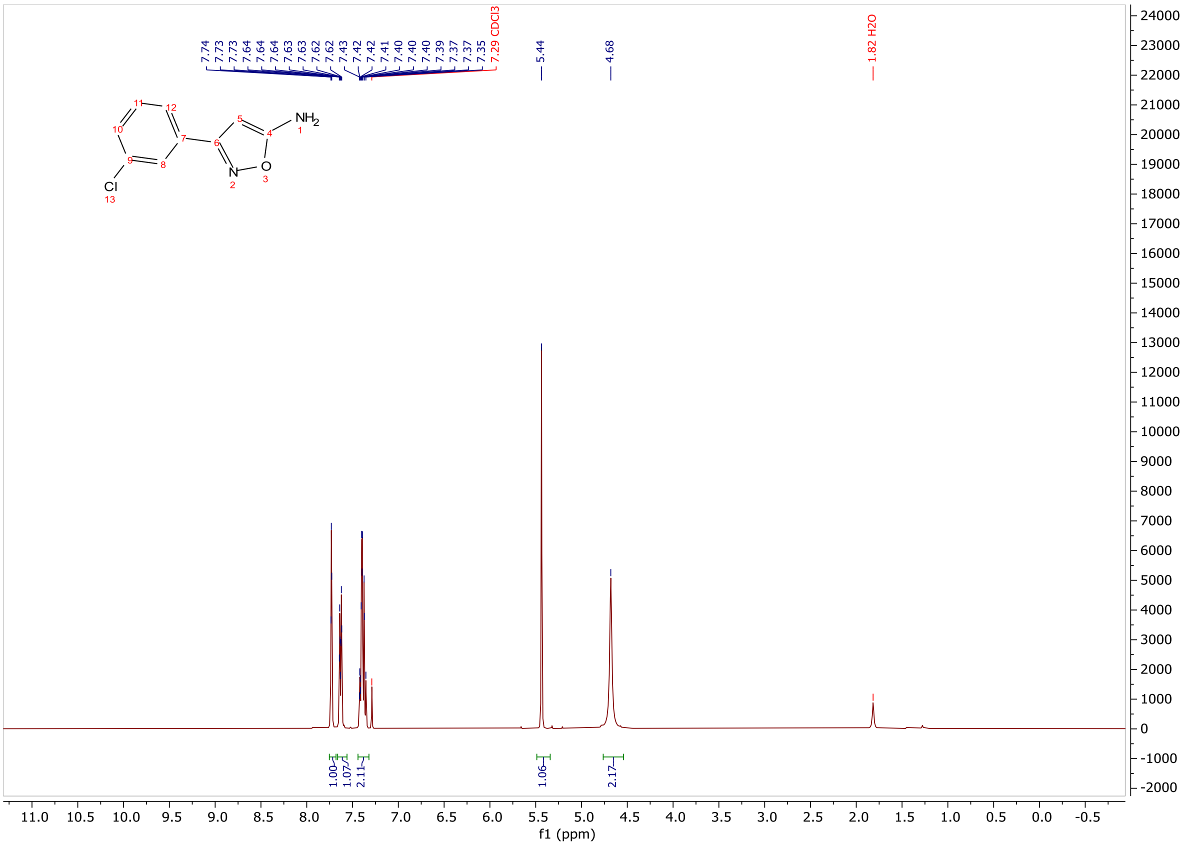

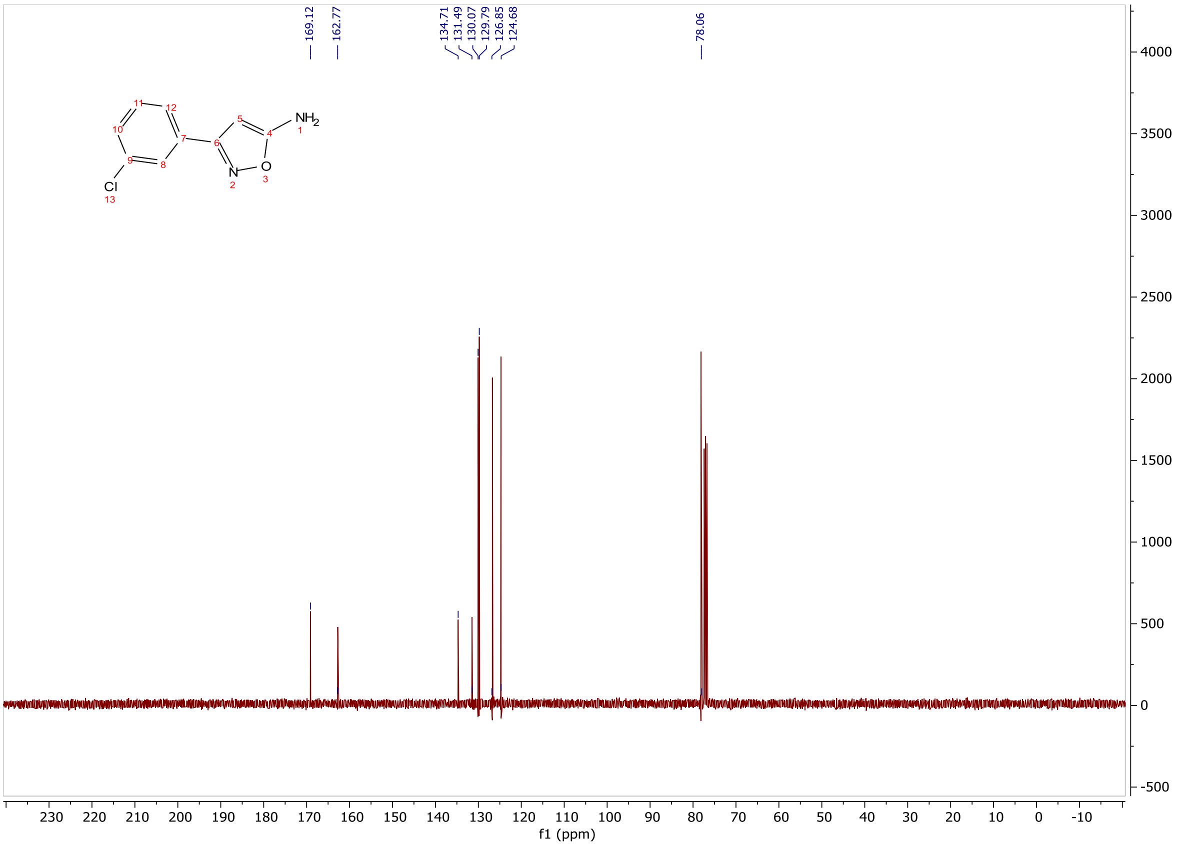

tert-butyl (R)-3-(2-((3-(3-chlorophenyl)isoxazol-5-yl)amino)-2-oxoethyl)pyrrolidine-1-carboxylate(9a):

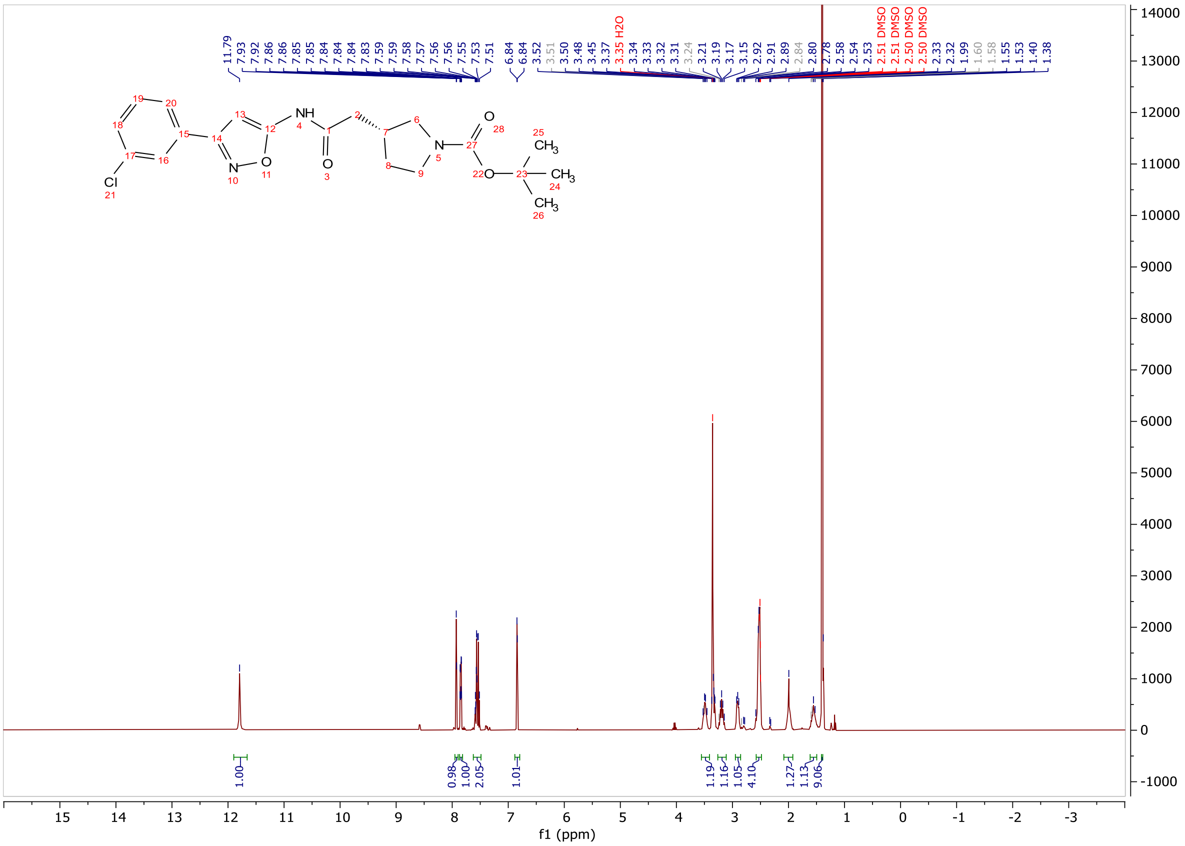

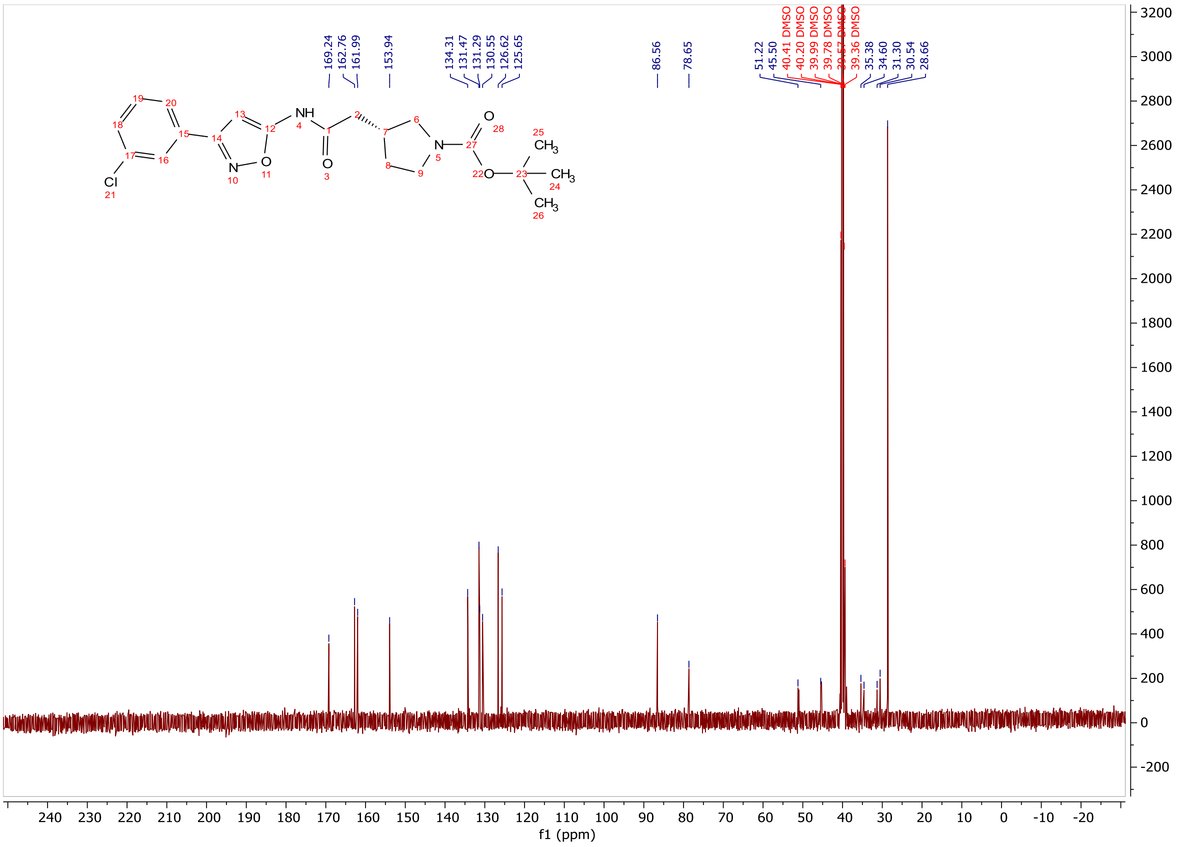

(R)-2-(1-cyanopyrrolidin-3-yl)-N-(3-(3-ethynylphenyl)isoxazol-5-yl)acetamide(1):

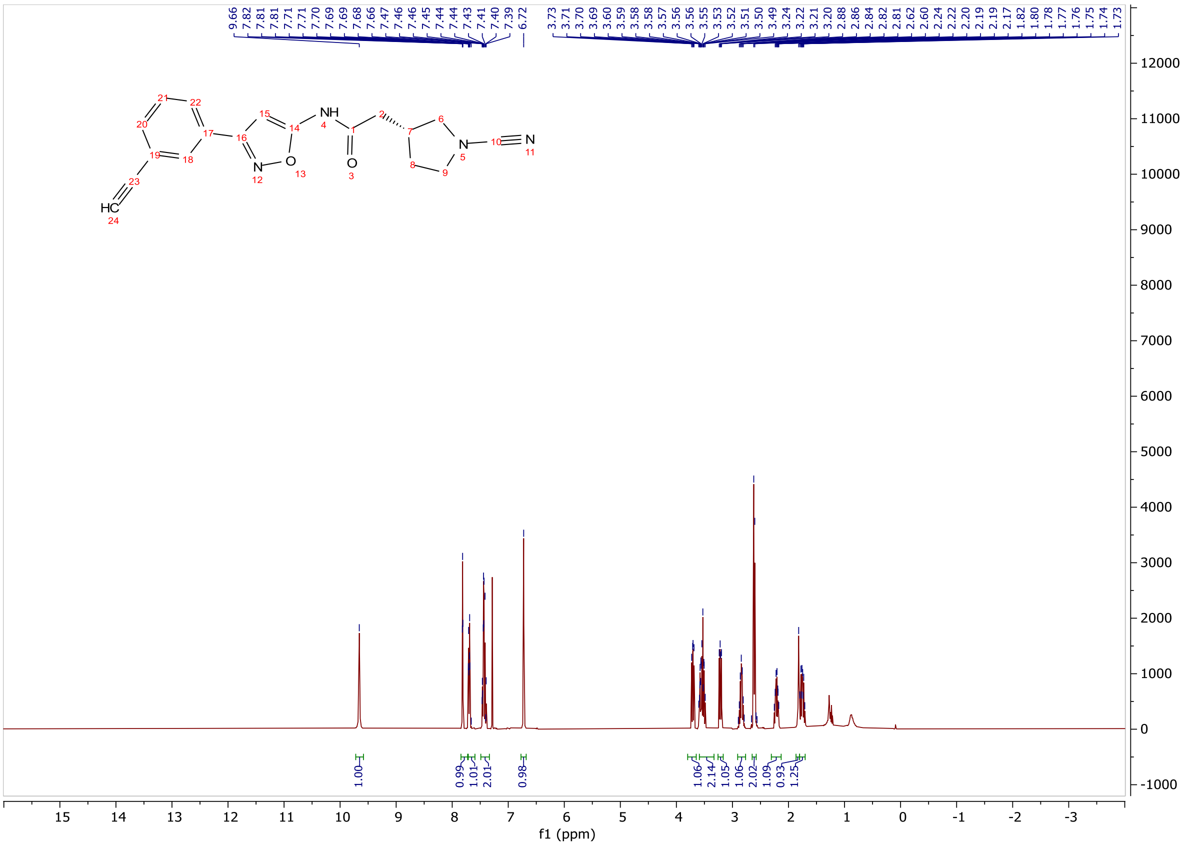

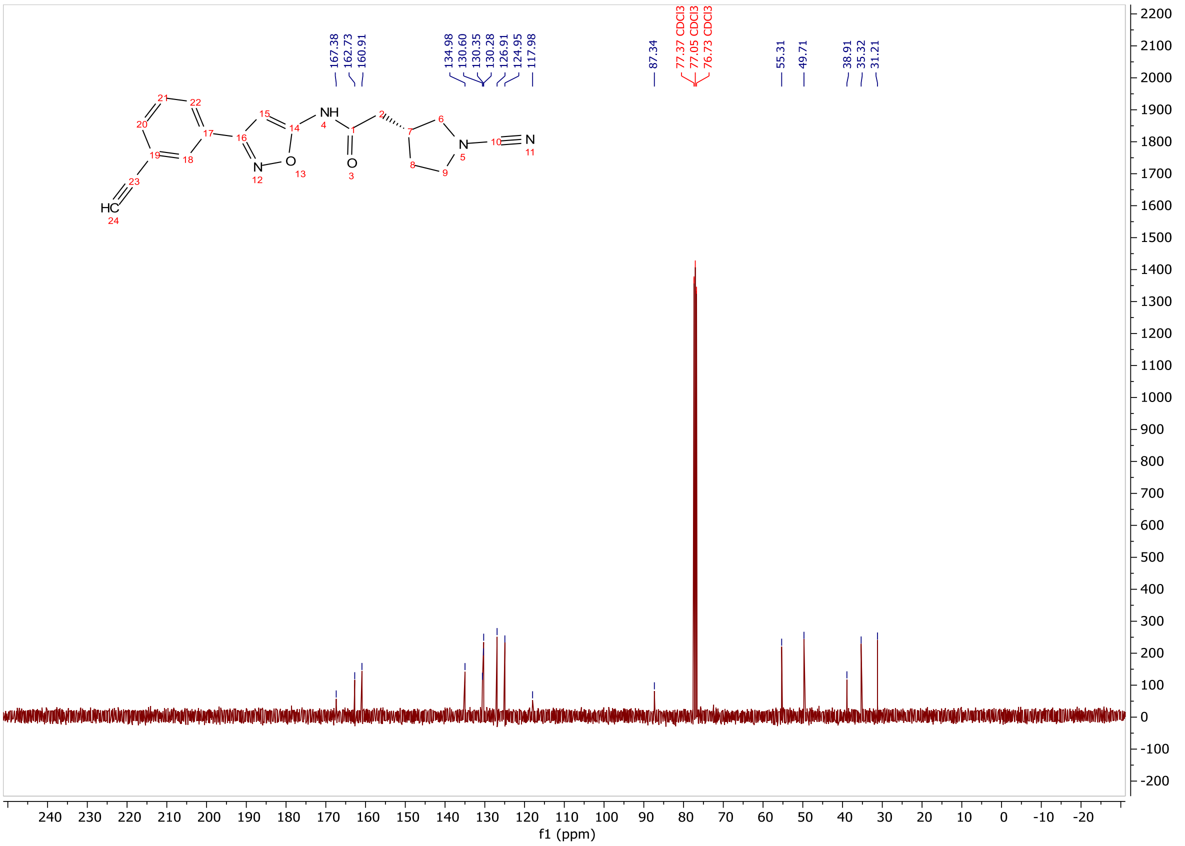

1-(2-phenoxyphenyl)ethan-1-one(13a):

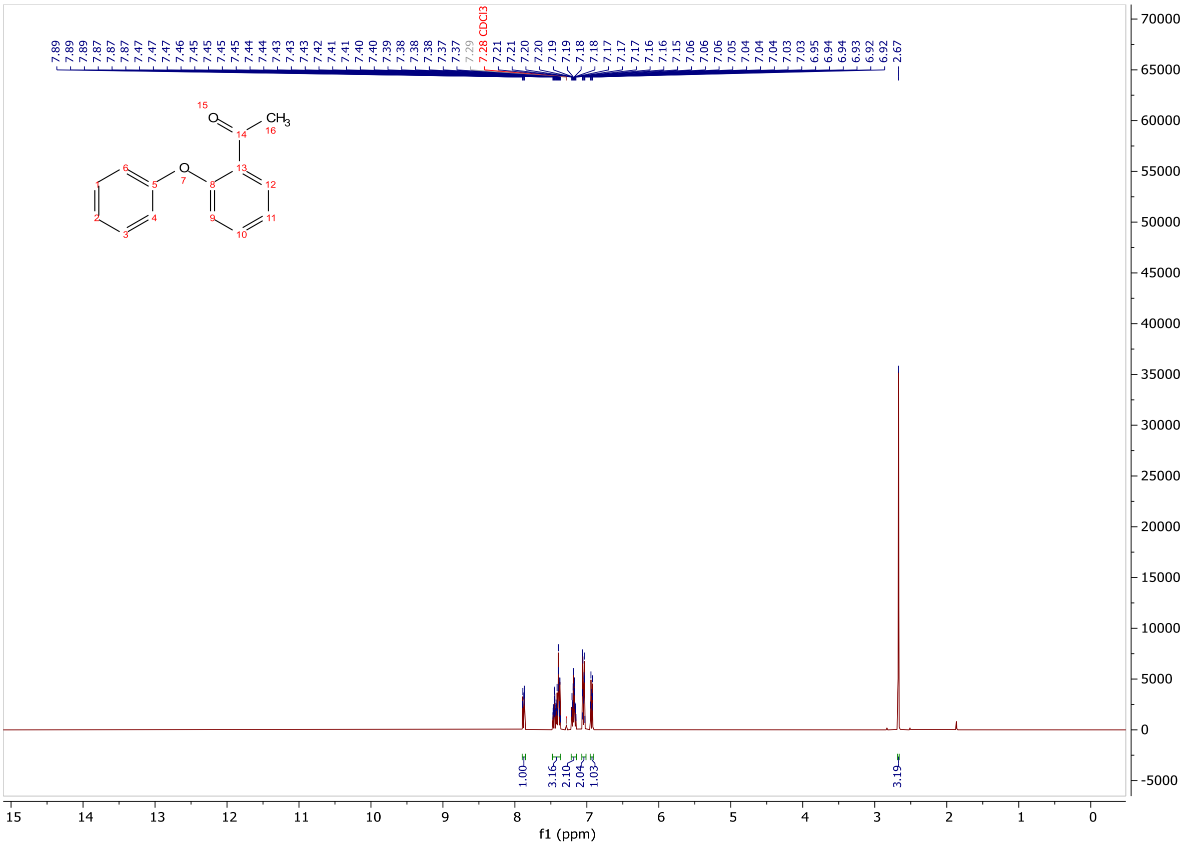

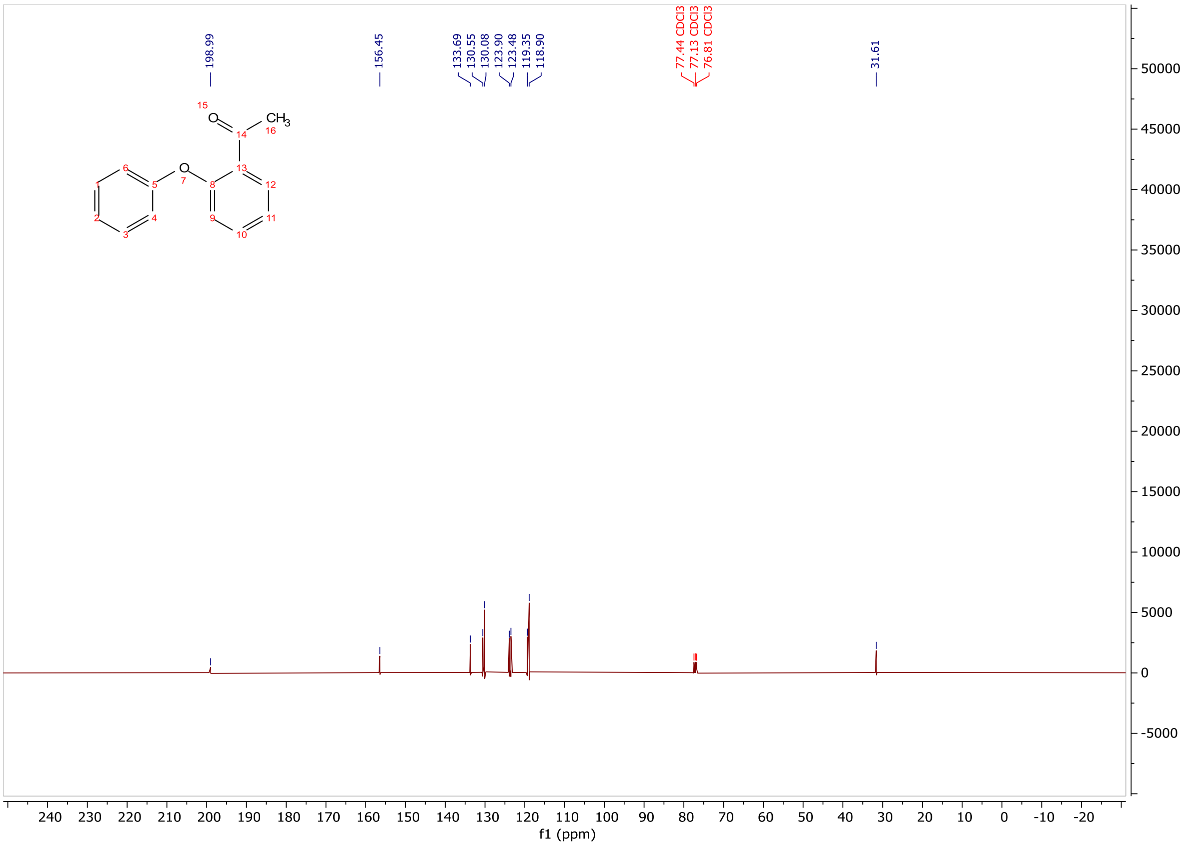

ethyl 2,4-dioxo-4-(2-phenoxyphenyl)butanoate(14a):

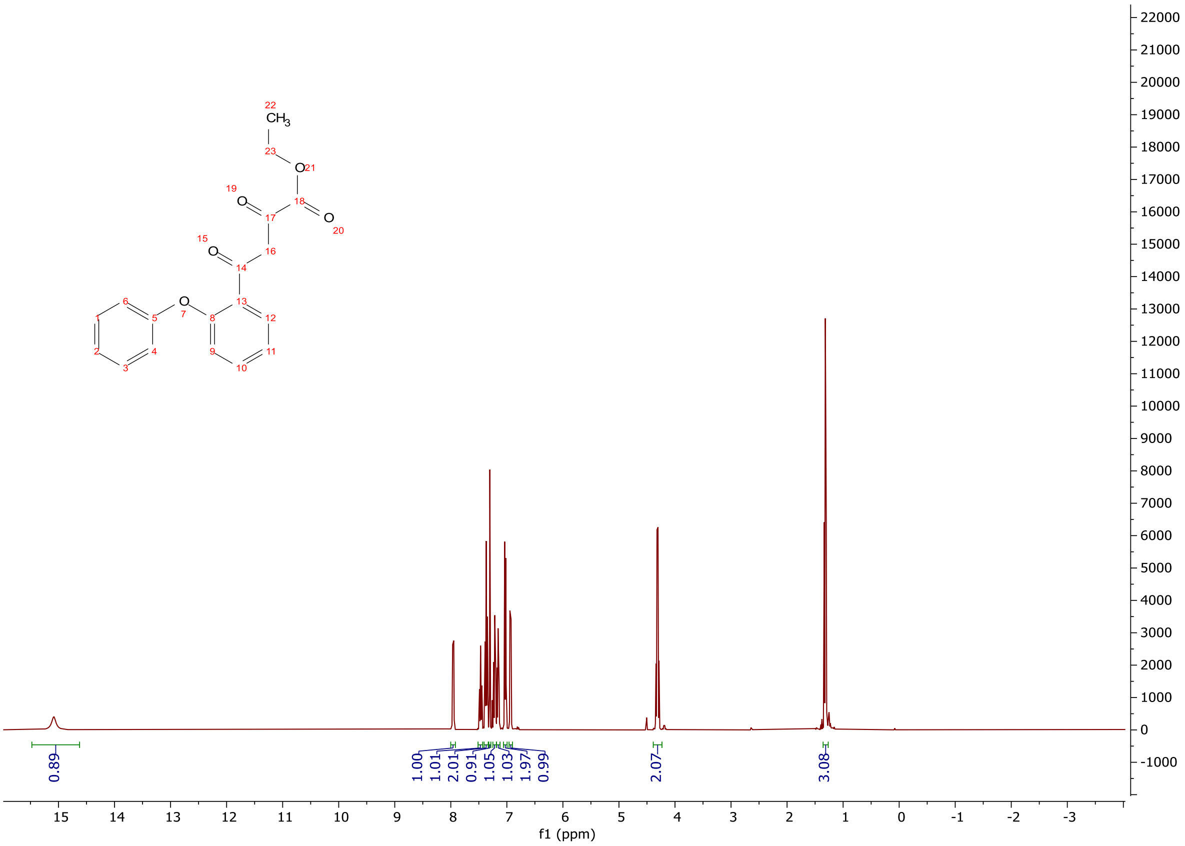

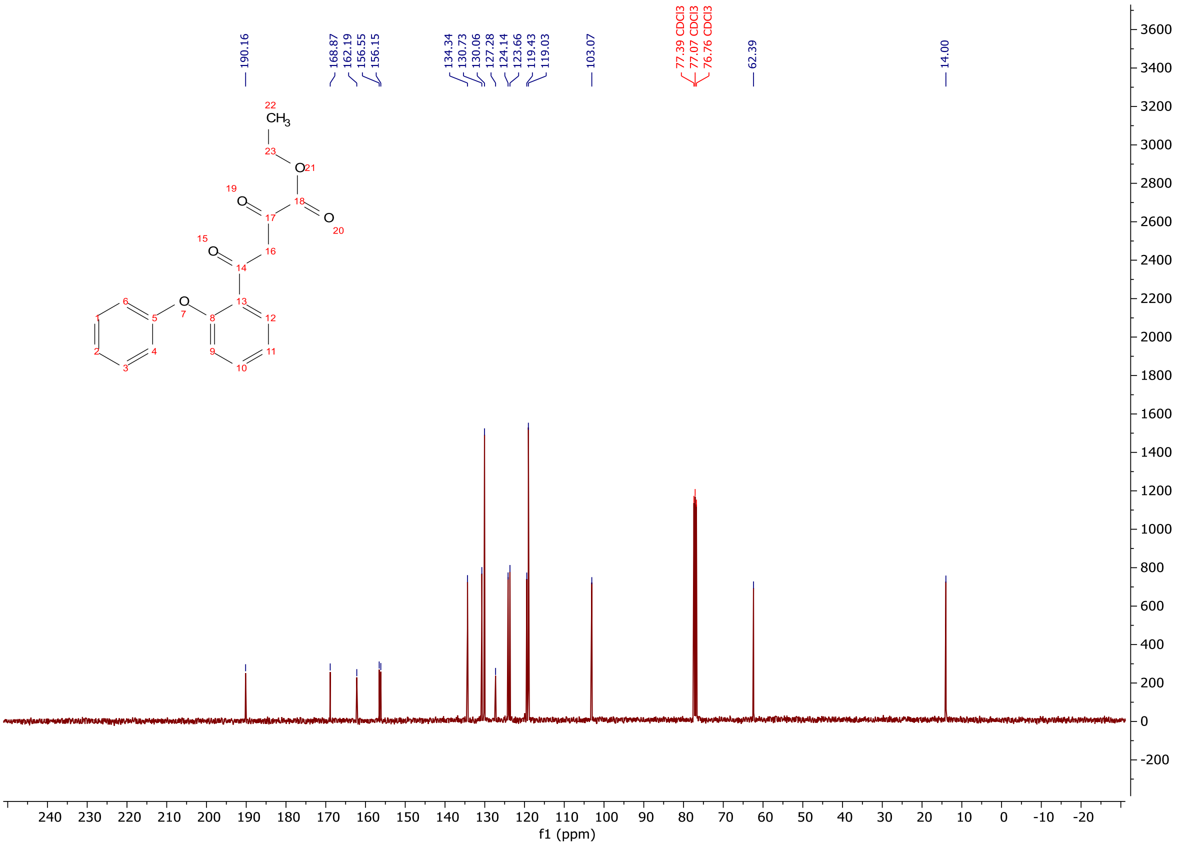

Ethyl 3-(2-phenoxyphenyl)-1H-pyrazole-5-carboxylate(15a):

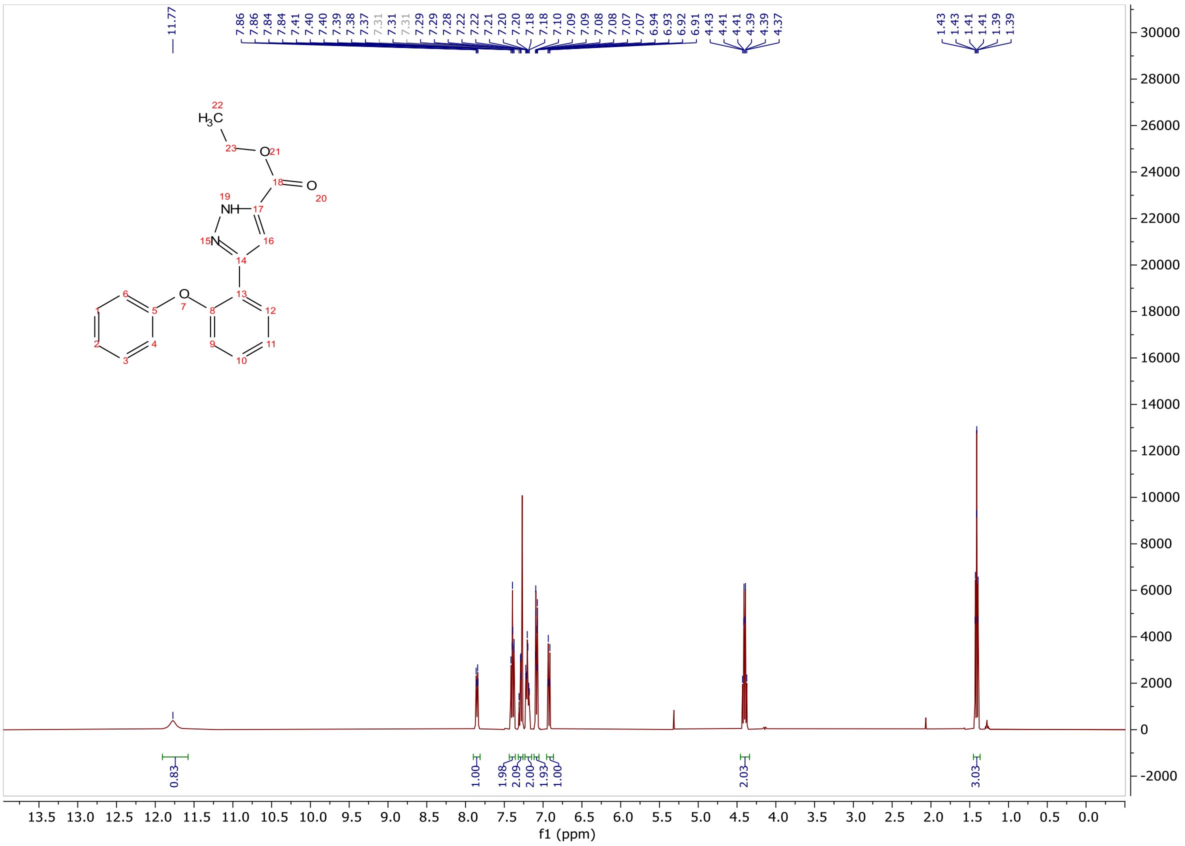

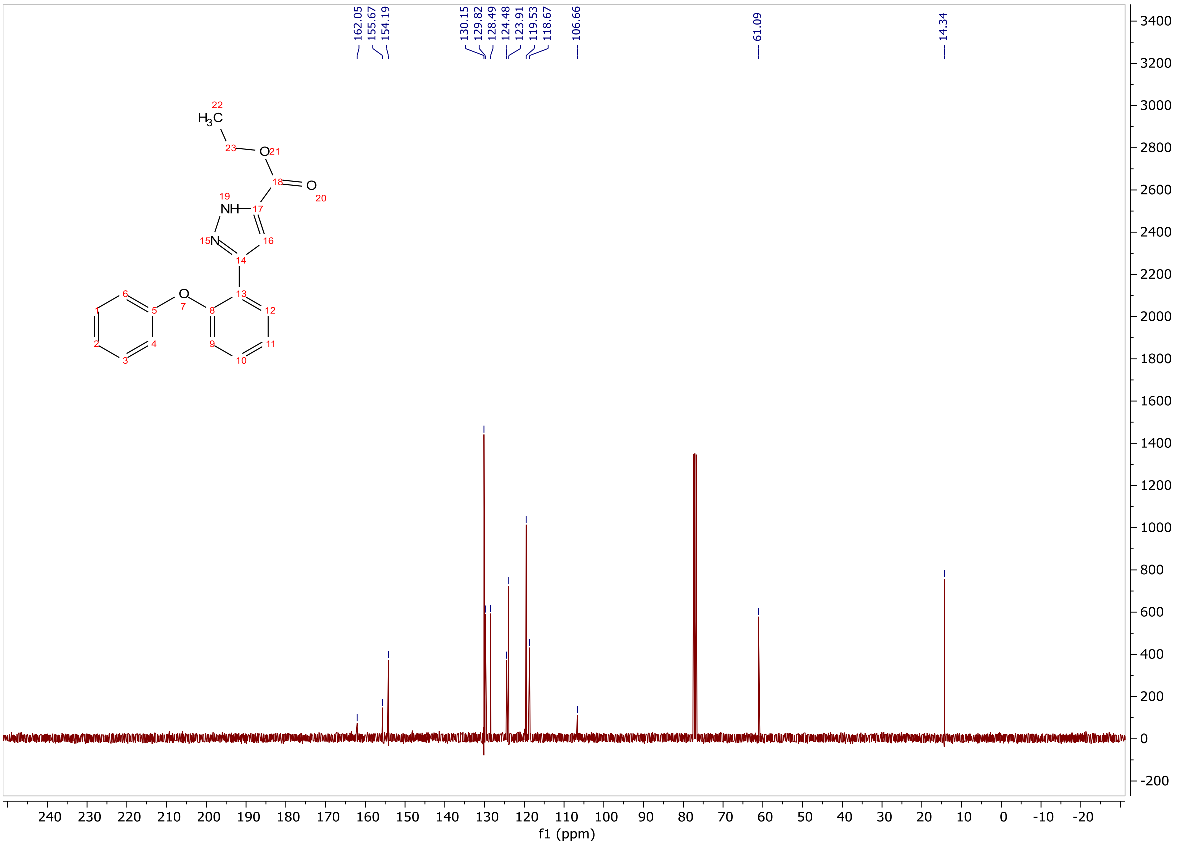

3-(2-Phenoxyphenyl)-1H-pyrazole-5-carboxylic acid(16a):

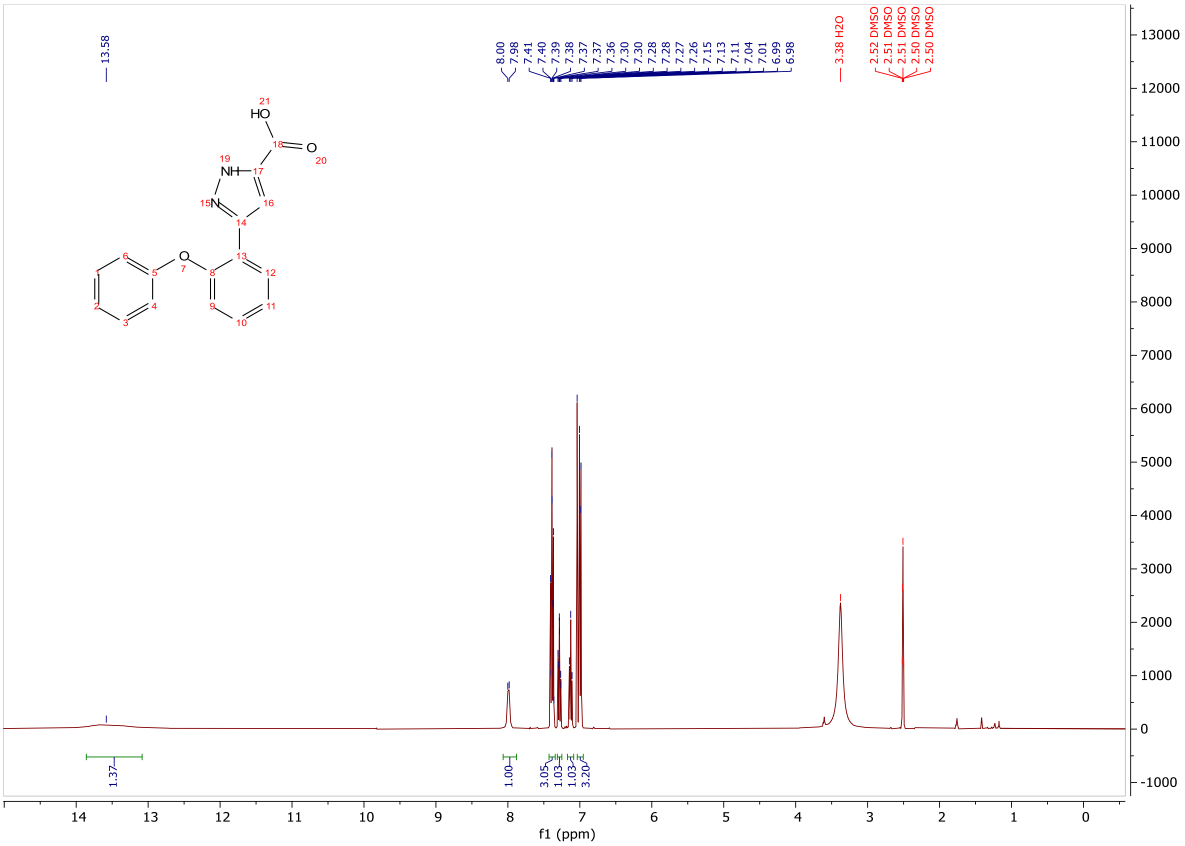

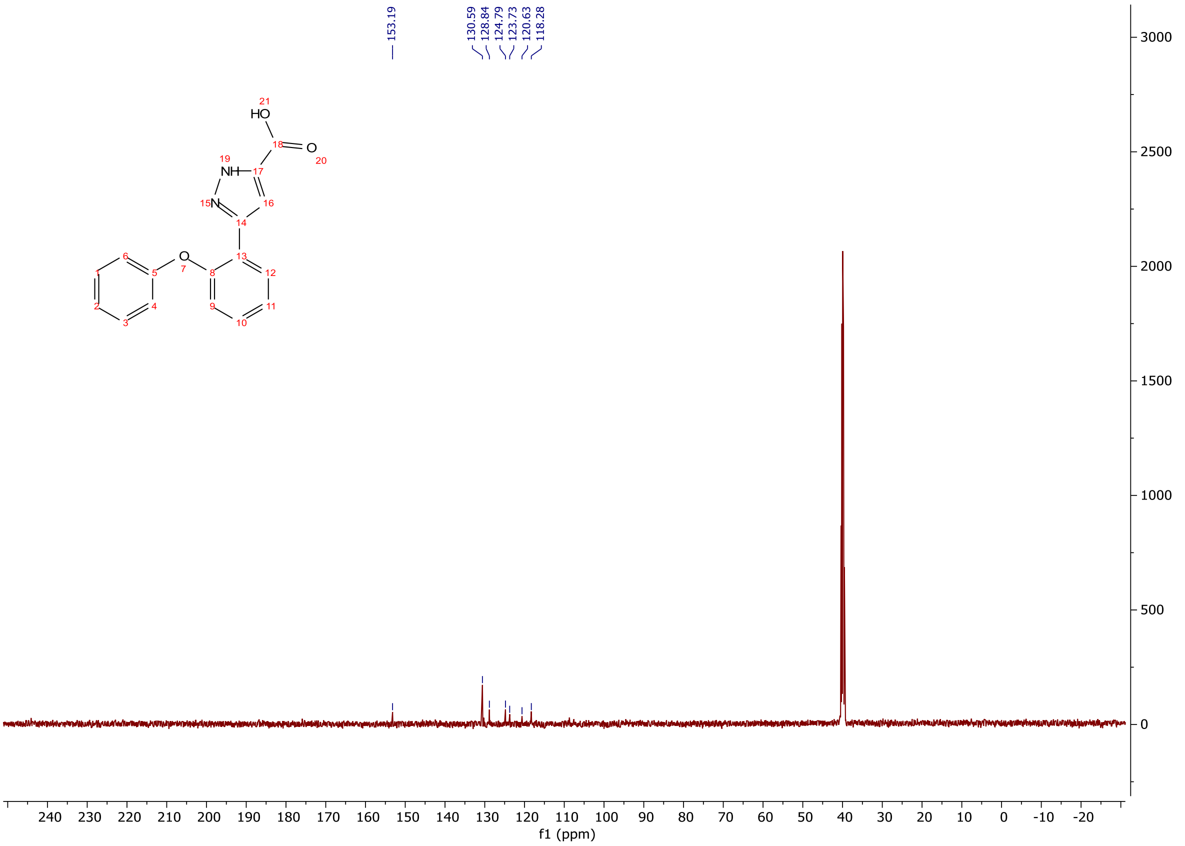

tert-Butyl (R)-3-(3-(2-phenoxyphenyl)-1H-pyrazole-5-carboxamido)pyrrolidine-1-carboxylate(18):

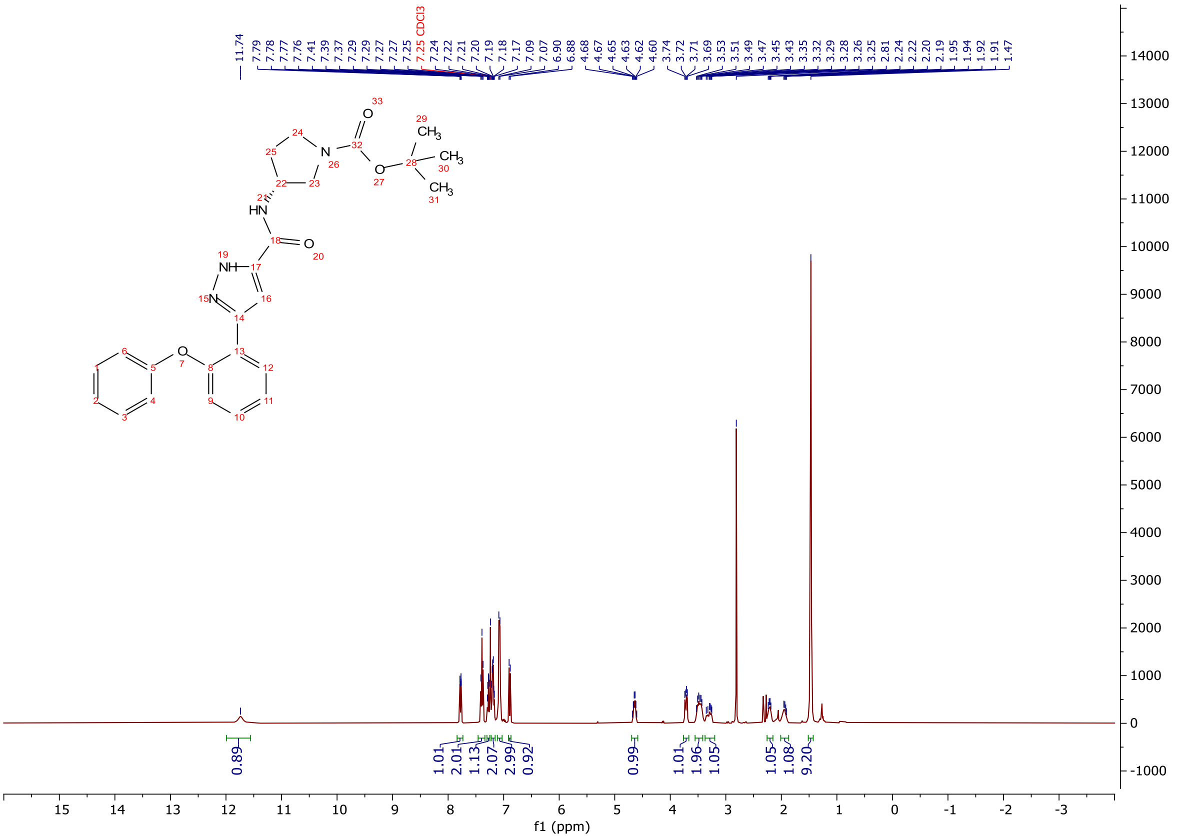

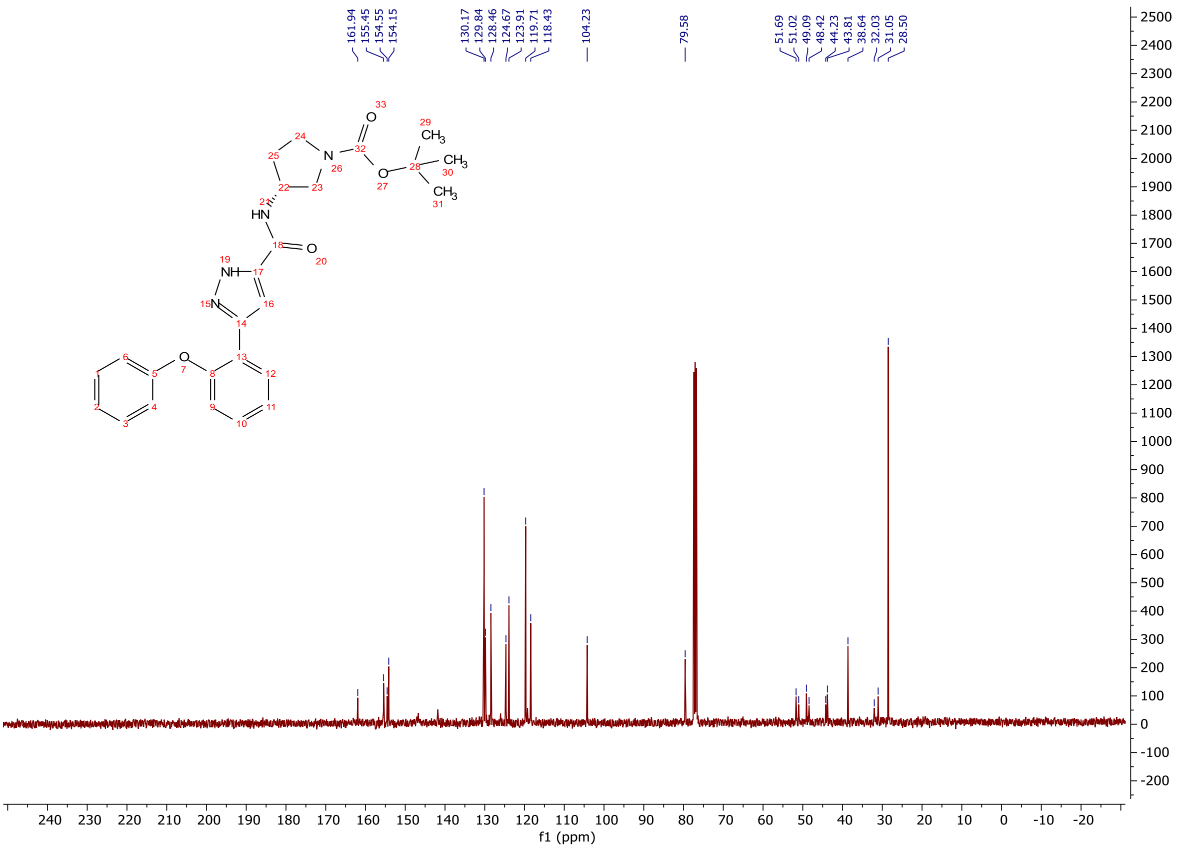

(R)-N-(1-cyanopyrrolidin-3-yl)-3-(2-phenoxyphenyl)-1H-pyrazole-5-carboxamide(3):

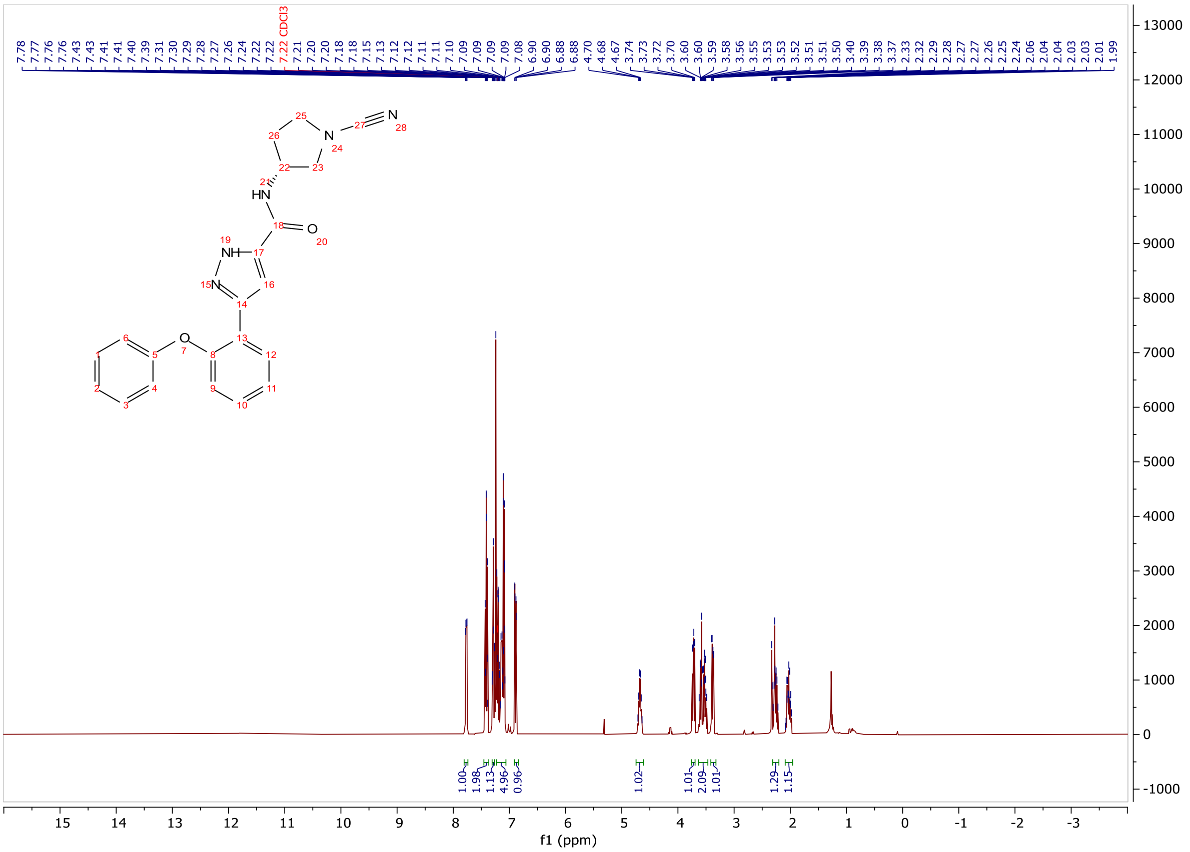

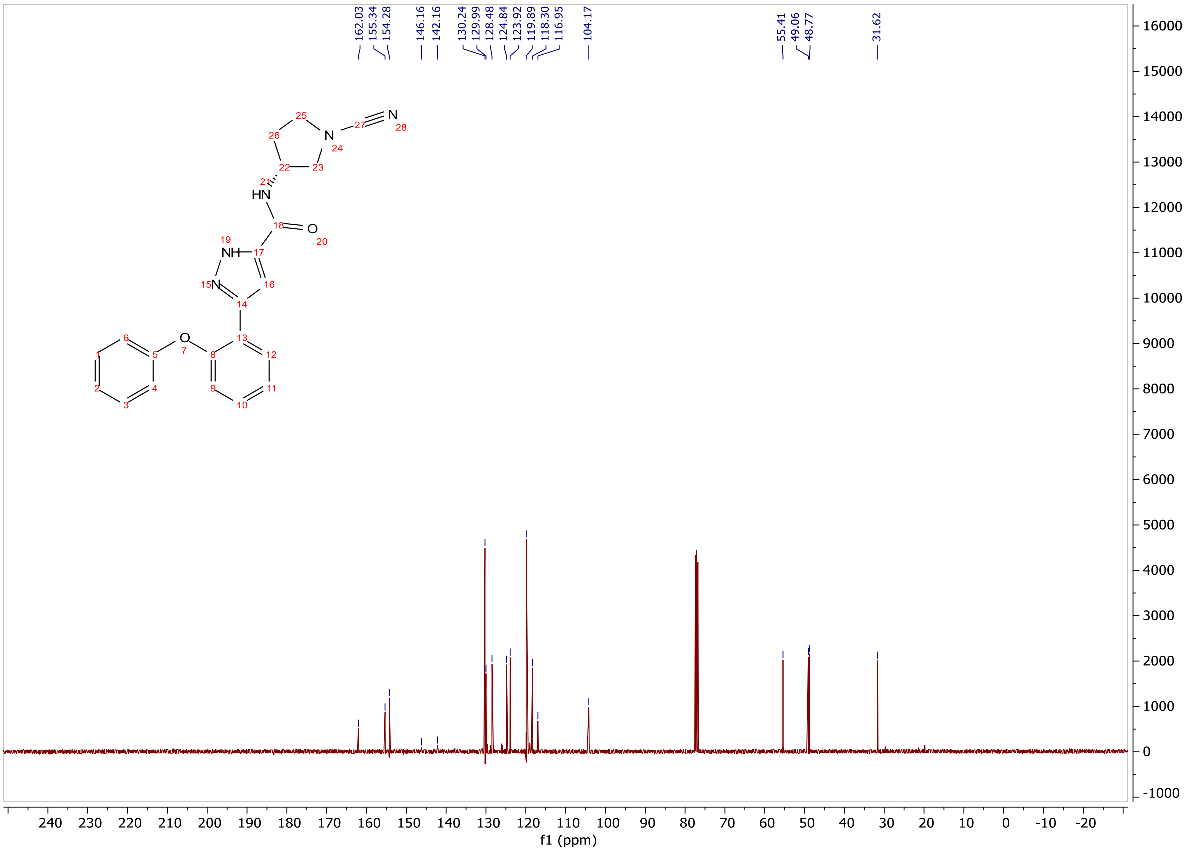

4. Biochemical and biological methods and proteomics

1) Biochemical USP30 fluorescence polarisation (FP) assay using Ub-Lys(TAMRA)-Gly

a. USP30 biochemical IC_{50} assay

Reactions were performed in triplicate in black 384 well plates (Greiner #784076) in a final reaction volume of 21 µL. Compound dilutions were prepared at 21× final concentration (2000 nM for a final concentration of 100 nM) in 50% DMSO/water. USP30 was diluted in reaction buffer A (40 mM Tris, pH 7.5, 0.005% Tween 20, 0.5 mg/ml BSA, 5 mM betamercaptoethanol) and 10 µL of diluted 10.5 nM USP30 was added to the compound (final concentration 5 nM). Enzyme and compound were incubated for 30 min at room temperature. Reactions were initiated by the addition of 10 ul 200 nM (final concentration 100 nM) of TAMRA labeled peptide linked to ubiquitin via an iso-peptide bond (Ub-Lys(TAMRA)-Gly, UbiQ-012). Reactions were read immediately after substrate addition using a Pherastar Plus (BMG Labtech; λ_{Ex} 531 nm, λ_{Em} 579 nm). IC_{50} values were determined by plotting percentage USP30 activity against compound concentration and fitting a four-parameter dose-response curve (Graphpad Prism 5.03).

b. USP30 biochemical kinetic assay

Reactions were performed in triplicate in black 384 well plates (small volume, Greiner 784076) in a final reaction volume of 20 ul. USP30 DUB catalytic domain (USP30; Catalog #E-582 residues 57-517) was diluted in reaction buffer (40 mM Tris, pH 7.5, 0.005% Tween 20, 0.5 mg/ml BSA, 5mM beta-mercaptoethanol) to the equivalent of 0, 2, 4, 20, 40 and 200 nM. 10 ul of diluted USP30 were taken in each well. Reactions were initiated by the addition of 10 ul of 40 nM of TAMRA labelled peptide linked to ubiquitin via an iso-peptide bond as fluorescence polarisation substrate. Reactions were incubated at room temperature and read every 2 min for 120 min. Readings were performed on a Pherastar Plus (BMG Labtech). l Excitation 540 nm; l Emission 590 nm.

c. Kinetic analysis

Inhibition kinetics for compound ABP **2** and ABP **4** were determined by preparing compound dilution plates at 21× final concentration in 50% DMSO in water. 50% DMSO (1 µL) or diluted compound (1 µL) was added to the reaction plate. Substrate Ub-TAMRA (200 nM, 10 µL) (final concentration: 100 nM) was prepared in buffer A described in section 3.1.a and added to the reaction plate. Reactions were initiated by the addition of USP30 (21 nM, 10 µL) (final concentration 10 nM), and fluorescence polarisation read immediately for 1 h in every 2 min. Analysis was performed in GraphPad Prism. Kinetic progress curves were fitted to equation $y = (vi/k_{obs})\ (1 - \exp(-k_{obs}*x))$ to determine the k_{obs} value (for first 30 min data). The k_{obs} value was then plotted against the inhibitor concentration to determine k_{obs}/I.

2) USP30 in-cell engagement analysis

a) Cell culture

HEK293T cells were culture in DMEM high glucose supplemented with 10% FBS. All cells were maintained in a humidified incubator at 37 °C and 5% CO_2. Cells were seeded on plates at least 16 h before experiment.

b) In-cell target engagement using HA-Ub-VME

6-well plate of HEK293T cells were incubated with media containing compounds IMP-2587 and IMP-2586 at indicated concentrations for 1 h at 37 °C. The cells were washed with PBS and lysed with RIPA buffer (Themor Scientific™ cat:89900) and cOmplete™ EDTA-free protease inhibitor cocktail (Roche). Protein concentration was determined by DC Protein assay (Bio-RAD). Lysate (20 µg protein) were incubated with HA-Ahx-Ahx-Ub-VME (0.5 µg) for 15 min at room temperature. 5× sample loading buffer (160 mM Tris pH 8, 5% (w/v) SDS, 0.025% (w/v) bromophenol blue, 25% (v/v) glycerol) containing 4% (v/v) BME was added and samples heated for 5 min at 95 °C prior to SDS-PAGE and immunoblotting.

c) SDS-PAGE and immunoblotting analysis

The prepared protein samples were separated by precast SDS-PAGE gel (10%, Bio-RAD, catalogue number:4561036) and transferred to nitrocellulose membranes (Amersham™ Protran® , GE Healthcare) by wet-tank transfer (Bio-RAD) in Tris-Glycine transfer buffer supplemented with 20% (v/v) MeOH for 1 h at 100 V. Membranes were blocked in 5% (w/v) slimmed milk in Tris-buffer (50 mM Tris pH 7.4, 150 mM NaCl) containing 0.01% (v/v) Tween-20 (TBST) for 1 h before incubation with the following primary antibody in the corresponding buffer overnight at 4°C:
UCHL1, ProteinTech, catalogue number: 14730-1-AP, 1:2000 in 5% (w/v) slimmed milk in TBST
USP30, Santa Cruz, catalogue number: sc-515235, 1:1000 in 5% (w/v) slimmed milk in TBST
HSP90, Santa Cruz, catalogue number: sc-69703, 1:1000 in 5% (w/v) slimmed milk in TBST

The membrane was washed three times with 10 mL TBST for 5 min and incubated with the corresponding HRP-conjugated secondary antibody (α-mouse-HRP or α-rabbit-HRP) in 5% (w/v) slimmed milk in TBST for 1 h at room temperature. After three washes with 10 mL TBST (10 min each), the membrane was incubated with HRP substrate (Luminata Crescendo, Millipore) and the chemiluminescence signal captured with an ImageQuant™ LAS 4000 imager. All the blots are imaged separately in the chemiluminescence channel for protein bands (HRP) and Cy5 channel for the pre-stained protein marker (Bio-RAD, Catalogue number: 1610394). The HRP channel images are processed using ImageJ software to adjust brightness and contrast (B&C) whilst ensuring that the balance between bands is preserved (figures in manuscript). The B&C adjustment parameters for each blot were displayed in supplementary information figures. The processed blots were overlayed with corresponding marker images indicating the protein molecular weight resulting in the supplementary figures.

d) Live cell probe incubation, lysate click chemistry and precipitation

6-well HEK293T cells were incubated with media containing compounds ABP **2** and ABP **4** at indicated concentrations for 1 h at 37 °C. The cells were washed with PBS and lysed with lysis buffer (50 µL (1% (v/v) Triton X-100, 0.1% (w/v) sodium dodecyl sulfate (SDS), EDTA-free complete protease inhibitor cocktail (1×, Roche) in PBS on ice for 10 min. The lysates were scraped and transferred to corresponding Lo-Bind Eppendorfs. Each lysate was sonicated for 1 minute, and the samples centrifuges at 5,000 x g at 4 ˚C for 5 minutes to pellet insoluble cellular debris. The supernatant was collected, and protein concentration was determined using the DC Protein Assay (Bio-Rad) as per manufacturer's instructions, and normalised to lowest concentrated sample (e.g. 1 mg/mL, using lysis buffer). The following "click mixture" was prepared separately, preparing 6 µL for every 100 µL of lysate:
Capture reagent (AzRB/AzT for ABP **2**, YnT/YnB for ABP **4** 10 mM in DMSO, 1 vol; final concentration in reaction 100 µM)
$CuSO_4$ (50 mM in water, 2 vol; final concentration in reaction 1 mM)
TCEP (50 mM in water, 2 vol; final concentration in reaction 1 mM)
TBTA (10 mM in DMSO, 1 vol; final concentration in reaction 100 µM)

The click mixture was vortexed and incubated at room temperature for 2 min before 6 µL of the mixture was added to every 100 µL of lysate. The reaction mixtures were shaken at room temperature for 1 h before being quenched with EDTA (500 mM in H_2O) to a final concentration of 10 mM.

Proteins were precipitated by adding H_2O (1 vol), MeOH (2 vol) and $CHCl_3$ (0.5 vol), vortexing briefly then centrifuging at 17,000 × g for 5 min. The top H_2O/MeOH layer was discarded and the middle layer of protein pellet and lower $CHCl_3$ layer retained. The suspension was washed with MeOH (300 µL), and sonicated to break up the pellet. The proteins were pelleted by centrifugation at 10,000–17000 × g for 5–10 min or until a compact pellet was formed. The protein pellet was washed once more with MeOH (1 mL). The pellet was resuspended by sonicating and completely dissolving in 1% (w/v) SDS in PBS (to 5 mg/mL protein) before being made up to 1 mg/mL protein with PBS.

e) In-gel fluorescence

The lysate samples (10-20 µg) were mixed with 4× Laemmli sample loading buffer (250 mM Tris-HCl pH 6.8, 30% (v/v) glycerol, 10% (w/v) SDS, 0.05% (w/v) bromophenol blue) supplemented with 20% v/v β-mercaptoethanol (BME) and boiled for 10 min at 95 °C. The resulting protein samples were separated according to the protocol in section 3.2.c. Gel were imaged on a Typhoon imager (GE Healthcare; λ_{Ex} 532 nm, λ_{Em} 575 nm for TAMRA. For protein loading quantification, gels were stained with Coomassie blue staining solution overnight, rinsed with water and imaged on an ImageQuant™ LAS 4000 imager (GE Healthcare).

f) Neutravidin biotin enrichment (Affinity pull-down)

Click chemistry and precipitation steps were carried out as described in section 3.2.d, on at least 400ug of protein per sample. The pellet was resuspended by sonicating and completely dissolving in 1% (w/v) SDS in HEPES 50 mM pH 8.0, before being made up to 1 mg/mL protein with HEPES 50 mM pH 8.0 to

achieve a final concentration of 0.2% SDS. 20uL of each samples was collected as TL (Total lysate) samples. Pull down of biotinylated proteins was achieved by incubating samples with pre-equilibrated (3x 1 mL washes with 0.2% (w/v) SDS in HEPES 50 mM pH 8 buffer) Pierce™ NeutrAvidin™ Agarose beads for 3 hours at room temperature. NeutrAvidin™ Agarose beads were used in ratio of 1 µL bead resin per 10ug of protein sample. The beads were subsequently washed by moderate shaking for 10 seconds then briefly pelleting by table-top centrifuge then vacuum aspirating the supernatant with fine-end pipette tips (to not disturb the agarose) after collecting 20 µL supernatant as SN (supernatant) samples. After beads wash, the samples are eluted with 2x Laemmli sample buffer containing 10% (v/v) β-mercaptoethanol at 95 °C for 10 min (PD samples, pull-down). The collected TL, SN and PD samples were analysed by SDS-PAGE and immunoblotting described in section 3.2.c. Primary antibody information were as following:
DESI2: Sigma, catalogue number: HPA049950, 1:1000 in 5% (w/v) slimmed milk in TBST
Vinculin: Abcam, catalogue number: ab91459, 1:1000 in 5% (w/v) slimmed milk in TBST
GAPDH, Abcam, catalogue number: AB9485, 1:2500 in 5% (w/v) BSA in TBST

3) Proteomics analysis

a) Live cell probe incubation

For each experiment, sterile 10 cm dishes (10 mL media working volume) were seeded with HEK293T cells and incubated at 5% CO_2 and 37◦C. After 24h, when cells had achieved 90–100% confluency, plates were treated with either DMSO or inhibitors/probes for indicated timepoint. The cells were harvested according to the description in section 3.2.d.

b) Click chemistry, precipitation, Neutravidin biotin enrichment and on-bead digestion

The harvested cell lysates were carried out by click chemistry, precipitation and enrichment described in section 3.2.d and 3.2.f. The resulting pull-downed protein on beads were reduced and alkylated with 5 mM TCEP and 10 mM chloroacetamide in 100 µL 50 mM HEPES with moderate shaking for 10 min at rt. Proteins were digested on-bead by treatment with 2.5 µL trypsin or trypsin/LysC mix(Promega, 20 ug dissolved in 100 µL 50 mM HEPES) with vigorous shaking at 37 °C overnight. The trypsin reaction was quenched by adding 1x EDTA-free protease inhibitor (50X stock,). The beads were pelleted and the supernatant (150 µL) transferred to a new epppendorf tube. An extra bead wash (50 µL, HEPES 50 mM pH 8.0) was combined with previous supernatant (200 µL total). At this stage, 10 µL of sample was analysed using a Pierce™ Quantitative Fluorometric Peptide Assay (Catalog number: 23290) as per the manufacturers instructions to determine accurate an peptide amount for TMT labelling.

c) Isobaric TMT labelling and high pH reverse phase fractionation

Upon checking that the pH of each sample was around 8.0 approximately 10 ug of peptide sample was labelled with 1/10 of an 0.8 mg vial of the appropriate TMT10plex™ Isobaric Mass Tag Labelling Reagent (Thermo Scientific) dissolved in acetonitrile (40 µL) with moderate shaking for 2 h at rt TMT-labelling was quenched by the addition of 1 µL of 5% (w/v) hydroxylamine and the samples from each TMT set were combined to form a “multi-plex” solution. Trifluoroacetic acid was added to achieve a 1% v/v solution and these samples were evaporated to dryness. Samples were then fractionated using

the Pierce High pH Reversed-Phase Peptide Fractionation Kit (Catalog number: 84868) as per the manufacturers instructions. All fractions of each sample were collected in low-bind epppendorfs tubes, evaporated to dryness and stored at –80 °C.

d) LC-MS/MS analysis

Samples were rehydrated in 0.5% (v/v) formic acid, 2% (v/v) UPLC grade MeCN in Optima™ LC/MS H_2O (Fisher Scientific) and dissolved completely by vortexing and sonication. Samples were filtered through 3x Durapore® membrane filters (Millipore) plugged into a p20 pipette tip by centrifuging the samples through the filters (4000 × g, 5 min) into a mass spectrometry vial. Samples were stored at 4 °C until ready for analysis.

Peptides were separated on an EASY-Spray™ Acclaim PepMap C18 column (50 cm × 75 µm inner diameter, Thermo Fisher Scientific) using a 3-hour linear gradient separation of 0–100% solvent B (80% MeCN supplemented with 0.1% formic acid): solvent A (2% MeCN supplemented with 0.1% trifluoroacetic acid) at a flow rate of 250 nL/min. The liquid chromatography was coupled to a Q-Exactive mass spectrometer via an easy-spray source (Thermo Fisher Scientific) which operated in data-dependent mode with survey scans acquired at a resolution of 70,000 at m/z 200. Scans were acquired from 350 to 1800 *m/z*. Up to 10 of the most abundant isotope patterns with charge +2 or higher from the survey scan were selected with an isolation window of 1.6 m/z and fragmented by HCD with normalized collision energy of 25. The maximum ion injection times for the survey scan and the MS/MS scans (acquired with a resolution of 35,000 at *m/z* 200) were 20 and 120 ms, respectively. The ion target value for MS was set to 106 and for MS/MS to 105, and the intensity threshold was set to 8.3×10^2.

e) Database searching and proteomics data analysis

Peptide searches were performed in MaxQuant (version 1.6.10.43). Under group-specific parameters and type, reporter ion MS2 was selected, and the appropriate TMT10plex™ isobaric labels selected for both lysines and N-termini. The isotope errors contained in each TMT batch code was also entered. For all experiments, oxidation (M) and acetyl (protein N-term) were set as variable modifications, carbamidomethyl (C) was set as a fixed modification, trypsin/P was set as the digestion mode. Where multiple TMT sets were analysed, re-quantify and match between runs were selected, and latest UniProt FASTA files for the human proteome and contaminants databases were used.

Data analysis was performed in Perseus version 1.16.6.0. Reporter intensity corrected values were loaded into the matrix. Data was filtered by removing rows based on “reverse”, and “potential contaminant” columns. Data were log2 transformed and filtered by row, retaining those that had 2 valid values in each triplicate condition. To account for variance in protein abundance across different sample, the median of each channel was subtracted from each protein. If appropriate, multiple TMT data sets were normalized by subtracting the mean of each row within each TMT “plex”. The log2 fold enrichment for each probe was determined by subtracting the DMSO control value from each of the different probe treated conditions.

4) USP30 WT and catalytic CS overexpressed in HEK293T cells

a) Preparation of DNA constructs

USP30 WT (#22578) were purchased from Addgene[4]. USP30 C77S was generated by PCR using Site-directed Mutagenesis Analysis kit (NEB) and the following primers:

USP30 C77S forward: 5’-GGGAACACCTCCTTCATGAAC-3’

USP30 C77S reverse: 5’-TAAATTAACAAGGCCAGG-3’

b) Profiling labelling in USP30 overexpression cells

HEK293T cells were transfected with plasmid encoding for USP30 WT and USP30 C77S using Lipofectamine™ 2000 Transfection Reagent (Invitrogen) and Opti-MEM™ reduced serum medium (GibCO™) according to the manufacturer’s instruction with the following modifications. USP30 WT (2.5 µg per well) and USP30 C77S (2.5 µg per well) was mixed with lipofectamine (3 µL per well) and added to cells in a 6-well plate. After 24h transfection, medium was removed and replaced by fresh culture medium supplemented with DMSO or inhibitors with indicated concentration for 1h. Then, probes with indicated concentration were added for another 1h treatment. After cell lysis, samples were CuAAC ligation and analyzed by western blotting described in Section 3.2.f. Primary antibody information were as following:

HA, Cell signalling, catalogue number: C29F4, 1:1000 in 5% (w/v) slimmed milk in TBST

5. Reference

1. M. L. Stockley; M. I. Kemp; A. Madin; M. D. Woodrow; A. Jones. Cyanopyrolidine Derivatives with Activity as Inhibitors of USP30. WO2018/060689 A1, 2018.

2. Rusilowicz-Jones EV, Jardine J, Kallinos A, Pinto-Fernandez A, Guenther F, Giurrandino M, Barone FG, McCarron K, Burke CJ, Murad A, Martinez A, Marcassa E, Gersch M, Buckmelter AJ, Kayser-Bricker KJ, Lamoliatte F, Gajbhiye A, Davis S, Scott HC, Murphy E, England K, Mortiboys H, Komander D, Trost M, Kessler BM, Ioannidis S, Ahlijanian MK, Urbé S, Clague MJ. USP30 sets a trigger threshold for PINK1-PARKIN amplification of mitochondrial ubiquitylation. Life Sci Alliance. 2020 Jul 7;3(8):e202000768. doi: 10.26508/lsa.202000768.

3. Panyain N, Godinat A, Thawani AR, Lachiondo-Ortega S, Mason K, Elkhalifa S, Smith LM, Harrigan JA, Tate EW. Activity-based protein profiling reveals deubiquitinase and aldehyde dehydrogenase targets of a cyanopyrrolidine probe. RSC Med Chem. 2021 Aug 16;12(11):1935-1943.

4. Sowa ME, Bennett EJ, Gygi SP, Harper JW. Defining the human deubiquitinating enzyme interaction landscape. *Cell.* 2009 Jul 23. 138(2):389-403.
